# A non-coding RNA balancing act: miR-346-induced DNA damage is limited by the long non-coding RNA NORAD in prostate cancer

**DOI:** 10.1186/s12943-022-01540-w

**Published:** 2022-03-22

**Authors:** C. E. Fletcher, L. Deng, F. Orafidiya, W. Yuan, M. P. G. S. Lorentzen, O. W. Cyran, A. Varela-Carver, T. A. Constantin, D. A. Leach, F. M. Dobbs, I. Figueiredo, B. Gurel, E. Parkes, D. Bogdan, R. R. Pereira, S. G. Zhao, A. Neeb, F. Issa, J. Hester, H. Kudo, Y. Liu, Y. Philippou, R. Bristow, K. Knudsen, R. J. Bryant, F. Y. Feng, S. H. Reed, I. G. Mills, J. de Bono, C. L. Bevan

**Affiliations:** 1 Imperial Centre for Translational and Experimental Medicine, Department of Surgery & Cancer, Imperial College London, London, UK; 2 Institute of Cancer Research and The Royal Marsden NHS Foundation Trust, Sutton, UK; 3 Division of Cancer and Genetics, School of Medicine, Cardiff University, Heath Park, Cardiff, UK; 4 Broken String Biosciences, Unit AB303, Level 3, BioData Innovation Centre, Wellcome Genome Campus, Hinxton, Cambridge, UK; 5 Institute for Radiation Oncology, Department of Oncology, University of Oxford, London, UK; 6 Translational Oncogenomics, Manchester Cancer Research Centre and Cancer Research UK Manchester Institute, Manchester, UK; 7 Division of Cancer Sciences, Faculty of Biology Medicine and Health, University of Manchester, Manchester, UK; 8 Department of Human Oncology, University of Wisconsin School of Medicine and Public Health, University of Wisconsin-Madison, Madison, WI USA; 9 Transplantation Research and Immunology Group, Nuffield Department of Surgical Sciences, University of Oxford, Oxford, UK; 10 Section of Pathology, Department of Metabolism, Digestion and Reproduction, Imperial College London, London, UK; 11 Veracyte, Inc., San Diego, CA USA; 12 Nuffield Department of Surgical Sciences, University of Oxford, John Radcliffe Hospital, Oxford, UK; 13 Christie NHS Foundation Trust, Manchester, UK; 14 Department of Cancer Biology, Thomas Jefferson University, Philadelphia, PA USA; 15 American Cancer Society and American Cancer Society Cancer Action Network, Washington DC, USA; 16 Departments of Urology and Radiation Oncology, University of California San Francisco, San Francisco, CA USA; 17 Patrick G Johnston Centre for Cancer Research, Queen’s University of Belfast, Belfast, UK; 18 Centre for Cancer Biomarkers, University of Bergen, Bergen, Norway; 19 Department of Clinical Science, University of Bergen, Bergen, Norway

**Keywords:** Non-coding RNA, Prostate cancer, DNA damage, microRNA, Long non-coding RNA, Replication stress, Target-directed microRNA decay, cancer

## Abstract

**Background:**

miR-346 was identified as an activator of Androgen Receptor (AR) signalling that associates with DNA damage response (DDR)-linked transcripts in prostate cancer (PC). We sought to delineate the impact of miR-346 on DNA damage, and its potential as a therapeutic agent.

**Methods:**

RNA-IP, RNA-seq, RNA-ISH, DNA fibre assays, in vivo xenograft studies and bioinformatics approaches were used alongside a novel method for amplification-free, single nucleotide-resolution genome-wide mapping of DNA breaks (INDUCE-seq).

**Results:**

miR-346 induces rapid and extensive DNA damage in PC cells - the first report of microRNA-induced DNA damage. Mechanistically, this is achieved through transcriptional hyperactivation, R-loop formation and replication stress, leading to checkpoint activation and cell cycle arrest. miR-346 also interacts with genome-protective lncRNA NORAD to disrupt its interaction with PUM2, leading to PUM2 stabilisation and its increased turnover of DNA damage response (DDR) transcripts. Confirming clinical relevance, NORAD expression and activity strongly correlate with poor PC clinical outcomes and increased DDR in biopsy RNA-seq studies. In contrast, miR-346 is associated with improved PC survival.

INDUCE-seq reveals that miR-346-induced DSBs occur preferentially at binding sites of the most highly-transcriptionally active transcription factors in PC cells, including c-Myc, FOXA1, HOXB13, NKX3.1, and importantly, AR, resulting in target transcript downregulation. Further, RNA-seq reveals widespread miR-346 and shNORAD dysregulation of DNA damage, replication and cell cycle processes.

NORAD drives target-directed miR decay (TDMD) of miR-346 as a novel genome protection mechanism: NORAD silencing increases mature miR-346 levels by several thousand-fold, and WT but not TDMD-mutant NORAD rescues miR-346-induced DNA damage. Importantly, miR-346 sensitises PC cells to DNA-damaging drugs including PARP inhibitor and chemotherapy, and induces tumour regression as a monotherapy in vivo, indicating that targeting miR-346:NORAD balance is a valid therapeutic strategy.

**Conclusions:**

A balancing act between miR-346 and NORAD regulates DNA damage and repair in PC. miR-346 may be particularly effective as a therapeutic in the context of decreased NORAD observed in advanced PC, and in transcriptionally-hyperactive cancer cells.

**Supplementary Information:**

The online version contains supplementary material available at 10.1186/s12943-022-01540-w.

## Background

Prostate cancer (PC) is the second commonest cancer and a leading cause of male cancer death [1]. The Androgen Receptor (AR), a member of the nuclear receptor family of transcription factors, is essential to PC development and disease progression, even in metastatic ‘castration-resistant’ PC (CRPC). Androgen-deprivation therapy (ADT) is therefore standard-of-care for both localised PC and CRPC [2]. Eventually, however, resistance to ADT necessitates alternative therapeutic approaches.

Cross-talk between AR signalling and DNA damage response (DDR) is now well-appreciated [3]: DNA damage activates AR signaling with the latter promoting DDR gene transcription and DNA repair [3–5]. CRPCs are reported to accumulate defective DNA repair and cell cycle checkpoints [6–8], with 20–30% CRPC having deleterious aberrations in homologous recombination (HR)-mediated DNA repair [9, 10]; microsatellite instability (MSI) has also been reported in 1% of primary tumours and 4–12% of CRPC [11–15]. Importantly, such defects create vulnerabilities that can be exploited therapeutically: HR-deficient tumours [16–18] are sensitive to PARP inhibition (PARPi), and MSI high tumours can respond to immunotherapy. Alternative DDR-targeting approaches thus represent promising therapeutic strategies for CRPC.

Non-coding RNA (ncRNA) constitutes almost 70% of the human transcriptome [19]. Despite their large numbers and robust demonstrations of the biological significance of ncRNA in cancer progression including prostate cancer [20], these molecules remain under-studied. ncRNAs can be divided into long non-coding RNAs (lncRNAs, ≥ 200 nt) and small RNAs. microRNAs (miRs) constitute a disease-relevant subset of small ncRNAs that modulate gene expression through association predominantly with the 3’UTR of longer transcripts. In most cases, this results in transcript degradation and/or translational inhibition. miRs are reported to be dysregulated during PC progression and show promise as biomarkers and therapeutic targets, with several in cancer clinical trials [21–25]. LncRNAs show remarkable sequence and structural diversity and are key regulators of gene activity, functioning variously as transcriptional repressors/enhancers, miR sponges, competitive endogenous RNAs, enhancer RNAs, splicing modulators and scaffolds. They show a high degree of tissue-specificity, and several have well-established PC roles: for example, lncRNA PCA3 is FDA-approved as a urinary diagnostic PC biomarker [26].

Previous work from our laboratory identified miR-346 as increasing AR activity through transcript stabilisation [27]. Pathway analysis of experimentally-validated miR-346 targets identified roles for miR-346 in DNA replication, DNA damage and cell cycle regulation, and mining of PC AGO-PAR-CLIP-seq data identified an interaction with the lncRNA, NORAD (*No*n-Coding RNA *A*ctivated by *D*NA Damage). NORAD is an abundant, conserved cytoplasmic lncRNA induced by DNA damage and with key roles in mitotic maintenance, DDR and chromosomal integrity (earning it the name ‘Defender of the genome’ [28]), at least partially through repressing Pumilio-1 and -2 (PUM1/2) proteins, whose activity increases turnover of DDR factors [29, 30]. NORAD has been additionally shown to form a genome-protective ribonucleoprotein complex with RBMX and TOP1 [31]. We show, for the first time, that miR-346 induces extensive DNA damage through chromatin-associations, and demonstrate that the miR-346:NORAD interaction is a clinically-relevant determinant of DNA damage responses, identifying a novel role for NORAD in promoting target-directed miR-346 decay to protect the genome.

## Methods

### Cells lines

In this study, experiments were performed in AR-positive cell lines predominantly modelling the castration-resistant, AR-responsive PC setting, which accounts for the majority of advanced mPC patients, and for whom we believe potential miR-346 based therapeutics would show greatest efficacy due to their accumulation of DDR defects and genomic aberrations. C42, C42B, LNCaP, DU145, 22RV1, BPH1, PNT1a were cultured in RPMI-1640 (Sigma-Aldrich, UK). HEK293 and HEK293T were maintained in DMEM (Sigma-Aldrich, UK). All were supplemented with 10% FCS (First Link, UK) and 2 mM L-glutamine (ThermoFisher, UK) and maintained at 37 °C, 5% CO_2_. Cell lines were purchased from ATCC and authenticated by MWG Eurofins Human Cell Line Authentication, tested monthly for mycoplasma contamination. C42 monoclonal cell lines stably-expressing GFP-tagged pre-miR-346 or shNORAD and RFP-tagged NC-miR under the control of a tetracycline responsive promoter were generated using the shMIMIC Inducible Lentiviral microRNA system (Horizon Biosciences). Doxycycline (0-250 ng/ml) was used to induce transgene expression.

### Plasmids

pcDNA3.1-NORAD was a kind gift from Professor Igor Ulitsky (Weizmann Institute of Science, Israel). pcDNA3.1-NORAD TDMD mutant was generated by iterative site-directed mutagenesis of two putative miR-346 TDMD sites (2367–2390 and 4103–4128) using primers detailed in Supplementary Materials. PUM2 expression plasmid (pFRT/FLAG/HA-DEST PUM2) was obtained from Addgene.

### Western blot analysis

Whole cell lysates were prepared in RIPA buffer supplemented with protease and phosphatase inhibitors. Proteins were resolved on 10–15% SDS-PAGE gels and electroblotted onto PVDF membrane. Membranes were blocked with 5% BSA or dried skimmed milk in TBS-T and incubated in primary antibodies in blocking buffer: anti-pRPA32-Ser^33^ (A300-246A Bethyl Laboratories), anti-phospho-γH2AX-Ser^139^ (Merck Millipore, 05–636), anti-pCHK1-Ser^345^ (Cell signalling #2348), anti-RNA polymerase II CTD repeat YSPTSPS (phospho-S5) (Abcam ab5401), anti-PUM2 (Bethyl Laboratories A300-202A), anti-RNASEH1 (SantaCruz Biotechnologies, H-4, sc-376,326), anti-β-actin (Abcam ab6276), anti-Vinculin (Merck Millipore, V9131), anti-GAPDH (Cell Signalling 14C10 #2118). Membranes were washed and incubated with HRP-conjugated secondary antibodies and blots developed with Luminata™ forte (Merck-Millipore) and imaged using iBright (Invitrogen).

### Immunofluorescence

Cells were fixed with 4% PFA, permeabilized with 0.5% Triton X-100, blocked with 2% BSA and 10% goat serum in TBS and incubated with primary antibodies in blocking buffer: anti-phospho-γH2AX-Ser^139^ (Merck Millipore, 05–636), anti-53BP1 (Novus Biologicals, NB100–904), anti-DNA:RNA hybrid [S9.6] (ENH002, Kerafast). Slides were TBS-washed, incubated in secondary antibodies in blocking buffer (anti-mouse-AlexaFluor-488, anti-mouse-AlexaFluor-594, anti-rabbit-AlexaFluor-488, anti-rabbit-AlexaFluor-594, ThermoFisher) and counterstained with TO-PRO-3 (ThermoFisher, T3605) and DAPI (ThermoFisher, 62,248). Slides were mounted in Vectashield Hardset Antifade Mounting medium (Vector laboratories). Images were acquired using LSM510 confocal microscope (Zeiss) at × 60 magnification. DNA damage foci were quantified using ImageJ.

### Immunohistochemistry

Formalin fixed xenograft tissues were processed into paraffin wax and sections were deparaffinized, hydrated and stained with Hematoxylin&Eosin. Full details of immunohistochemistry are given in Supplementary Methods.

### DNA fibre assay

DNA fibres were analysed using the technique modified from Halliwell et al [32] Full details are given in Supplementary Methods.

### Sulphorhodamine B (SRB) cell growth assay

Cell number was assayed using the SRB assay as previously described [27].

### Caspase assay

Luminescent caspase 3/7 activation assays were performed using the Caspase-Glo 3/7 kit (Promega) according to the manufacturer’s instructions, 48 h post-transfection with miR mimics. Luminescent signal was detected using a Victor luminometer. Caspase activity was normalized to cell number (SRB assay).

### Cell cycle analysis

Cells were trypsin-dissociated, washed with PBS, ethanol-fixed and stored at 20 °C for ≥24 h. Cells were PBS-washed and resuspended in Muse™ cell cycle reagent (Luminex, MCH100106) and incubated for 30 min at RT protected from light. Analysis was performed using the Guava Muse™ Cell Analyzer (Luminex) as specified by manufacturer.

### Alpha-amanitin transcriptional inhibition

Cells were treated with 10 μM Amanitin or vehicle concurrently with miR mimic transfection for 8 h.

### miRNA, siRNA and plasmid transfection

MiRCURY LNA microRNA inhibitors and mimics (Qiagen) and siRNAs (Flexitube, Qiagen) were transfected into cells at final concentration of 0–20 nM in antibiotic-free conditions using Lipofectamine RNAiMax as per the manufacturer’s recommendations. Co-transfection of plasmids and miR mimics was performed using JetPrime transfection reagent (Polyplus) according to the manufacturer’s protocol. Times stated in text and figures refer to the length of time after adding the miR: transfection reagent complexes to the cells.

### Quantitative real-time polymerase chain reaction (qRT-PCR)

Total RNA was extracted using Monarch® total RNA miniprep kit (NEB), reverse transcribed using Precision nanoscript2 reverse transcription kit (PrimerDesign). qRT-PCR was performed using Fast SYBR® Green Master Mix (Applied Biosystems). For miRs, reverse transcription was performed using miRCURY LNA microRNA RT kit (Qiagen) and qPCR performed using miRCURY LNA SYBR® Green PCR kit (Qiagen). QuantStudio™ 7 Flex Real-Time PCR system (ThermoFisher Scientific) was used for quantification. Primer sequences are detailed in Supplementary Information. Expression levels of genes were calculated using the ΔΔC_t_ method and normalised to L19/ GAPDH and SNORD48A/ U6/ 5S rRNA for mRNA and miRs, respectively. For absolute quantification, ten-fold dilutions of known concentrations of miR-346 RNA oligonucleotides or NORAD qPCR amplicon were reverse-transcribed and a standard curve generated.

### Subcellular fractionation

Subcellular fractionation was performed as described [33], and RNA isolated from resultant fractions using Trizol LS (ThermoFisher Scientific, UK) according to the manufacturer’s instructions.

### Endogenous PUM2 RNA Immunoprecipitation

Full details are given in Supplementary Methods. Briefly, prior-transfected cell lysates were incubated with anti-PUM2-conjugated Protein G Dynabeads, PBS-T-washed and RNA extracted using Trizol (ThermoFisher) for reverse transcription and qRT-PCR.

### Biotinylated NORAD RNA Immunoprecipitation

Templates for T7 in vitro transcription of NORAD regions (1950–2110 WT, PRE mutant, miR-346 binding site mutant and negative control region lacking PRE or miR-346 binding sites) were generated by PCR (Phusion High-Fidelity PCR Kit, ThermoFisher), using primers containing T7 RNA Polymerase promoter within forward primer. Single-stranded DNA oligonucleotides corresponding to the above NORAD regions were used as template (see Supplemental Information). T7 In vitro transcription using above-generated templates was performed using Thermo Scientific™ TranscriptAid™ T7 High Yield Transcription Kit (ThermoFisher Scientific) and biotin-16-UTP (Lucigen, USA) to generate biotin-labelled NORAD transcripts. 20 pg biotin-labelled NORAD RNA was incubated with 1 mg cell lysate for 1 h at room temperature with rotation. Biotin-NORAD:protein complexes were isolated using streptavidin-conjugated magnetic beads (88,816, ThermoFisher) at 4 °C for 16 h with rotation, followed by washing (3 × 5 min in TBS-T). Beads were boiled in 2x SDS loading buffer for 5 min and Western blotting performed for PUM2 as described above.

### INDUCE-seq

Pre-treated cells were dispensed into 96 well plates pre-coated with Poly-D-lysine (Greiner bio-one, 655,940) at a density of 1 × 10^5^ cells/well and crosslinked in 4% methanol-free PFA (10 min, RT), then washed × 2 with PBS. Intracellular adapter annealing to dsDNA breaks and INDUCE-seq break mapping were performed as described [34].

### NORAD RNA in situ hybridisation (RNA-ISH)

RNA in situ hybridization (ISH) detection of NORAD was performed using RNAScope reagents (Advanced Cell Diagnostics, USA) according to manufacturer’s instructions. For full details, see Supplementary Methods.

### AGO2/biotin-miR RNA Immunoprecipitation

Performed as described [27].

### RNA-seq

RNA-seq was performed by Novogene. Full details in Supplementary Methods.

### Xenograft tumour growth

8-week male NSG mice (Charles Rivers Laboratories, UK) were injected subcutaneously with 3 × 10^6^ C42/NC (*n* = 8), C42/shNORAD (*n* = 14) or C42/miR-346 (n = 14) cells suspended 50:50 in Matrigel. Tumour volume was measured every 2–3 days, and mice randomly assigned to doxycycline or vehicle treatment for transgene induction (NC miR, shNORAD, miR-346) at tumour volume = 80-100 mm^3^ (volume = length*width*height*π/6). Doxycycline (250 ng/ml) was administered in drinking water containing 1 mg/ml sucrose (vehicle), changed daily. Tumours were allowed to grow to 12.5 mm mean diameter, at which point animals were killed, tumours resected and half flash-frozen, half fixed in neutral-buffered formalin. Statistical significance was calculated using the Mann–Whitney *U* analysis. Animals received food and water ad libitum and were monitored for ill effects. All work was carried out in accordance with the provisions of the Animals (Scientific Procedures) Act 1986 of the United Kingdom (HMSO, London, UK, 1990) and with appropriate local ethical and Health and Safety approval.

### Bioinformatics and statistical analyses

Normally distributed continuous variables were assessed by Student’s t-test. *P* ≤ 0.05 was interpreted to denote statistical significance. Results are presented as mean ± SE or ± SD for at least three independent experiments unless otherwise stated. Pearson correlation coefficient was calculated assuming linear relationship between variables. Gene ontology terms were downloaded from http://geneontology.org/.

TCGA data (processed, Log2 transformed) was accessed from https://tcga-data.nci.nih.gov/docs/publications/prad_2015/ [35]. Additional gene expression data sets were accessed from GEO (https://www.ncbi.nlm.nih.gov/geo/). CRPC (*n* = 159) transcriptome data from SU2C-PCF [36] was re-analysed: paired-end transcriptome sequencing reads were aligned to the human reference genome (GRCh37/hg19) using a RNAseq spliced read mapper Tophat2 [37] (Tophat2.0.7), with default settings. Gene expression, fragments per kilobase of transcript per million mapped reads (FPKM), was calculated using Cufflinks [38].

NORAD Activity Score (NAS) is defined in Supplementary Methods.

For correlation of DDR with NORAD expression and NAS in high-risk PC patients, TCGA-PRAD [35] samples were filtered for high-risk patients [39]. Patients without a diagnostic T-stage or deemed not amenable to surgery (T3b-T4) were removed. Gene expression data was limited to the GRCh37.p13 assembly where gene symbols were mapped against ENSEMBL identifiers with biomaRt [40]. Expression values were log2 transformed with the addition of a pseudo-count (+ 1). Genes were normalised to a mean of zero and unit variance (z-score). DDR and NAS scores were estimated as the average of normalised expression values of signature genes. Each patient receives one score per signature. Pearson correlation was calculated between signature scores and normalised gene expression (cor.test, R programming language). Multiple hypothesis correction followed the false discovery rate methodology [41].

For correlation of NORAD with survival in the Walker et al. cohort of 322 PC prostatectomy samples [42], samples were dichotomised according to NORAD gene expression, comparing the quartile with the highest expression (NORAD-high) to the lowest quartile (NORAD-low). Early-stage PC (T1a-3c Nx M0) was included in analysis. Biochemical recurrence was defined as a rise in PSA of > 0.2 ng/ml and metastatic recurrence as radiological evidence of metastatic disease in lymph node, bone or viscera.

For correlation of NAS with patient outcome, Human Exon 1.0 ST microarray (Thermo-Fisher, Carlsbad, CA) data generated from formalin-fixed paraffin-embedded samples in six published retrospective radical prostatectomy cohorts (*n* = 1567) were accessed [43–48] (accession numbers GSE46691, GSE62116, GSE72291, GSE62667, GSE79956, GSE79957, and GSE7991, respectively) from the GRID database (Decipher Biosciences, San Diego, CA). Microarray processing was performed as previously described [46]. Patients were quartiled according to NAS and measures of survival compared.

INDUCE-seq raw data was processed as described [34]. Full details of analysis are given in Supplemental Methods. Overlap of DSB coordinates between different samples was calculated using GenomicRanges [49]; for miR-346 and shNORAD DSB overlaps, the miR-346 DSBs were extended from single points to 8 nt windows or 100 nt windows centered on the DSBs.

For analysis of relative enrichment of miR-346-induced DSBs at ARE-containing enhancers and promoters in PC cells, these genomic features were defined as described [50]: briefly, promoters are defined as ±2 kb windows of the TSS of all expressed genes in C42 cells (from RNA-seq), enhancers are defined as top 25,000 H3K27ac ChIP-seq peaks located greater than 2 kb from a known TSS of all genes identified in GENCODE, and insulators are defined as the top 50,000 CTCF ChIP-seq peaks that do not overlap with promoters or enhancers. The JASPAR MA0007.2 ARE profile was used (15 bp motif) to find overlaps with AR binding sites. An ARE-positive promoter/enhancer was defined as one containing ≥1 ARE. To find miR-346-induced DSBs breaks in promoter and enhancer regions (+/− AREs), these regions were overlapped with the AREs to create four sets of ranges. These were then overlapped with DSBs from the miR-346 INDUCE-seq dataset, which was summed and normalized by total range length. The number of miR-346-induced DSBs at these sites are presented per kb.

## Results

### miR-346 induces potent DNA damage and replication stress in prostate Cancer cells

**Fig. 1:**
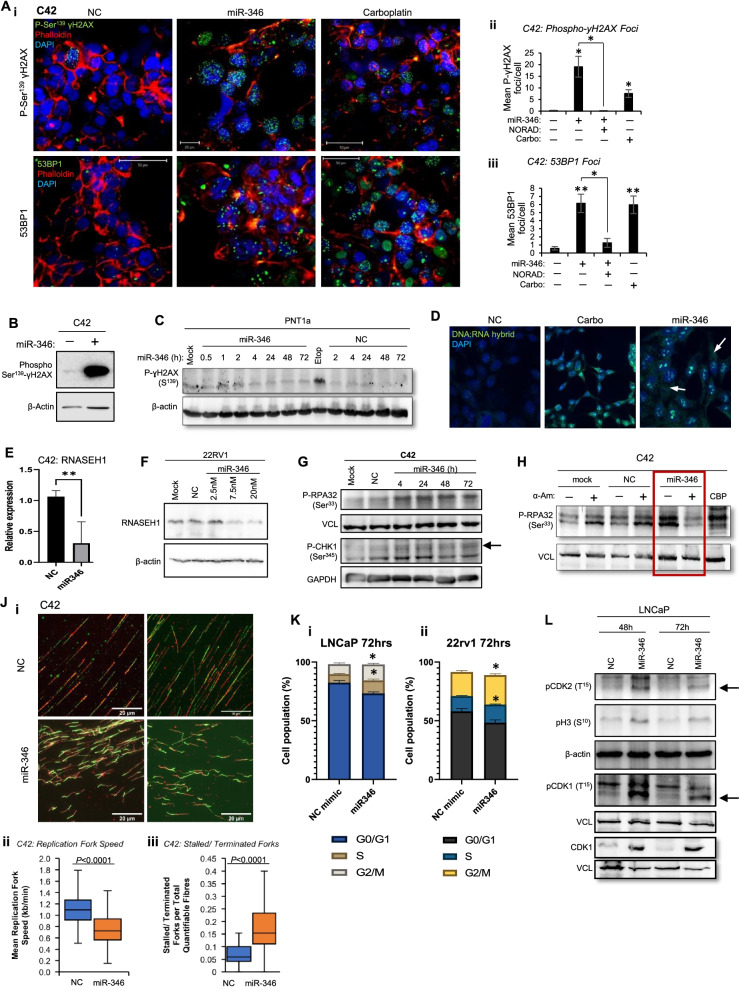
miR-346 Induces Potent DNA Damage and Transcription-Dependent Replication Stress in Prostate Cancer. **A** Immunofluorescent microscopy analysis of i,iii) phospho-Ser^139^-γH2AX and ii,iv) 53BP1 protein levels in C42 cells treated with Carboplatin (2.5 μM) or transfected with 20 nM miR-346 for 72 h. Representative images of three independent experiments are shown. Foci were quantified using ImageJ. **B**, **C** Western blot analysis of phospho-Ser^139^-γH2AX protein levels in B) C42 cells and **C**) PNT1A non-cancerous prostate cells transfected with miR-346 (20 nM) for 96 h (**B**) or indicated durations (**C**). **D** Immunofluorescent microscopy analysis of DNA:RNA hybrids (R-loops) in C42 cells treated with Carboplatin (2.5 μM) or transfected with 20 nM miR-346 for 72 h. Representative images of three independent experiments are shown. **E** RNA-seq analysis of RNASEH1 transcript levels in C42/miR-346 cells treated ± Dox (100 ng/ml). **F** Western blot analysis of RNASEH1 protein levels in 22RV1 cells treated with 10 nM NC mimic or 2.5, 7.5 or 20 nM miR-346 for 72 h. **G**, **H** Western blot analysis of: G) phospho-Ser^33^-RPA32 and phospho-Ser^345^-CHK1 protein levels in C42 cells transfected with miR-346 (20 nM) for the indicated durations, H) phospho-Ser^33^-RPA32 protein levels in C42 cells transfected with 10 nM miR-346 and treated with 10 μM α-Am for 8 h. **J** DNA fibre assay analysis of replication fork speed and stalled/terminated forks (per quantifiable fibre) in C42 cells transfected with miR-346 or negative control miR (20 nM) for 24 h. A minimum of 100 fibres were quantified for each measurement and different replication events quantified in ImageJ as described [32]. Fields were selected using one fluorescence channel only for the avoidance of bias. Scale bars = 20 μm. **K** Flow cytometric analysis of cell cycle distributions of LNCaP (i) and 22RV1 (ii) cells transfected with NC or miR-346 mimic (10 nM) for 72 h. **L** Western blot analysis of phospho-Thr^15^-CDK2, phospho-Ser^10^-Histone H3, phospho-Thr^15^-CDK1 and total CDK1 in LNCaP cells transfected with10nM miR-346 or NC miR for 48 and 72 h. **B**, **C**, **F**, **G**, **H**, **L** Representative images of three independent experiments are shown, β-actin, GAPDH and VCL were used as a loading controls. CBP = carboplatin, α-Am = alpha-amanitin. Etoposide and CBP were used as positive controls for DNA damage induction. Columns: mean ± SEM for a minimum of three independent experiments. * *P* ≤ 0.05, # *P* ≤ 0.005, α *P* ≤ 0.0001. See also Fig. S1–4

In a previous AGO-PAR-CLIP-seq analysis [51], miR-346-bound transcripts were identified in a number of PC cell lines. Pathway analysis of these revealed enrichment for cell cycle, DNA replication, DNA repair and chromatin organisation (Fig. S1A). Since miR-346 modulates AR activity [27], and AR causes transcription-associated DNA damage [4, 5, 52–54], we hypothesised that miR-346 modulates DDR in PC. Interestingly, immunofluorescent staining of phospho-γH2AX and 53BP1 foci in C42 cells demonstrated potent DNA damage induction upon miR-346 treatment (Fig. 1A), supported by phospho-γH2AX Western blotting (Fig. 1B, S1B). Dose-responsiveness of miR-346-induced DNA damage was confirmed in C42, LNCaP and 22RV1 cells (Fig. S1C-E). Doxycycline-treated C42 monoclones stably expressing GFP-tagged pre-miR-346 under control of a tetracycline-responsive promoter similarly revealed increased phospho-γH2AX protein levels (Fig. S1F) compared to negative control-expressing cells, confirming that miR-346 induction of DNA damage is not specific to one mode of miR overexpression. Importantly, miR-346 did not significantly increase phospho-γH2AX protein levels in non-malignant (PNT1a) or benign (BPH1) prostate epithelial cells (Fig. 1C, S1G,H).

miR-346 was shown to induce R-loop formation (these impede replication fork processivity resulting in replication stress), as assayed using a DNA:RNA hybrid-detecting antibody (Fig. 1D), suggesting that miR-346 induces DNA damage within transcriptionally-active loci. Interestingly, miR-346 results in downregulation of *RNASEH1*, both at the transcript and protein levels (Fig. 1E,F, Fig. S1J)*,* which is responsible for R-loop resolution; this positive feedback mechanism may perpetuate DNA damage. Cytosolic dsDNA:RNA hybrids were observed in the cytoplasm of both carboplatin and miR-346-treated cells (Fig. 1D, white arrows), consistent with observations of cytosolic R-loops following DNA damage [55].

Protein levels of two further replication stress markers, Ser^33^-phosphorylated replication protein A2 (RPA2/RPA32) and Ser^345^-phosphorylated CHK1, were increased following 4 h of miR-346 treatment in C42 cells (Fig. 1G, Fig. S2A,B); phospho-RPA32 was also increased following doxycycline induction of pre-miR-46 in C42/miR-346 cells (Fig. S2C). In addition, many DNA polymerases, helicases and ligases required for DNA replication are significantly repressed following miR-346 overexpression (Fig. S2D), perhaps facilitating DNA replication pausing in response to widespread miR-346-induced R-loops, since none of these transcripts are identified as direct miR-346 targets in PC [51].

Given that miR-346 increased R-loops, which form during active transcription, we hypothesised that miR-346-induced DNA damage may require ongoing transcription. This was confirmed when treatment of C42 cells with the transcriptional inhibitor α-Amanitin after transfection with miR-346 entirely abrogated induction of DNA damage (phospho-RPA32(Ser^33^) (Fig. 1H, S2E).

To further investigate the impact of miR-346 on DNA replication dynamics, DNA fibre assays were performed. miR-346 significantly decreased replication fork speed and increased the numbers of stalled or terminated fibres [32, 56] (Fig. 1J, Fig. S3 - hydroxyurea, a positive control, resulted in very short red-only fibres indicative of severely slowed/stalled replication forks). In flow cytometric analysis, we found that miR-346 significantly enhanced accumulation of LNCaP cells both in S and G2/M phases (Fig. 1K, S4A), indicative of cell cycle arrest at these stages. Commensurate with this, levels of phospho-CDK2(T^15^) (G1/S arrest marker), phospho-Histone H3(S^10^) and total- and phospho-CDK1 (M-phase arrest markers) were increased at the same time-points (Fig. 1L, Fig. S4B-E).

Together, these data indicate that miR-346 induces R-loop formation and DNA replication fork stalling, resulting in DNA damage and cell cycle arrest.

### miR-346 and the LncRNA NORAD both associate with clinical outcomes and their direct interaction limits miR-346-induced DNA damage

**Fig. 2:**
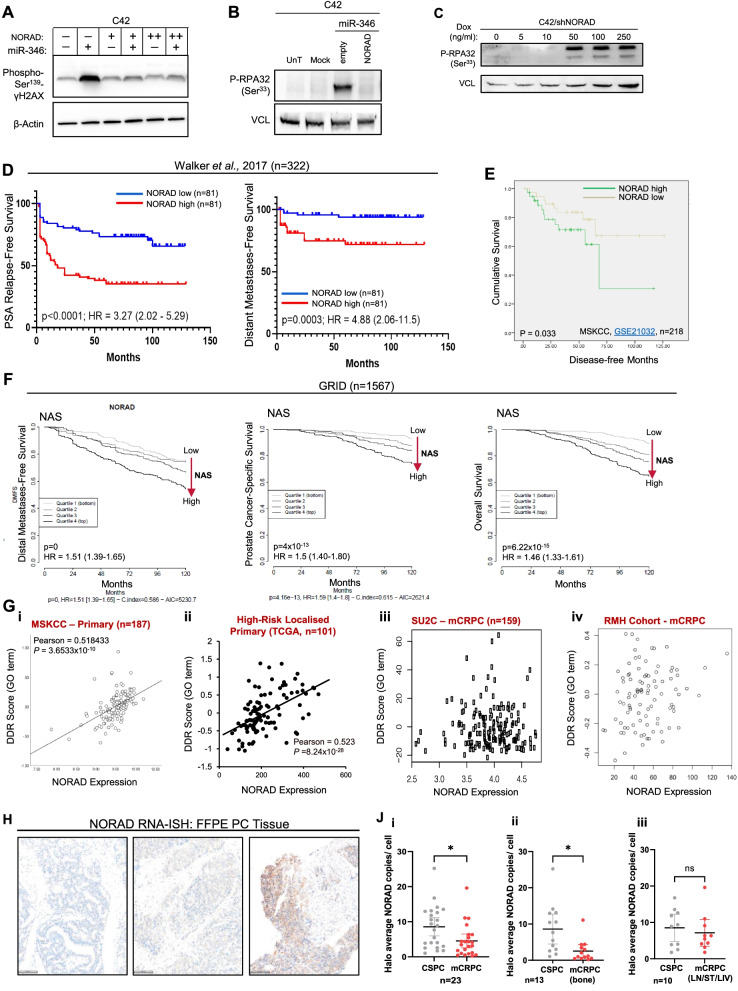
LncRNA, NORAD, Rescues miR-346-Induced DNA Damage and is Associated with Adverse Prostate Cancer Outcome. **A** Western blot analysis of phospho-Ser^139^-γH2AX protein levels in C42 cells transfected with miR-346 (10 nM) ± pcDNA3.1-NORAD or empty plasmid for 96 h. B,C) Western blot analysis of phospho-RPA32(Ser33) protein levels in **B**) C42 cells transfected with miR-346 (10 nM) ± pcDNA3.1-NORAD or empty plasmid for 72 h and **C**) C42/shNORAD monoclone #2–12 treated with indicated doxycycline concentrations for 72 h. **A**-**C** Representative images of three independent experiments are shown, β-actin and VCL were used as a loading controls. **D**, **E** PSA relapse-free survival and distant metastasis-free survival (**D**) and overall survival (**E**) of PC patients dichotomised into NORAD low and high groups in the Walker et al. radical prostatectomy cohort (*n* = 322) [35] (**D**) and MSKCC cohort [36] (GSE21032, *n* = 218) (**E**). **F** Distal metastasis-free survival, PC-specific survival and overall survival in patients from six retrospective radical prostatectomy cohorts (*n* = 1567) with long-term clinical outcomes [37–42] accessed from the GRID database and quartiled according to NORAD Activity Score (NAS). **G** Correlation of NORAD expression with DNA damage response score in indicated patient tumour gene expression data sets [26–33]. **H**, **J** NORAD RNA in situ hybridisation analysis of NORAD RNA copies in matched castration-sensitive (CSPC) and metastatic castration-resistant PC (mCRPC), *n* = 23, LN = lymph node, LIV = liver, ST = other soft tissue. Representative images of primary tumours are shown (A - low, medium and high expression, scale bar = 100 μm, brown = NORAD RNA). * *P* ≤ 0.05. See also Fig. S5–7, Table S1

To determine the mechanism(s) by which miR-346 induces DNA damage, we identified miR-346 targets through the interrogation of a PC AGO-PAR-CLIP-seq database of miR:RNA interactions [51]. One of the top miR-346 interacting transcripts is the lncRNA NORAD (Table S1), implicated in maintenance of mitosis, DNA repair and chromosomal integrity [29, 30]. miR-346:NORAD interaction in PC cells was confirmed (Fig. S5A). Importantly, ectopic expression of full-length NORAD rescued miR-346-induced DNA damage in both C42 and 22RV1 cells (phospho-γH2AX/phospho-RPA32 immunoblotting: Fig. 2A,B, Fig. S5B-D) and NORAD silencing (siRNA or dox-induced shRNA) increased protein levels of phospho-RPA32 (Fig. 2C, Fig. S5E,F). This supports a regulatory relationship between NORAD and miR-346 in modulating PC DDR.

To determine the relevance of NORAD in human PC, we next correlated NORAD expression or activity (NORAD activity score, NAS) with patient outcome across multiple PC patient cohorts. High NORAD levels significantly correlated with poor outcome (PSA relapse-free survival and distant metastasis-free survival) in the Walker et al. [42] cohort of 322 radical prostatectomy samples with long-term follow-up, and in the MSKCC cohort [57] of 218 prostate tumours (181 primary from prostatectomy, 37 metastases, GSE21032) (Fig. 2D,E). Patients with high NORAD levels show reduced overall survival compared to those with low NORAD levels in TCGA-PRAD (lack of significance may be in part due to shorter patient follow-up in this cohort (200 days vs 125 months [MKSCC] and 150 months [Walker et al. [42]]). We observed no correlation between NORAD expression and patient survival in either of the mCRPC cohorts examined (SU2C and Royal Marsden Hospital [RMH] - Fig. S6B,C). We believe that this is because the influence of NORAD on survival is eclipsed by the influence of gross aberrations in key DDR pathways at this advanced disease stage.

High NAS also associated with reduced overall survival, PC-specific survival, *PSA* relapse-free survival and distant metastasis-free survival in microarray data from six retrospective radical prostatectomy sample datasets (primary PC, total *n* = 1567) with long-term clinical outcomes [43–48] (Fig. 2F). In contrast, miR-346 was associated with improved survival (*p =* 0.087, Fig. S6D) but was decreased with increasing Gleason grade (Fig. S6E). This supports potential antagonistic roles for NORAD and miR-346 in PC.

Importantly, both NORAD expression and NAS strongly correlated with DDR in localised and high-risk localised PC, but surprisingly not in mCRPC (Fig. 2G, Fig. S7A). This may support a partial uncoupling of NORAD from DNA repair processes in advanced PC, where extensive genomic aberrations may override NORAD activity. This is in keeping with the lack of correlation between NORAD expression and patient survival in mCRPC (Fig. S6B,C). However, *NORAD* expression and NAS remained strongly positively correlated with known DNA repair- and genome integrity-related factors, such as *TOP1*, *BRCA2* and *CDK12* expression, UV response via ERCC3-mediated NHEJ, and SMARCA2-directed chromatin remodelling, in the same mCRPC dataset (Fig. S7B-J). Alternatively, the lack of correlation of NORAD with survival and DDR in mCRPC may be attributable to loss of NORAD expression during PC progression; interestingly, NORAD RNA-ISH in FFPE tumour sections from castration-sensitive localised PC (CSPC) and matched, same patient, metastatic castration-resistant PC (mCRPC), *n* = 23, revealed that NORAD RNA copies are significantly lower in mCRPC as compared to CSPC (Fig. 2H,J). This difference was particularly obvious when bone metastases (most common) were considered separately (Fig. 2Jii), but was lost when considering visceral metastases (Fig. 2Jiii). NORAD levels were also consistently higher in cancer stroma than in PC regions, regardless of site (Fig. S7K).

### miR-346 modulates NORAD genome-protective activity in prostate Cancer

**Fig. 3:**
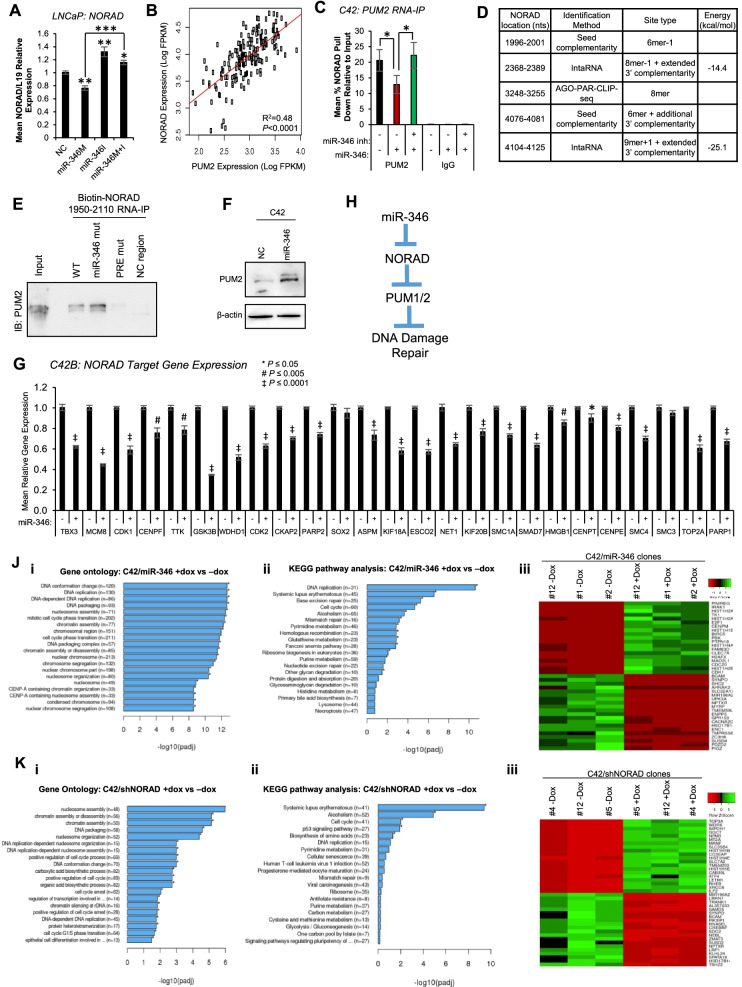
miR-346 Associates with NORAD to Inhibit its Genome Integrity-, DNA Replication- and DNA Repair-Promoting Activity in Prostate Cancer. **A** qRT-PCR analysis of NORAD expression in LNCaP cells transfected with miR-346 ± miR-346 inhibitor (both 10 nM) for 72 h. **B** Correlation between NORAD expression and PUM2 expression in SU2C mCRPC patient data set. **C** qRT-PCR analysis of NORAD transcript levels in PUM2 and IgG immunoprecipitates from C42 cells transfected ± miR-346 (10 nM) and miR-346 inhibitor (10 nM) for 24 h. **D** miR-346 binding sites within NORAD identified from PC AGO-PAR-CLIP-seq [51], IntaRNA (http://rna.informatik.uni-freiburg.de/IntaRNA/Input.jsp) and by seed complementarity. **E** Western blot analysis of PUM2 protein levels in biotin-NORAD 1950–2110 (WT or mutant, as indicated) immunoprecipitates from C42 cells. **F** Western blot analysis of PUM2 protein levels in C42 cells transfected with miR-346 or negative control miR (20 nM) for 72 h. β-actin was used as a loading control and a representative image is shown. **G** qRT-PCR analysis of NORAD target gene [29, 30] expression in C42B cells transfected ± miR-346 (20 nM) for 72 h. L19 was used as a normalisation gene. Columns: mean ± SEM for three independent experiments performed in triplicate. H) Proposed mechanism of miR-346 regulation of NORAD:PUM2 DNA damage response axis. **J**, **K** Gene ontology (i) and KEGG (ii) pathway analysis of differentially-expressed genes identified by RNA-seq of C42/miR-346 (**J**) and C42/shNORAD (K) cells (three independent monoclones) ± dox. Top 20 up- and down-regulated transcripts are shown (Jiii, Kiii). miR-346 BS mut = miR-346 binding site-mutant NORAD, PRE mut = Pumilio recognition element-mutant NORAD, NC = negative control NORAD region. * *P* ≤ 0.05, ** *P* ≤ 0.005, *** *P* ≤ 0.0001. See also Fig. S8, S9, S10 and Tables S2–6

We sought to further characterise the relevance of the miR-346:NORAD interaction in PC DDR. To investigate whether miR-346 targets NORAD for canonical AGO2-mediated transcript degradation, qRT-PCR was performed following transfection of LNCaP and 22RV1 cells; this demonstrated a modest yet significant reduction in NORAD levels after miR-346 treatment that was rescued by the addition of miR-346 inhibitor (anti-sense oligonucleotide to miR-346), confirming effect specificity (Fig. 3A, Fig. S8A). NORAD has previously been reported to function through sequestration of PUM2 [29, 30]. To investigate the relevance of PUM2 for NORAD function in PC, we assessed the relationship between NORAD and PUM2 expression in publicly-available RNA-seq datasets: NORAD expression significantly correlated with PUM2 in both localised PC (Fig. S8B) and mCRPC (Fig. 3B), and also with PUM1 (Fig. S8C). Since miR-346 addition only modestly reduced NORAD levels, we hypothesised that to exert its effects, miR-346 may disrupt NORAD:PUM2 interaction. qRT-PCR for NORAD in PUM2 immunoprecipitates from C42 cells showed that miR-346 transfection reduced NORAD:PUM2 interaction by 50%, with this effect being rescued by addition of miR-346 inhibitor (Fig. 3C). We hypothesised that this disruption results from miR-346 binding NORAD near PUM recognition elements (PREs) [30].

AGO-PAR-CLIP identified interaction of miR-346 with NORAD nucleotides 3248–3255, whilst IntaRNA (http://rna.informatik.uni-freiburg.de/IntaRNA/) [58] analysis of RNA:RNA associations revealed two 8/9mer interactions with extended 3′ complementarity at 2368–2389 and 4104–4125, and two further 6mer sites at 1996–2001 and 4076–4081 were identified by analysing NORAD sequence for miR-346 complementarity (Fig. 3D). Prediction of NORAD RNA folding revealed the presence of putative miR-346 binding sites predominantly in stem loop-structured regions (http://rna.tbi.univie.ac.at/cgi-bin/RNAWebSuite/RNAfold.cgi, Fig. S8D). One of these sites, at 1996–2001, is located close to a PRE [30], so we examined whether mutation of this site modulated PUM2 association. Biotin-labelled 160 nt NORAD RNA fragments spanning this region, either wild-type or carrying miR-346 binding site mutation or PRE-mutation, were incubated with C42 PC cell lysates. Mutation of the putative miR-346 binding site significantly increased PUM2 association with NORAD (Fig. 3E, S8E), whilst PRE mutation totally abrogated NORAD immunoprecipitation of PUM2 (Fig. 3E, S8E). Together these data support a model whereby high miR-346 disrupts NORAD sequestration of PUM2, liberating PUM2 protein to turnover DNA repair transcripts (Fig. 3H). Indeed, miR-346 addition significantly increased PUM2 protein (Fig. 3F, S8F), but not transcript levels (Fig. S8G), suggesting that miR-346 regulation of PUM2 is post-transcriptional, and likely due to loss of NORAD sequestration and enhanced stability of liberated PUM2. In support of the functional relevance of miR-346 modulation of NORAD:PUM2 interaction, miR-346 addition significantly decreased expression of 29 of 33 previously-identified NORAD-upregulated transcripts [29, 30] (Fig. 3G).

Together, these data suggest that miR-346 modulation of NORAD:PUM2 interactions alters critical DDR processes in PC (Fig. 3H). High NORAD levels promote efficient DDR, whilst elevated miR-346 inhibits NORAD-mediated DNA repair by disrupting NORAD:PUM2 sequestration.

### miR-346 and NORAD impact DNA replication, DNA damage and cell cycle in advanced PC

To appreciate the transcriptome-wide implications of miR-346 and NORAD activity in advanced PC, we performed RNA-seq analysis of C42/miR-346 and C42/shNORAD (stably expressing miR-346 and shNORAD, respectively, under doxycycline control) cells ±Dox, and also C42 cells transiently transfected with siNORAD or negative control siRNA (siNC). Data analysis was performed as shown (Fig. S9A). Read quality and number of mapped reads are shown and overall gene expression levels were similar between samples (Fig. S9B-D). Of note, both siNORAD and shNORAD increased the percentage of intronicly-mapped reads (Fig. S9E), which may suggest a role for NORAD in splicing and pre-mRNA processing. Numbers of significantly up- and down-regulated genes are shown (Fig. S10A). In line with potent miR-346 induction of DNA damage and replication stress (Fig. 1), 18 of the top 20 miR-346-enriched gene ontology (GO) terms relate to DNA replication, packaging, conformation and chromatin assembly (Fig. 3Ji – similar results for KEGG pathway analysis (Fig. 3Jii), and gene set enrichment analysis (GSEA, Table S2)). Further, GO analysis of shared miR-346 RNA-seq dysregulated genes and AGO-PAR-CLIP-seq-identified miR-346-bound transcripts in PC [51] suggests that canonical miR-346 gene repression modulates DNA replication and DNA damage in mCRPC (Table S3). Importantly, however, only 378 of the 3943 RNA-seq-identified miR-346-regulated transcripts were also found to interact directly with miR-346 in the cytoplasmic RISC complex by PC AGO-PAR-CLIP-seq [51], suggestive of important non-canonical, non-cytoplasmic or indirect miR-346 activity in modulating DNA damage. The top twenty significantly miR-346-upregulated and -downregulated genes are shown (Fig. 3Jiii).

In line with NORAD promotion of genome integrity and DNA replication fidelity [29, 30], 15 of 20 shNORAD-enriched terms relate to chromatin assembly, DNA packaging, DNA replication and cell cycle (Fig. 3Ki). Similar results were obtained from KEGG pathway analysis (Fig. 3Kii) and GSEA (Table S4). Importantly, more than 2000 genes were dysregulated by both shNORAD- and siNORAD (Fig. S10B). To identify key pathways modulated by miR-346:NORAD signalling, while negating effects of mode of NORAD silencing, we examined genes commonly dysregulated by all of shNORAD, siNORAD and miR-346, identifying 583 genes differentially-regulated under all conditions (Fig. S10C). Pathway analysis of shared genes demonstrates convergence upon DNA recombination, isotype switching, DNA replication, chromatin remodelling, translation, cholesterol biosynthesis and cell cycle (Table S5). Of note, miR-346-downregulated transcripts share considerable overlap with siPUM2-downregulated genes (Fig. S10D), and shared miR-346 and siPUM2 DEGs converge upon DNA replication, DNA repair and cell cycle processes (Table S6), suggesting that at least some of the canonical miR-346 modulation of DNA damage may be mediated via PUM2. qRT-PCR analysis of a subset of miR-346 and shNORAD/siNORAD DEGss validated RNA-seq findings (Fig. S10E,F).

### miR-346 induces genome-wide dsDNA breaks at transcription start sites

**Fig. 4:**
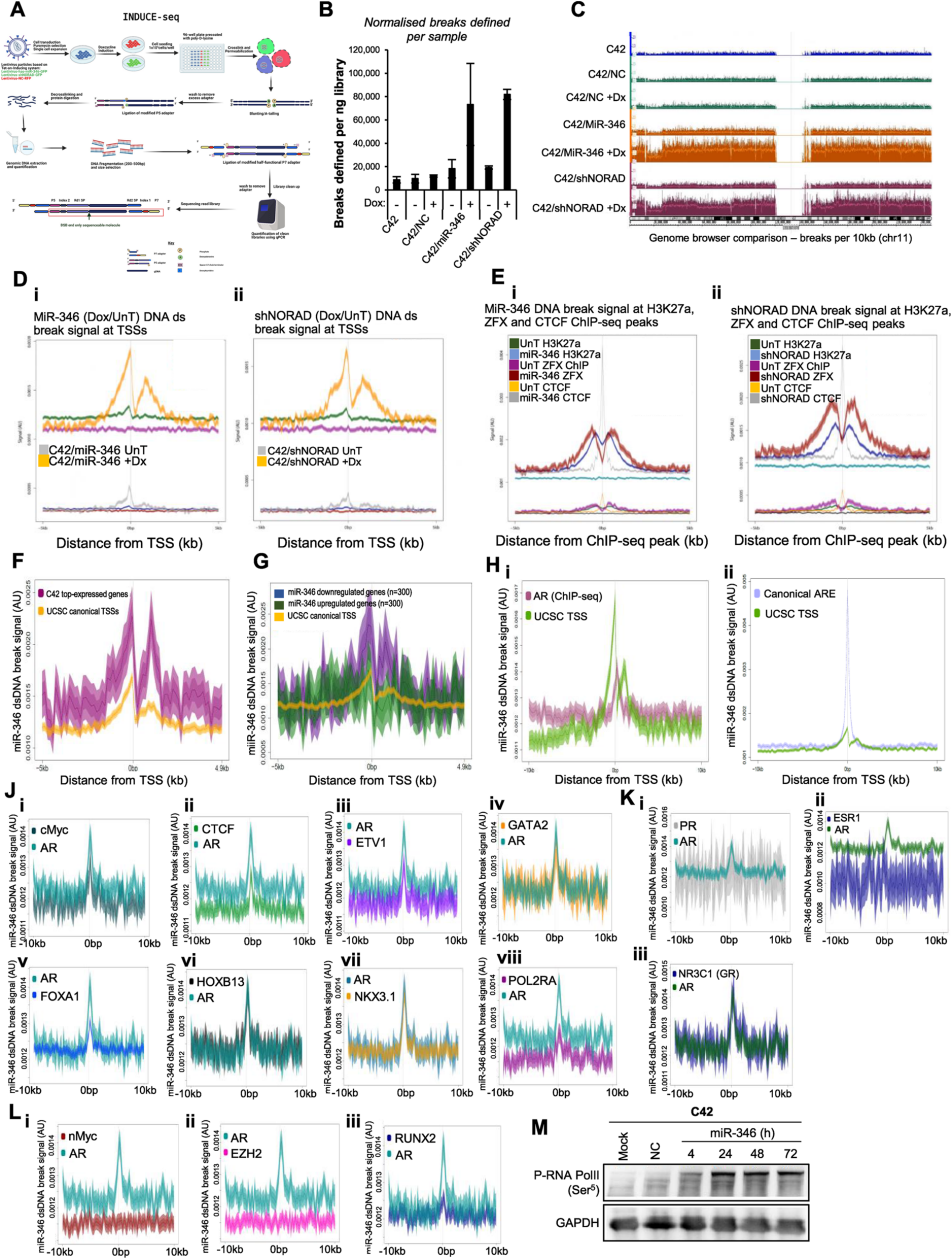
miR-346 Induces Genome-Wide Double Strand DNA Breaks at Transcription Start Sites. **A** Schematic illustration of INDUCE-seq protocol, **B** Quantification of number of ds DNA breaks identified per ng of sequencing library, **C** Genome browser comparison of number of breaks per kb across chromosome 11 for C42, C42/NC, C42/miR-346 and C42/shNORAD cells treated ± Dox. Peak height corresponds to number of DNA breaks. Similar results were obtained for other chromosomes. **D**, **E** Integration of miR-346 and shNORAD dsDNA break coordinates with **D**) annotated transcription start sites (TSSs - hg19 refseq gene list) and **E**) PC ChIP-seq-identified CTCF, ZFX and histone H3K27ac sites. **F**) top 1000 genes expressed in C42 cells show greater enrichment of miR-346-induced dsDNA breaks at their TSSs (dark red) compared to total C42-expressed genes (yellow), **G** enrichment of miR-346 dsDNA breaks at TSSs of miR-346-downregulated (dark blue), but not –upregulated (dark green), genes. **H** Enrichment of miR-346 DSBs at (i) ChIP-seq-identified AR binding sites (LNCaP cells - pink) and (ii) canonical AREs (consensus ARE sequence from JASPAR [http://jaspar.genereg.net/]- blue). miR-346 DSB enrichment at TSSs from hg19 refseq gene list is shown for reference. **J**, **K**, **L** Integration of miR-346 dsDNA break coordinates with PC ChIP-seq-identified binding sites for (J) PC-implicated TFs/ pioneer factors cMyc (GSM1907204), CTCF (GSM2827203), ETV1 (GSM1145322), GATA2 (GSM941195), FOXA1 (GSM1863005), HOXB13 (GSM2480818), NKX3.1 (GSM989640) and POL2RA (GSM696843) (**K**) AR-related Nuclear Receptor family members PR (GSM50151), ESR1 (GSE43988) and NR3C1 (GR) (GSM759669) and (**L**) neuroendocrine PC-associated TFs nMyc (GSM2305251), EZH2 (GSM2305255) and RUNX2 (GSM838400). **M** Western blot analysis of phospho-Ser5-RNA PolII protein levels in C42 cells transfected with miR-346 or NC (20 nM) for indicated durations. GAPDH was used as a control for loading and a representative image is shown. See also Fig. S11

To further characterise mechanisms by which miR-346 causes DNA damage, we used INDUCE-seq for in situ amplification-free, single nucleotide-resolution mapping of dsDNA breaks [34] (Fig. 4A) in C42/NC, C42/miR-346 and C42/shNORAD cells ±Dox. Both miR-346 and shNORAD were shown to dramatically increase DNA breaks pan-genome, generating 3,316,725 and 2,869,894 dsDNA breaks, respectively, compared to 381,972 breaks in C42/NC + dox, and 230,364 in untreated C42 cells (Fig. 4B,C). Notably, only 2.7% of miR-346-induced DSBs overlap with shNORAD-induced DSBs (±4 nt), rising to 13.5% if an overlap window of ±50 nt is used (Fig. S11A). This suggests that whilst there is some overlap between miR-346- and shNORAD-induced DSBs, they induce breaks predominantly at different genomic positions, potentially by different mechanisms. This is supportive of NORAD-independent functions of miR-346. However, the downstream impacts of both miR-346 and shNORAD converge upon the same major processes of DNA replication, DNA repair and cell cycle (Fig. 3).

Of 18,958 genes present in hg19, 17,222 (90.8%) contained at least one miR-346-induced break. miR-346 and shNORAD breaks were enriched at transcription start sites (TSSs), since DSBs were identified at a frequency of 81 and 69, respectively, per 100,000 bp within TSS regions (UCSC TSSs ±200 bp), respectively, compared to 62 and 53 per 100,000 bp within annotated gene regions. Importantly, only 49% of miR-346 and shNORAD DSBs localised to actively transcribed regions. The implications of breaks in non-transcribed regions is subject to ongoing study.

Integration of UCSC-annotated TSSs and transcription factor binding sites from C42 PC cells with INDUCE-seq DSB coordinates revealed strong overlap of miR-346- and shNORAD-induced DNA breaks with TSSs and binding sites for CTCF, ZFX transcription factors and H3K27ac marker of active transcription (Fig. 4D,E). In addition, miR-346-induced breaks were significantly enriched in the top 1000 most highly-expressed genes in C42 cells, consistent with the requirement for active transcription for miR-346-induced breaks (Fig. 4F), and miR-346-induced breaks were enriched at the TSSs of miR-346-downregulated, but not -upregulated genes (Fig. 4G), suggesting that miR-346-induced DSBs result in reduced expression of the most highly-transcribed genes. Notably, shNORAD DSB profiles showed similar overlap with TSSs and TFs, supporting mechanistic convergence of NORAD and miR-346. Since miR-346 DSBs are enriched at TSSs, and AR is one of the most active TFs in PC, we examined enrichment of miR-346 DSBs at canonical Androgen Response Elements (AREs – JASPAR [http://jaspar.genereg.net/]) and ChIP-seq-identified AR-binding sites from LNCaP cells. miR-346 DSBs were found to be significantly enriched at both site types (Fig. 4H).

Since large numbers of AR binding sites occur at enhancer regions, we examined the relative enrichment of miR-346-induced DSBs at previously-defined PC enhancers versus promoters [50] (see Material and Methods for detailed methodology). These analyses revealed that the number of miR-346-induced DSBs is greater at ARE-containing (1.16/kb) versus non-ARE-containing (0.79/kb) promoters, but this was not true at enhancers (0.81/kb versus 0.78/kb) (Fig. S11B). In addition, there are higher numbers of miR-346-induced DSBs per kb at ARE-containing promoters (1.16/kb) versus ARE-containing enhancers (0.81/kb) (Fig. S11B). Both observations are consistent with our model that miR-346 induces DSB by inducing transcriptional hyperactivation at sites of the most active TFs in PC.

Further examination of miR-346 DSB association with ChIP-seq-identified TF binding sites from LNCaP cells revealed enrichment at c-Myc, CTCF, ETV1, GATA2, FOXA1, HOXB13, NKX3.1, and POLR2A binding sites (Fig. 4J). These TFs were selected for analysis due to their recognised key roles in PC development and/or progression. Importantly, there was no enrichment of miR-346 DSBs at binding sites for AR-related nuclear receptor TFs, Estrogen Receptor (ESR1) and Progesterone Receptor (PR) (Fig. 4K). However, miR-346 DSB enrichment was observed at binding sites of the Glucocorticoid Receptor (GR, NR3C1), which is highly active in PC, and shares a very high degree of sequence homology and DNA response element specificity with AR (Fig. 4K). Further, no miR-346 DSB enrichment was observed at binding sites for TFs associated with neuroendocrine PC (NEPC), such as EZH2, n-Myc and RUNX2 (Fig. 4L).

De novo motif enrichment analysis was conducted to identify DNA sequence specificity of miR-346-induced DSBs (Fig. S11C). Notably, limited motif enrichment was identified in miR-346-induced vs endogenous DSBs: although consensus NKX3.1 binding sites were identified within 50 bp of 43.2% of miR-346-induced DSBs, such motifs were also identified in 34.5% of endogenous DSBs in C42 cells (Fig. S11C). These data are consistent with identified enrichment of miR-346 DSBs at ChIP-seq-identified NKX3.1 binding sites (Fig. 4Jvii).

Together these data suggest that miR-346 DSBs occur preferentially at binding sites of the most highly-transcriptionally active TFs in PC cells in a non-DNA sequence-specific manner. In keeping with this, miR-346 was shown to significantly increase levels of Ser^5^-phosphorylated RNA polymerase II, a marker of transcription initiation (Fig. 4M, Fig. S11D,E).

### miR-346 induces rapid DNA damage independently of the NORAD/PUM2 Axis and NORAD drives target-directed microRNA decay of miR-346

**Fig. 5:**
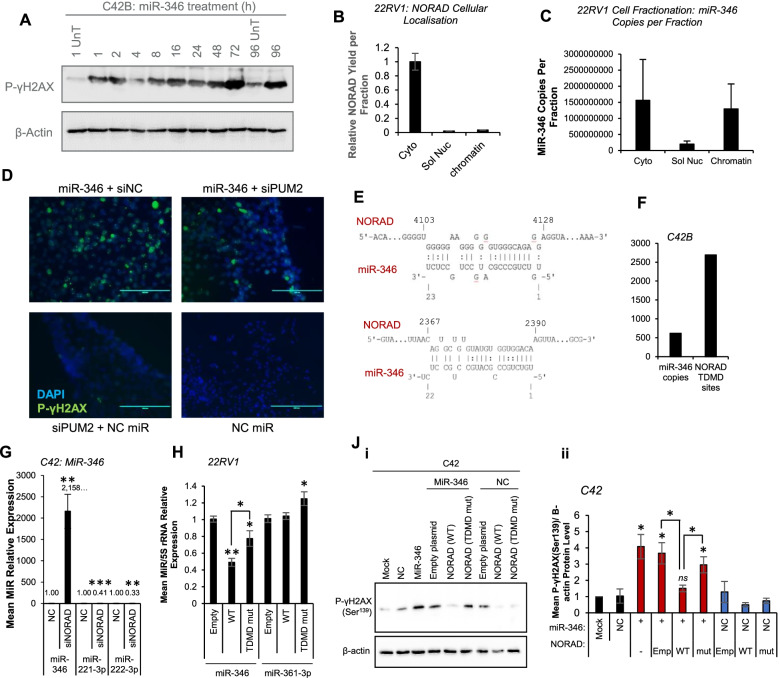
miR-346 Induces DNA Damage in part Independently of NORAD/PUM2, NORAD Promotes Target-Directed miR-346 Decay. **A** Western blot analysis of phospho-Ser^139^-γH2AX protein levels in LNCaP cells transfected with 20 nM miR-346 for indicated duration. β-actin was used as a loading control. **B**, **C** qRT-PCR analysis of B) NORAD and **C**) miR-346 in cytosolic, soluble nuclear and chromatin fractions of 22RV1 cells. SNORD48 was used as a fractionation control and identified almost exclusively complexed with chromatin (Fig. S12B). **D** Immunofluorescent microscopy analysis of i,iii) phospho-Ser^139^-γH2AX protein levels in C42 cells transfected with miR-346 or NC (20 nM) ± siPUM2 (20 nM) for 72 h. **E** Illustration of NORAD:miR-346 putative interaction sites with features consistent with target-directed microRNA decay. **F** qRT-PCR quantification of number of miR-346 copies and NORAD TDMD sites in unperturbed C42B cells. Ten-fold serial dilutions of a miR-346 mimic and NORAD qPCR amplicon were prepared, reverse transcribed and analysed by qRT-PCR in parallel to C42B samples for absolute quantification. **G** qRT-PCR analysis of miR-346, − 221-3p and 222-3p levels in C42 cells transfected with siNORAD or siNC for 72 h. miR levels were normalised to U6, **H** qRT-PCR analysis of miR-346 levels in 22RV1 cells transfected with pcDNA3.1-NORAD WT or TDMD mutant for 72 h. miR levels were normalised to U6. Columns: mean ± SEM for three independent experiments performed in triplicate. **J** Western blot analysis of phospho-Ser^139^-γH2AX protein levels in C42 cells transfected with miR-346 (10 nM) ± WT or TDMD-mutant NORAD. β-actin was used as a loading control. A representative image of four independent experiments is shown. Densitometry was performed using ImageJ. * *P* ≤ 0.05, ** *P* ≤ 0.005, *** *P* ≤ 0.0001. See also Fig. S12, Table S7

To further investigate the dynamics of miR-346-induced DNA damage, we evaluated phospho-γH2AX protein levels across a time-course after miR-346 transfection in PC cells; very rapid DNA damage was observed after as little as 1 h (Fig. 5A, Fig. S12A). Further, fractionation of 22RV1 cells showed that, whilst NORAD is predominantly cytoplasmic (Fig. 5B), consistent with prior reports [30], more than 50% of endogenous miR-346 copies are in the nucleus, and largely chromatin-associated (Fig. 5C). Together with the observation that miR-346 can induce DNA damage following PUM2 silencing (Fig. 5D, Fig. S12C), these results suggest that miR-346 is able to induce DNA damage in part independently of disrupting NORAD sequestration of PUM2.

More than half of the top NORAD associated pathways in mCRPC are miR activity pathways (Table S7), suggesting a potential role for NORAD in miR regulation in advanced PC. Since endogenous miR-346 copies are low and NORAD copies high in PC cells, we considered the possibility that, under physiological conditions, NORAD regulation of miR-346 may serve as a novel genome protection mechanism to guard against miR-346-induced DNA damage. The NORAD:miR-346 interaction has several features consistent with an emerging phenomenon called target-directed microRNA decay (TDMD), whereby miR:transcript binding results in miR, rather than target, turnover: these features include cytosolic lncRNA localisation, extended binding site complementarity and single nucleotide bulges towards miR-346 3′ end to prevent AGO2 slicing of NORAD. Two potential NORAD TDMD sites were identified (Fig. 5E) and the number of potential NORAD TDMD sites per (C42B) cell was found to be 4.2-fold higher than the number of miR-346 copies (Fig. 5F). Notably, putative TDMD sites at 2368–2389 and 4104–4125 are located in regions lacking PREs, suggesting that NORAD TDMD of miR-346 may be mediated by different NORAD regions than are responsible for miR-346-repressed PUM2 interaction. Predicted stability of interaction with miR-346 was highest for NORAD 4104–4125 (Fig. 3D, S12D).

Importantly, in support of the TDMD hypothesis, miR-346 levels were more than 2000-fold and 100-fold increased following NORAD silencing in C42 and C42B cells, respectively (Fig. 5G, Fig. S12E), whilst levels of miR-221-3p and − 222-3p (whose primary transcript is a miR-346 target) [51] were significantly reduced (Fig. 5G). Levels of a control, non-miR-346-regulated miR (miR-361-3p) were unaltered (Fig. S12E). We calculated (based on number of NORAD copies lost and miR-346 copies gained following NORAD silencing in C42B cells, Fig. S12F), that each NORAD molecule may be capable of turnover of approximately 650 miR-346 molecules. This is consistent with previous results [59], showing that a target with TDMD sites can cause degradation of multiple miR molecules. Of note, reduced extent of siNORAD-mediated miR-346 increase was observed in 22RV1 cells, which have higher endogenous miR-346 expression (Fig. S12G). Pre-miR-346 was undetectable in C42 PC cells, but pri-miR-346, − 222/221 and -17a/18a/19a/20a were significantly decreased following NORAD silencing in C42 cells (Fig. S12H). Thus, we cannot discount NORAD modulation of Drosha-mediated pri-miR to pre-miR processing.

To confirm the requirement for identified putative TDMD sites for miR-346 degradation, both sites were mutated by site-directed mutagenesis. WT and TDMD-mutant NORAD were transfected into 22RV1 cells (selected due to their high basal miR-346 levels) and miR-346 levels assessed. It was found that mutation of TDMD sites significantly abrogated NORAD downregulation of miR-346 (Fig. 5H), while neither affected levels of a control miR (miR-361-3p). Further, WT but not TDMD-mutant NORAD was able to significantly rescue miR-346-induced DNA damage (Fig. 5J, Fig. S12J), confirming functional impact of NORAD TDMD of miR-346.

Together, these data support NORAD target-directed miR decay (TDMD) of miR-346 as a novel genome-protection mechanism and suggest that miR-346 can cause DNA damage in part independently of NORAD.

### Targeting the miR-346-NORAD Axis for prostate Cancer therapy

**Fig. 6:**
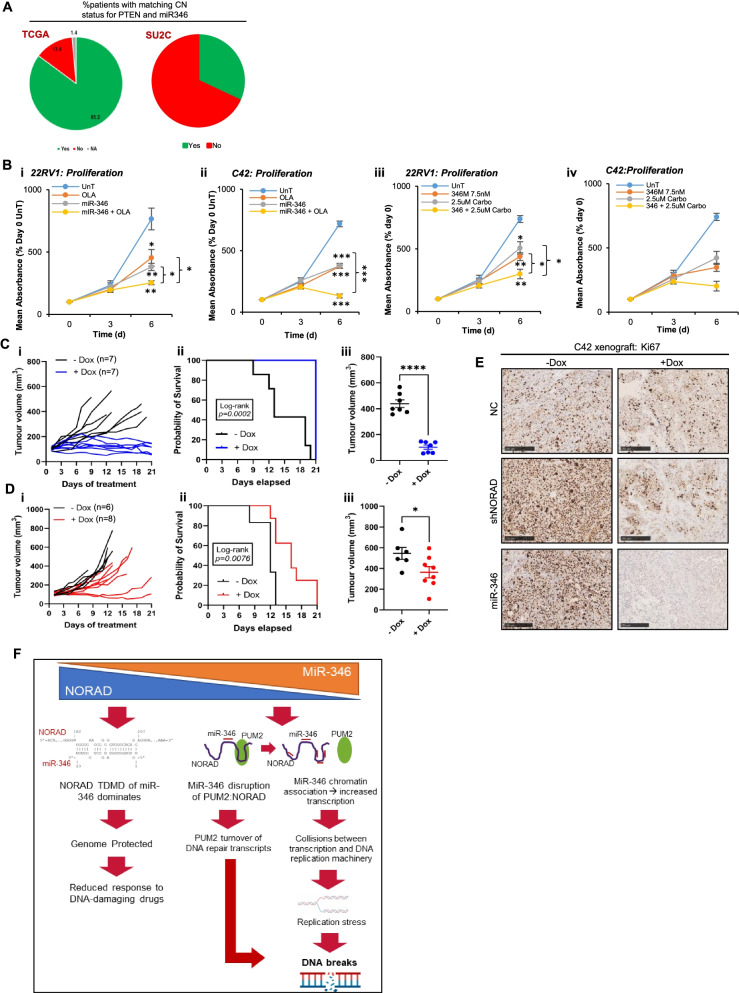
miR-346 and NORAD Modulate PC Therapeutic Response and In Vivo Tumour Growth. **A** Charts indicating percentage of PC patients from the indicated cohorts demonstrating matching PTEN and MIR346 copy number status. **B** SRB assay analysis of PC cell proliferation in response to miR-346 transfection (7.5 nM) ± olaparib PARP inhibitor (i,ii - 5 μM) or carboplatin (iii,iv – 2.5 μM) in 22RV1 (i,iii) or C42 (ii, iv) cells. **C**, **D** Relative tumour growth of C42/miR-346 (**C**) and C42/shNORAD (**D**) xenograft tumours and survival of host NSG male mice ± Dox, *n* = 7 per group. **E** Immunohistochemistry analysis of Ki67 protein levels in FFPE sections of dox-treated C42/NC, C42/miR-346 and C42/shNORAD xenograft tumours from NSG mice. Scale bar = 250 μm. **F** Model for miR-346:NORAD Regulation of DNA Damage in Prostate Cancer. Under high NORAD/ low miR-346 conditions, NORAD drives target directed microRNA decay (TDMD) of miR-346 to inhibit miR-346-induced DNA damage and prevent miR-346 inhibition of NORAD activity. High NORAD levels result in reduced response to DNA damaging agents. Under low NORAD/ high miR-346 conditions, miR-346 decreases NORAD transcript levels and disrupts NORAD:PUM2 association, liberating PUM2 to turnover DNA repair transcripts. In addition, miR-346 associates with chromatin to accelerate transcription. This results in R-loop formation and increased collisions between transcription and DNA repair machinery, leading to DNA replication stress and double-stranded DNA breaks that increase response to DNA damaging therapeutics in prostate cancer. * *P* ≤ 0.05, ** *P* ≤ 0.005, *** *P* ≤ 0.0001. See also Fig. S13–17

To assess how genomic context of miR-346 may influence its function, we investigated *MIR346* copy number (CN) status in localised PC and mCRPC. The *MIR346* gene is located less than 10 MB 5′ of *PTEN* on chromosome 10. Whilst 85% of primary cancers (TCGA-PRAD) have matching *PTEN* and *MIR346* copy number status, 102/150 (68%) of mCRPC show *MIR346* and *PTEN* on different segments (Fig. 6A, Fig. S13A), suggestive of emergence of breakpoints between the two genes during disease progression. Further, of the 48 mCRPCs with *MIR346* and *PTEN* on same segment, 4% (*n* = 2) have deep deletion (co-loss of *MIR346* and *PTEN*) compared to 22% harbouring *PTEN* deep deletion alone in the whole cohort. This suggests that when *PTEN* is deleted, mCRPC patients are less likely to have *MIR346* co-loss in the same event, potentially implying a drive to retain miR-346 gene activity during progression to mCRPC.

To evaluate the therapeutic potential of miR-346, SRB assays were performed in 22RV1 and C42 cells. miR-346 significantly repressed proliferation, at concentrations as low as 2.5 nM (Fig. 6B, Fig. S13B), and sensitized PC cells to both PARP inhibition and carboplatin chemotherapy (Fig. 6B). miR-346 also increased apoptosis (Fig. S13C) and decreased expression of cell cycle genes (Fig. S13D). To investigate the PC therapeutic relevance of miR-346 and shNORAD in a more physiologically-relevant setting, C42 cells stably-expressing doxycycline-inducible GFP-tagged pre-miR-346 or shNORAD or RFP-tagged negative control (NC) were used to generate subcutaneous xenografts in immunodeficient mice. After tumour establishment, mice were randomly assigned to Dox or vehicle treatment. Dox-induction of NC miR did not significantly alter tumour growth, survival or end-point tumour volume (Fig. S14A-C), nor expression of apoptosis (cleaved Caspase-3) markers (Fig S14Fiv, Fig. S15), although protein levels of Ki67 proliferation marker were reduced (Fig. 6E, Fig S14Fi). Induction of miR-346 expression (145-fold versus untreated tumours – Fig. S14D) was found to inhibit xenograft growth in all animals, with complete tumour regression (end-point tumour volume lower than pre-treatment tumour volume) observed in three of seven treated mice (Fig. 6C). miR-346 conferred a significant survival benefit: in the treated group, all mice survived to experiment end-point, whilst all vehicle-treated mice were culled prior to experiment end due to tumour volume (Fig. 6Cii). Tumours with miR-346-induction were significantly smaller at end-point versus vehicle-treated tumours (Fig. 6Ciii), showing significantly decreased protein levels of proliferation marker, Ki67, and elevated apoptotic marker, cleaved Caspase-3 (Fig. 6E, Fig S14Fii, v, Fig. S16) Doxycycline-induction of shNORAD significantly repressed xenograft tumour growth, increased survival and reduced end-point tumour volume (Fig. 6D), despite only 20% reduction in NORAD levels (Fig. S14E). Significantly decreased Ki67 and increased cleaved Caspase-3 levels were observed in tumours from dox-treated versus vehicle-treated mice (Fig. 6E, Fig. S14 Fiii,vi, Fig. S17). Importantly, miR-346 levels were increased in dox-treated shNORAD tumours versus untreated, despite modest decreases in intra-tumoural NORAD levels, consistent with reduced TDMD of miR-346 by NORAD (Fig S14Div). In addition, NORAD levels were significantly increased in dox-treated C42/miR-346 xenografts as compared to untreated tumours (Fig S14Eii), potentially illustrative of NORAD upregulation as an adaptive response to chronic miR-346 expression. Together, these data indicate that miR-346 is highly effective in repressing tumour xenograft growth as a monotherapy, and may show additive or synergistic effects in combination with DNA-damaging therapeutics such PARP inhibitors or chemotherapeutics. This may be particularly effective in the context of decreased NORAD observed in advanced mCRPC, and in transcriptionally-hyperactive cancer cells.

## Discussion and conclusions

In this study, we present evidence that interaction between miR-346 and the lncRNA NORAD regulates DNA damage in PC. miR-346 induces potent DNA damage through two mechanisms: it disrupts NORAD sequestration of PUM2, liberating it to turnover DNA repair transcripts, but also interacts with chromatin to increase transcription initiation, leading to R-loop formation, DNA replication stress and DNA damage checkpoint activation. It is associated with improved PC survival.

Conversely, NORAD drives TDMD of miR-346 as a novel genome protection mechanism. High NORAD in primary PC is associated with poor PC patient survival but its levels are decreased in mCRPC compared to matched primary HSPC; its loss represents a potential therapeutic vulnerability to be exploited through miR-346 administration. Indeed, as well as inducing in vivo tumour regression as a monotherapy, miR-346 sensitises PC cells to PARPi and chemotherapy.

There has been increasing interest in links between miR/ncRNA activity and DDR in recent years: it is now well-appreciated that miR biogenesis proteins are required for proficient DNA repair [60–64], and small and long ncRNA molecules have been identified in close proximity with DNA breaks [65]. Use of NGS and elegant reporter systems provides compelling evidence for Dicer-dependent transcription of damage-induced small RNAs (diRNAs) at exogenous DNA damage sites [62, 66]. This can be mediated by DNA-damage induced lncRNAs (dilncRNAs), which act both as a precursor source of diRNAs and as scaffolds for their recruitment. A common theme emerging from such studies is that both diRNAs and dilncRNAs *promote* DNA repair in a transcription-dependent manner in mammalian cells, but that the ability of diRNAs to increase repair of endogenous DSBs in non-repetitive genic regions remains controversial [67]. In contrast, ours is the first description of an endogenous nuclear-localised miR causing rapid, genome-wide DNA damage through chromatin association.

miR-346 is unusual among miRs in being nuclear, more so for being chromatin-associated. AGO-PAR-CLIP-seq analysis [51] of PC cells identifies many nucleus-retained intronic and intergenic miR-346 binding sites, supporting its functionality within this cellular compartment. Mechanisms proposed for miR nuclear localisation and retention include presence of nuclear localisation sequences, complete biogenesis within the nucleus, and active shuttling between nuclear and cytoplasmic compartments [68]. Given that NORAD is largely cytoplasmic but at least 45% of miR-346 is chromatin-associated, the latter mechanism may be most likely, particularly since miR-346 lacks the consensus ASUS sequence (S = cytosine or guanidine) present in approximately one third of nuclear miRs [69]. Indeed, cytoplasmic retention may represent an important facet of NORAD repression of miR-346 DNA-damaging activity. Since different RBPs are hypothesised to drive nuclear transport of different miR subsets (including those lacking known NLSs, such as miR-21 and miR-29a [70]), determination of protein interactors of miR-346 in different NORAD contexts will be important. Nuclear retention may also be guided by seed complementarity within nuclear RNA or DNA targets and is thus likely to be highly cell type-specific.

Formation of R-loops in response to miR-346, and the requirement for active transcription for miR-346-induced DNA damage, which was enriched at gene promoters, suggests that miR-346 may accelerate transcription initiation. and that such highly-aberrant transcription may lead to collisions between DNA replication and transcriptional machinery, leading to DNA replication stress and DSBs. Despite this initial transcription initiation ‘pulse’, resultant replication stress ultimately causes transcriptional repression, since miR-346 DSBs are enriched in miR-346-downregulated, but not -upregulated genes.

This is not the first description of transcriptional regulation by miRs: miR-744-5p and miR-466d-3p increase *CCNB1* expression through interaction with seed complementary sequences in the gene promoter, leading to increased histone H3 Lysine 4 trimethylation in a manner dependent on miR biogenesis proteins, Drosha, Dicer, AGO1 and AGO2 [71]. However, the precise mode by which miR-346 interacts with DNA remains unknown. The sheer prevalence of miR-346 breaks (91% of genes) and their enrichment at binding sites of diverse TFs may argue against simple direct sequence complementarity. Indeed, only minimal enrichment of the top-identified binding motif (for NKX3.1) was found at miR-346 DSBs as compared to background. Instead our findings may support a model whereby miR-346 binding is determined by local 3D DNA topography and/or indirect association with DNA via additional RNA/protein factors such as PolII and RISC. RISC-mediated miR:DNA association is reported to require proximal promoter-associated non-coding transcripts [72]: the requirement of transcription for miR-346-induced DNA damage is notable in this context. Alternatively, miRs have been shown to interact with complementary ssDNA sequences at promoters [73], which could also explain detection of DNA:RNA hybrids following miR-346 transfection.

A further important finding is that miR-346 DSBs are strongly enriched at binding sites of some of the most highly-active TFs in PC, including AR. Indeed, numbers of miR-346-induced DSBs are greater at ARE-containing versus non-ARE-containing promoters. Given that AR is a major transcriptional driver of pro-proliferative, pro-survival pathways, and a therapeutic target in PC, this raises the possibility that miR-346 induction of DSBs at AR binding sites could potently downregulate AR target gene expression. This provides a robust rationale for exploring therapeutic combination of miR-346 with androgen-deprivation therapies (ADT). Such a therapy should be unaffected by common mechanisms of ADT resistance, such as AR amplification, activation of AR transcriptional programmes by other TFs, and expression of constitutively-active AR transcript variants.

The observation that miR-346-induced DNA damage can be rescued by NORAD provides evidence in support of a strong reciprocal regulatory relationship between the two molecules, further underpinned by their convergence on DNA replication, cell cycle and DNA damage response pathways and miR-346 downregulation of 29 of 33 previously-identified NORAD-upregulated transcripts. The observed reduced survival of patients with high expression of NORAD may be linked to reduced response to radiotherapy. Survival analysis used data from radical prostatectomy cohorts, for whom radiotherapy prior to surgery is common. Given NORAD’s essential role in promoting genome integrity, it is logical to speculate that it may oppose radiotherapy-induced DNA damage and cell death. In this context, it will be interesting to investigate the ability of shNORAD to sensitise PC models to radiotherapy (and determine how this relates to DNA repair proficiency - 30% of tumours harbour DDRD [36]), and to examine the potential of NORAD as a predictive biomarker. In addition, the strong correlation between NORAD expression/−activity and DDR in primary PC supports a critical role for NORAD in DNA repair in PC, in agreement with data from other cell types [29–31]. However, it is particularly intriguing that this correlation is completely lost in mCRPC. This may be explained by an uncoupling of NORAD from DDR in the context of the overwhelming genetic aberrations often observed in mCRPC.

Seminal work from the Ulitsky and Mendell laboratories first described NORAD as containing highly-conserved repeat units capable of binding at least 17 PUM1/PUM2 protein molecules to prevent their turnover of critical DDR and mitosis-associated transcripts [29, 30]. Although miR-346 only modestly reduced NORAD levels, it strongly inhibited interaction between NORAD and PUM2, supporting the relevance of the DDR-modulatory NORAD:PUM2 interaction [29, 30] to the PC context. A 6mer site at NORAD 1996–2001 immediately adjacent to a PRE was identified as responsible for miR-346 disruption of the NORAD:PUM2 interaction. Whether such a regulatory mechanism (likely to disrupt PUM2 binding at only one of NORAD’s eight repeat units) is active under physiologically low miR-346/ high NORAD conditions is unclear. However, it is plausible that miR-346 binding at this site is sufficient to disrupt PUM2 binding through steric hindrance. Alternatively, miR-346 binding may alter the wider 3D configuration of NORAD to modulate PUM2 binding at different sites, or may recruit additional factors to disrupt NORAD:PUM2 association.

Our findings also add another dimension to NORAD’s role as ‘defender of the genome’, and may go some way to explaining why its loss causes such extensive chromosomal instability: in addition to sequestering PUM2, and formation of a genome-protective ribonuclear protein complex with RBMX and TOP1 [31], NORAD initiates decay of DNA damaging miR-346 via TDMD [59, 74–77]. This is likely to be a particularly efficient mechanism of genome protection, since NORAD TDMD sites are five-fold higher than miR-346 copies in unperturbed PC cells, in keeping with prior observations that miR*/*target ratio, as well as complementarity, determines extent of TDMD [59, 74, 75]. Indeed, absolute quantification estimated that each NORAD molecule may be capable of turnover of up to 650 miR-346 copies. This is consistent with, albeit more dramatic than, the observation that one molecule of the lncRNA, Cyrano, drives loss of approximately 17 copies of miR-7 in mouse cerebellum [78]. Presence of NORAD TDMD sites distant from PREs suggests that different NORAD regions are responsible for TDMD to those via which miR-346 disrupts NORAD:PUM2 interaction. An important question raised by this study is the extent to which miRs other than miR-346 can induce DNA damage. Given the importance of NORAD in regulating DDR, its high levels of cellular expression, its strong correlation with miR activity pathways in mCRPC, and that it is one of the most heavily miR-bound transcripts in the PC genome [51], it is tempting to speculate that a key mechanism of action for this molecule may be in sequestering and targeting a subset of DNA-damaging miRs for degradation.

We propose that the loss of NORAD in mCRPC could represent a therapeutic vulnerability to be exploited through administration of miR-346 to induce intolerable DNA damage, particularly since miR-346 decreases during PC progression and induces in vivo tumour regression. We further showed that miR-346 can sensitise PC cells to other DNA damaging agents such as chemotherapy and PARPi, and it will be important to assess these combinatorial effects in patient-relevant models of different DDRD contexts. Given its mode of action, we suggest that miR-346 may be most successful as a therapeutic in transcriptionally-hyperactive cellular contexts, as frequently observed in cancer. In support of this, miR-346 showed minimal induction of DNA damage in non-cancerous PNT1a prostate cells.

In summary, our data support the model illustrated in Fig. 6F, whereby, under conditions of high NORAD and low miR-346, NORAD target directed microRNA decay of miR-346 dominates to prevent miR-346-induced DNA damage, constituting a novel mechanism of genome protection. When NORAD levels are reduced and/or miR-346 increased, miR-346 decreases NORAD transcript levels and disrupts NORAD:PUM2 interaction, liberating PUM2 to turnover DNA repair transcripts. In addition, miR-346 associates with chromatin, resulting in a pulse of aberrant transcription initiation. This results in R-loop formation and increased collisions between transcription and DNA replication machinery, leading to replication stress and extensive DNA DSBs at binding sites of PC-critical TFs such as AR. Thus the relative abundance of NORAD and miR-346 determines DDR and genome stability in PC. This is of direct clinical relevance, since high NORAD is associated with reduced PC survival: its loss in mCRPC versus CSPC represents a potential therapeutic vulnerability to be exploited through delivery of miR-346, potentially in combination with chemotherapy or PARP inhibition*.*

## Supplementary Information

**Additional file 1.**

**Additional file 2.**

**Additional file 3.**

## Data Availability

The datasets used and/or analysed during the current study are available from the corresponding author on reasonable request.

## Abbreviations

3’UTR: 3′ untranslated region
ADT: Androgen deprivation therapy
AR: Androgen receptor
ARE: Androgen response element
CN: Copy number
CSPC: Castration-sensitive prostate cancer
DDR: DNA damage response
Dox: Doxycycline
DSB: Double-strand break
HR: Homologous recombination
IHC: Immunohistochemistry
lncRNA: Long non-coding RNA
mCRPC: Metastatic castration-resistant prostate cancer
miR: MicroRNA
MSI: Microsatellite instability
NAS: NORAD activity score
ncRNA: Non-coding RNA
NEPC: Neuroendocrine prostate cancer
NORAD: Non-coding RNA activated by DNA damage
PARPi: PARP inhibitor
PC: Prostate Cancer
PRE: Pumilio-2 recognition element
PUM2: Pumilio-2
RNA-ISH: RNA in situ hybridisation
SNP: Single nucleotide polymorphism
SRB: Sulphorhodamine B
TAD: Topologically-associating domain
TDMD: Target-directed microRNA decay
TF: Transcription factor
TSS: Transcription start site
