# Supplementary material for: A non-coding RNA balancing act: miR-346-induced DNA damage is limited by the long non-coding RNA NORAD in prostate cancer
Source: Mol Cancer. 2022 Mar 22;21:82. doi: 10.1186/s12943-022-01540-w (PMC8939142; doi:10.1186/s12943-022-01540-w)
Supplement: Supplementary file 1 — Additional file 1. [file 12943_2022_1540_MOESM1_ESM.pdf]

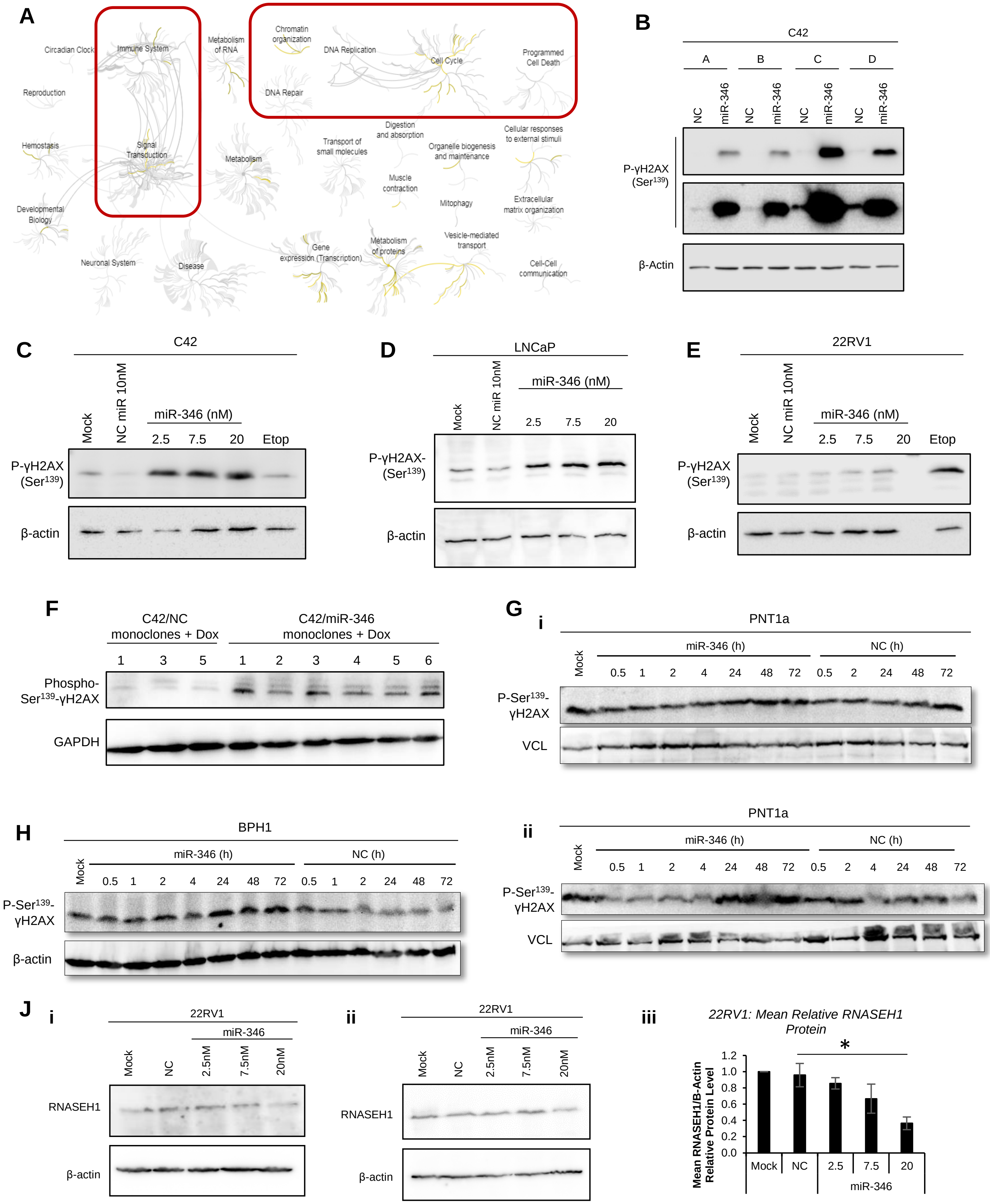

Figure S1: MiR-346 Induces Dose-Dependent DNA Damage in PC Cells. *A)* Pathway analysis of validated miR-346-bound transcripts identified by AGO-PAR-CLIP-seq in PC cell lines[51]. *B-F)* Western blot assay analysis of phospho-γH2AX protein levels (DNA damage marker) in: *B,C)* C42, *D)* LNCaP and *E)* 22RV1 PC cells transfected with indicated concentrations of miR mimics for 96h *(B,D)* or 72h *(C,E)*, and *F)* 72h 100ng/ml doxycycline-treated C42 monoclones expressing tetracycline-inducible pre-miR-346 or NC pre-miR. Numbers represent individual monoclones. *G,H)* Western blot analysis of phospho-γH2AX protein levels in PNT1A *(G)* and BPH1 *(H)* cells transfected with 20nM miR-346 or NC for indicated durations. Etoposide (10µM) was used as a positive control for DNA damage *(C,E)*. *J)* Western blot analysis of RNASEH1 protein levels in 22RV1 cells treated with 10nM NC mimic or 2.5, 7.5 or 20nM miR-346 for 72h. Densitometry was performed using ImageJ. β-actin *(B-E, H)*, GAPDH *(F)* and Vinculin *(G)* were used as loading controls. Biological replicates relating to **Fig 1** are shown. * *P*≤0.05.

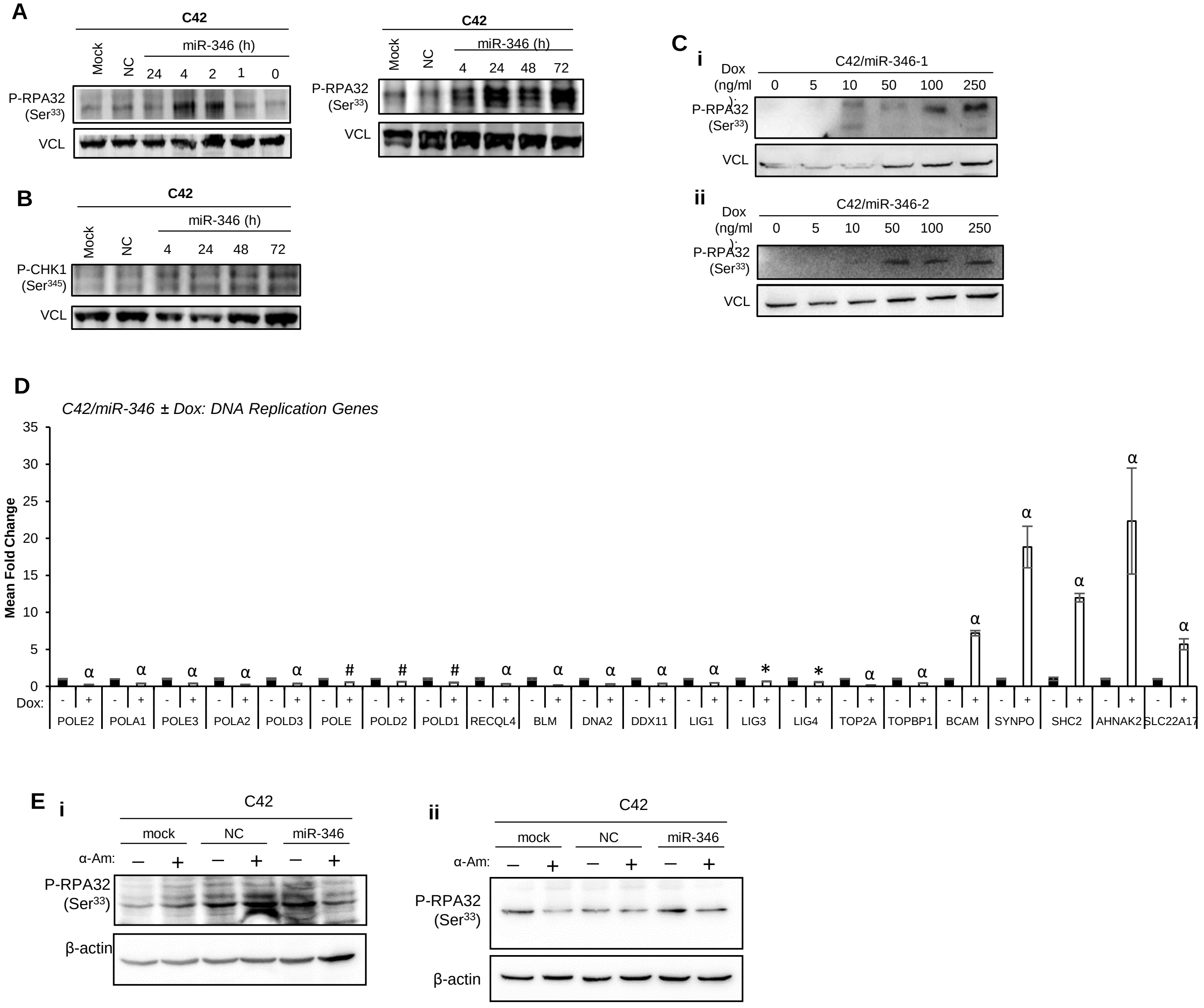

Figure S2: MiR-346 Induces Replication Fork Stalling and Slowing. *A,B)* Western blot analysis of *A)* phospho-RPA32(Ser33) and *B)* phospho-CHK1(Ser345) in C42 cells transfected with 20nM miR-346 mimic for indicated durations. C*)* Western blot analysis of phospho-RPA32(Ser33) protein levels in C42/miR-346 monoclones treated with indicated doxycycline concentrations for 72h. Vinculin (VCL) was used as a loading control and results for two independent monoclones are shown. *D)* RNA-seq analysis of DNA replication gene expression in C42/miR-346 cells ± Dox (72h). Additional miR-346-upregulated genes are shown. *E)* Western blot analysis of phospho-RPA32(Ser33) protein levels in C42 cells treated with 10nM miR-346 ± 5μM alpha-amanitin for 16h. * $P \leq 0.05$, # $P \leq 0.005$, α $P \leq 0.0001$. See also **Fig 1**.

NC

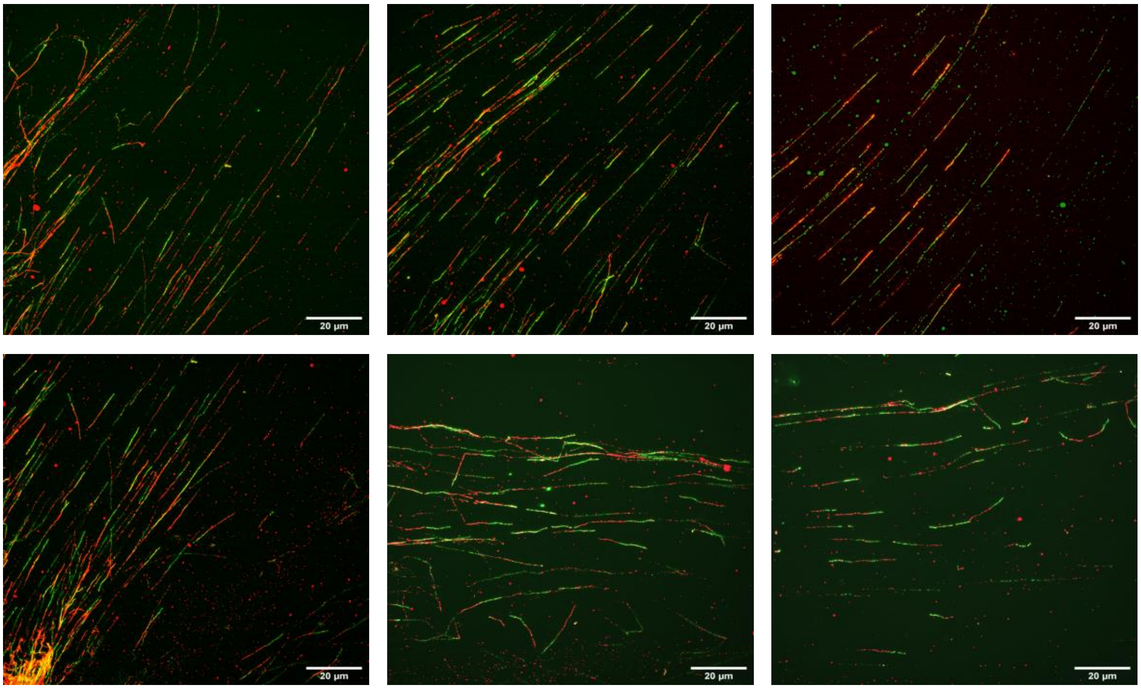

miR-346

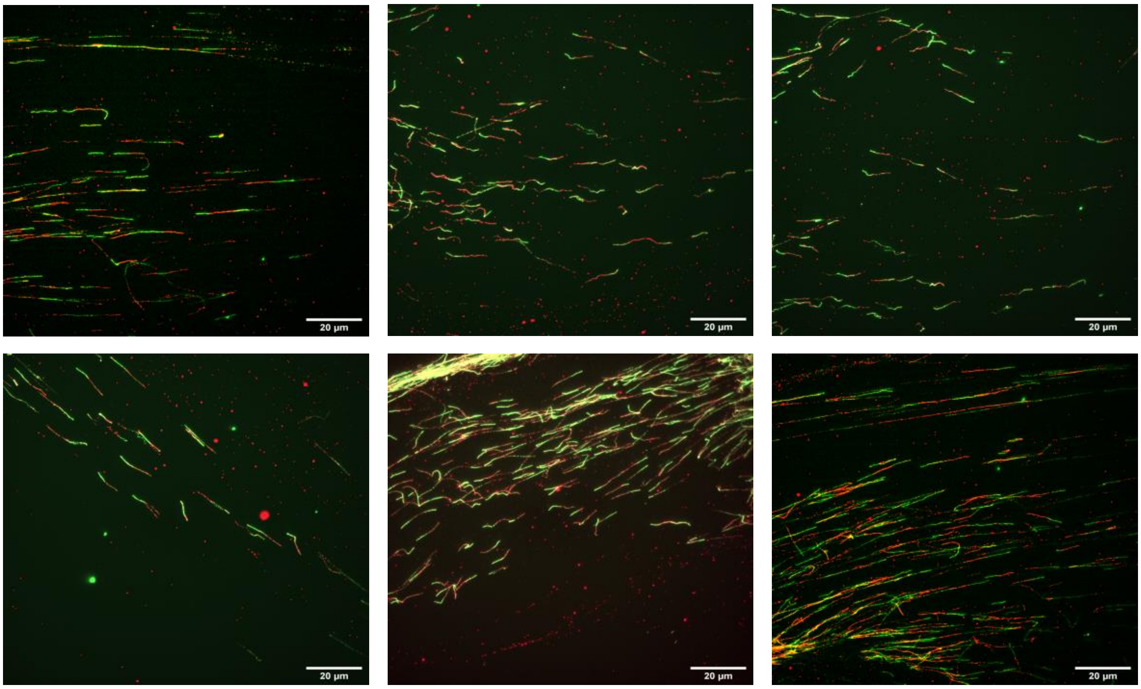

5HU

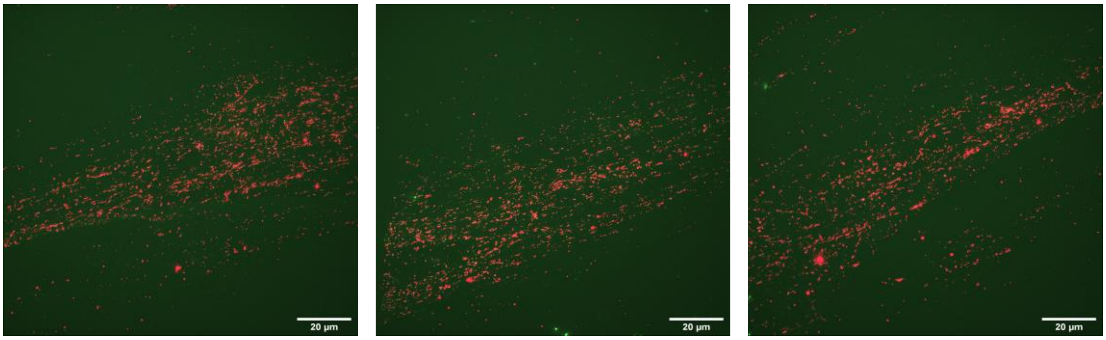

Fig S3: DNA fibre assays analysis of C42 cells following transfection with NC or miR-346 (24h), or 40min 5HU treatment. A minimum of one hundred fibres were quantified for each measurement and different replication events quantified in ImageJ as described (Halliwell J *et al.* 2020). Fields were selected using one fluorescence channel only for the avoidance of bias. Scale bars = 20µm. Additional images from independent biological repeats relating to **Fig 1H** are shown.

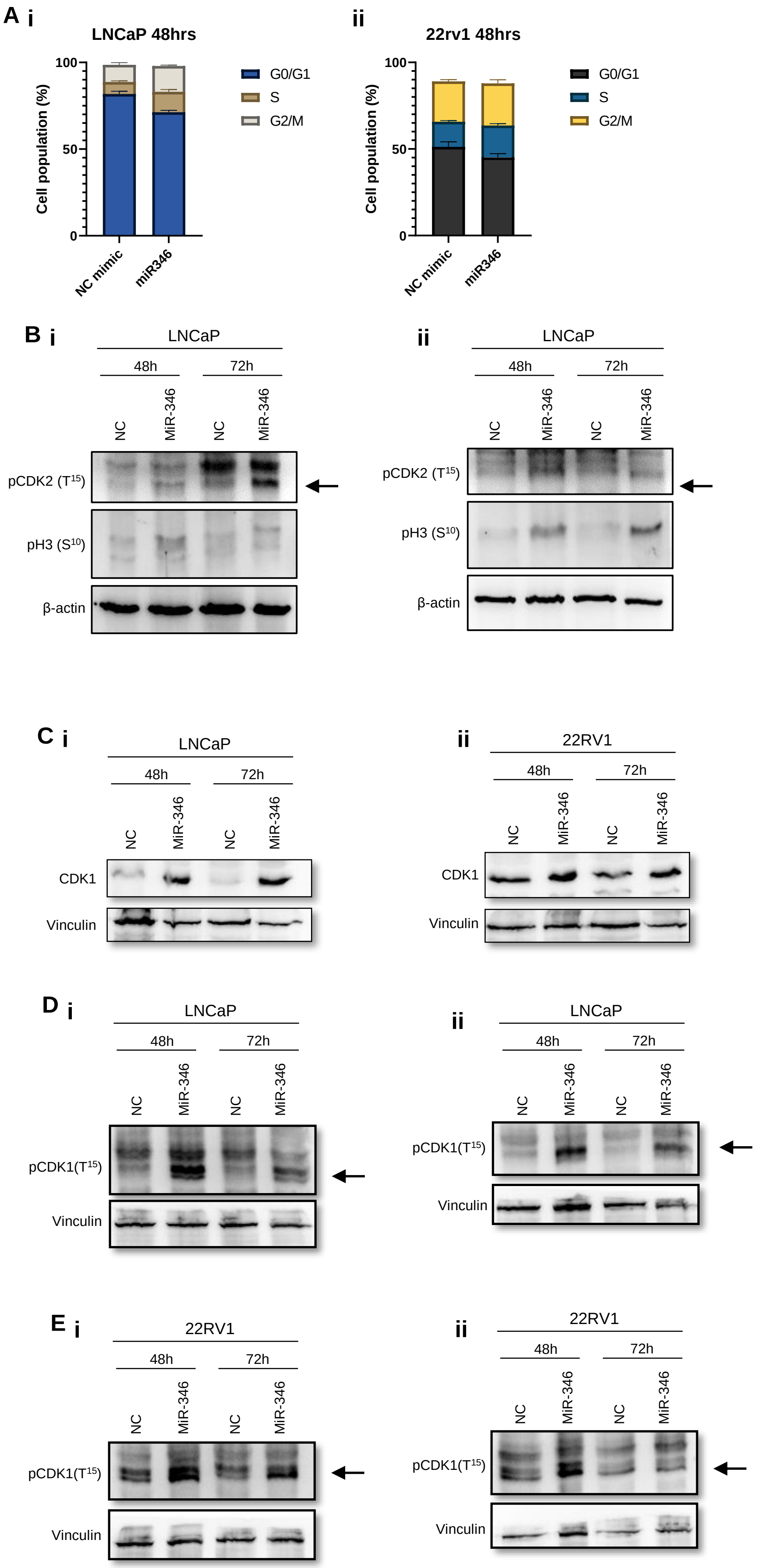

Fig S4: MiR-346 Causes Prostate Cancer Cell Cycle Arrest in S- and M-phase. *A)* Flow cytometric analysis of propidium iodide-stained LNCaP *(i)* and 22RV1 *(ii)* cells transfected with miR-346 or NC mimic for 48h. *Columns:* mean ± SEM for three independent experiments. *B-E)* Western blot analysis of *(B)* phospho-CDK2(T^{15}) and phospho-Histone H3(S^{10}), *(C)* total CDK1 and *(D,E)* phospho-CDK1(T^{15}) in *(B,D)* LNCaP, *(C)* LNCaP and 22RV1 and *(E)* 22RV1 cells transfected with miR-346 or NC mimic for 48 and 72h. β-actin *(B)* and Vinculin *(C-E)* were used as loading controls. Biological replicates relating to **Fig 1** are shown.

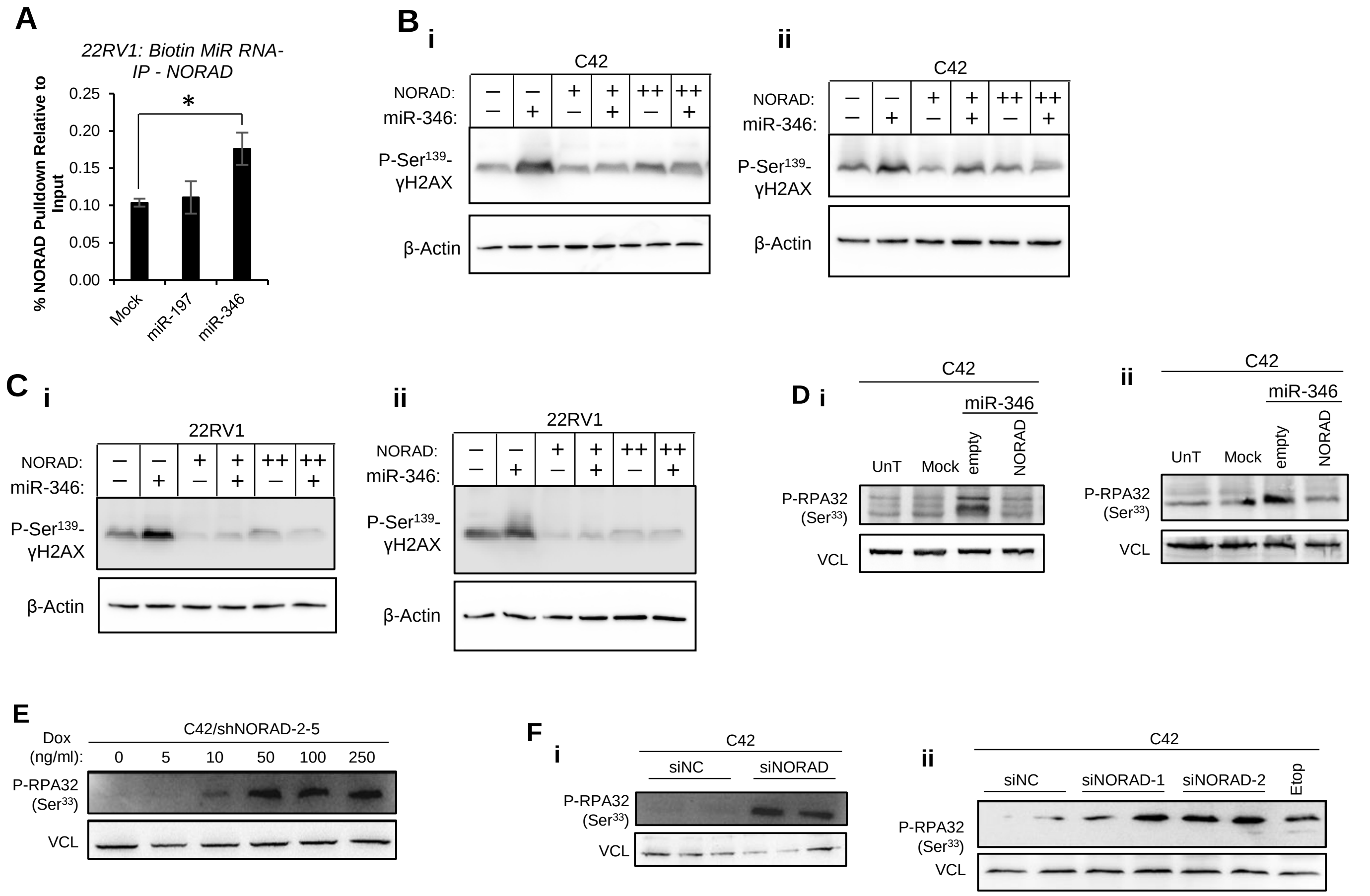

Figure S5: NORAD Interacts with MiR-346 and Rescues MiR-346-Induced DNA Damage in PC Cells. *A)* AGO2/biotin-miR pulldown analysis of NORAD association with miR-197 and miR-346 within AGO2-containing RISC complex of 22RV1 cells. *B,C)* Western blot assay analysis of *B)* C42 or *C)* 22RV1 cells transfected with miR-346 or NC mimic (10nM) ± NORAD or empty control plasmid for 72h. *D)* Western blot analysis of phospho-RPA32(Ser33) protein levels in C42 cells transfected with miR-346 (10nM) ± NORAD or empty control plasmid for 72h. *E)* Western blot analysis of phospho-RPA32(Ser33) protein levels in C42/shNORAD monoclone #2-5 treated with indicated doxycycline concentrations for 72h. *F)* Western blot analysis of phospho-RPA32(Ser33) protein levels in C42 cells transfected with 20nM siNC or siNORAD for 72h. β-Actin *(B,C)* and Vinculin *(D-F)* were used as loading controls. Biological replicates relating to **Fig 2** are shown.

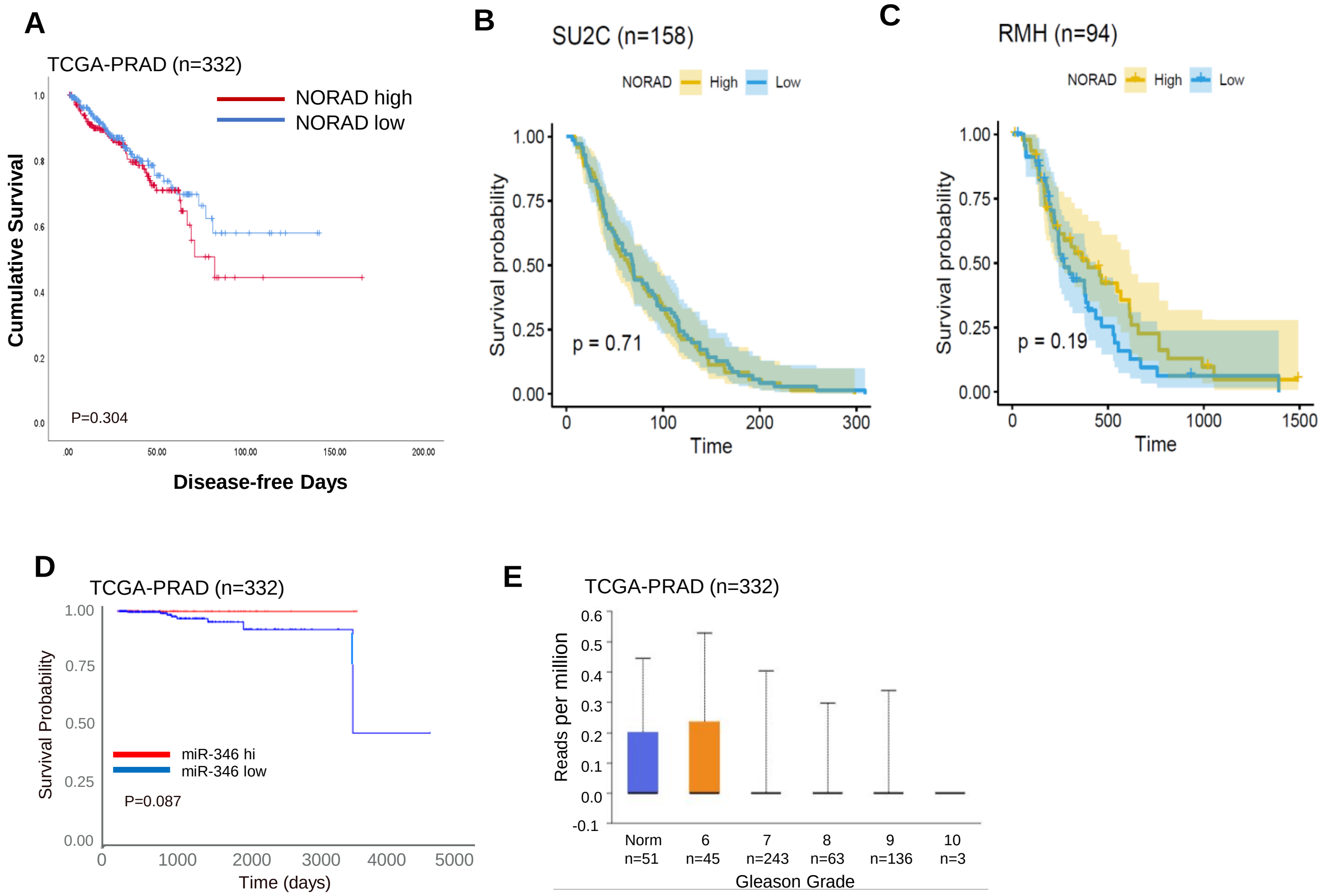

Figure S6: NORAD and MiR-346 are Associated with Prostate Cancer Survival. *A-C)* Correlation of NORAD transcript levels with patient survival in TCGA-PRAD (n=332 - *A*), SU2C mCRPC (n=158 – *B*) and Royal Marsden Hospital (RMH) mCRPC (n=94) patient cohorts. *D)* Correlation of miR-346 with patient survival in TCGA-PRAD data set (n=332). *E)* MiR-346 expression in tumours of different grades from TCGA-PRAD (n=332). mCRPC = metastatic castration-resistant prostate cancer.

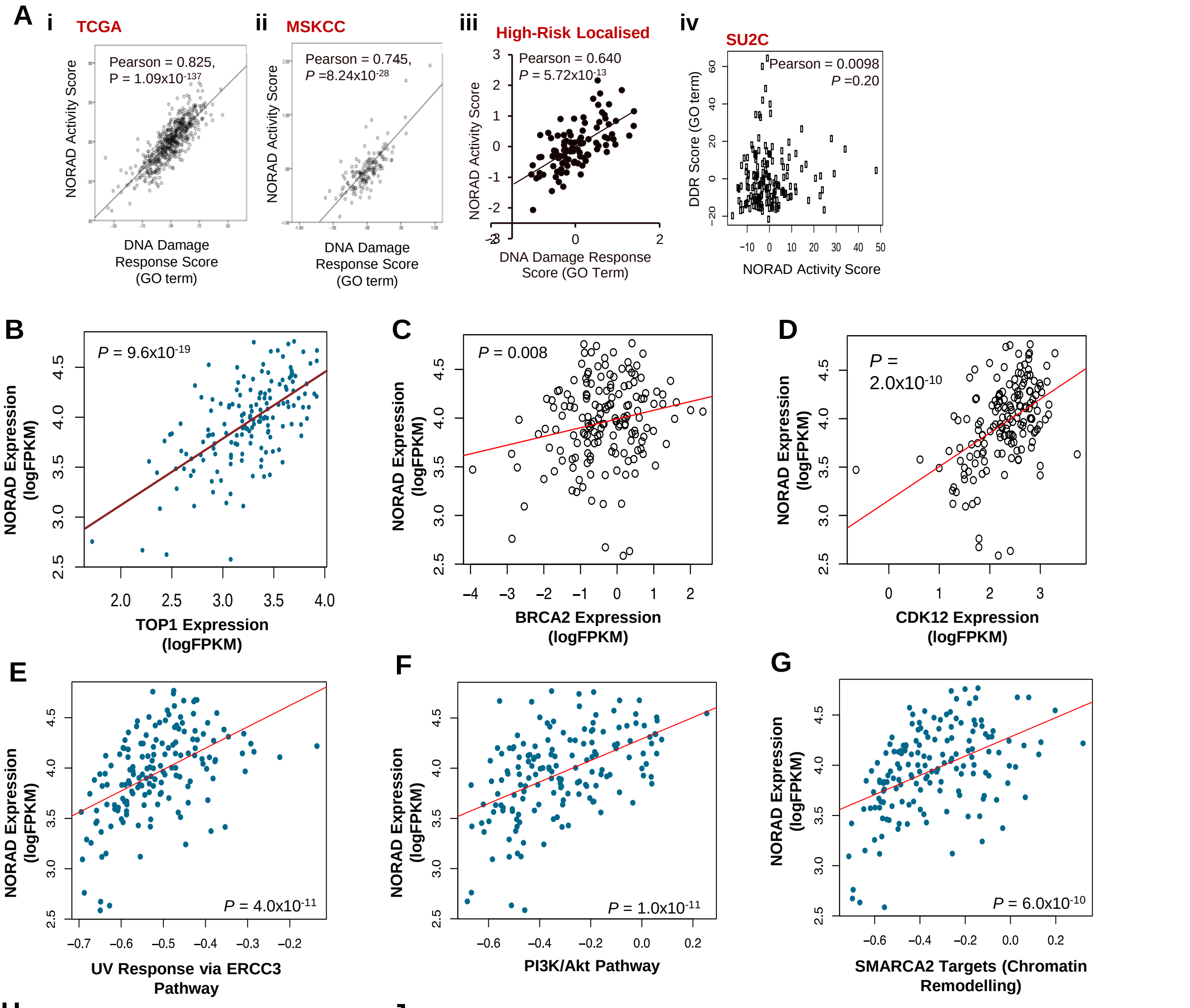

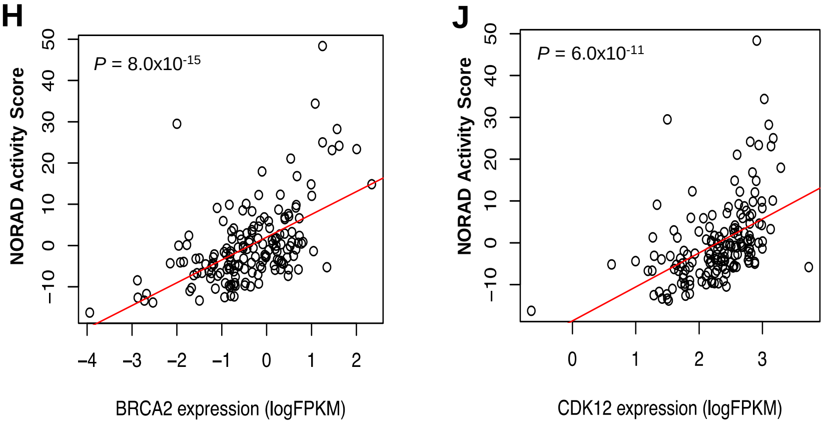

Figure S7: NORAD Activity Correlates with DNA Damage Response Pathways in Advanced Prostate Cancer. *A)* Correlation between DNA damage response score and NORAD activity score (NAS) in indicated PC patient tumour gene expression data sets[36,51,76]. *B-J)* Correlation between NAS and DDR-associated transcripts and pathways in the SU2C mCRPC data set[51]. *K)* NORAD RNA *in situ* hybridisation analysis of NORAD RNA copies in epithelial and stromal compartments of matched castration-sensitive (CSPC) and metastatic castration-resistant PC (mCRPC), n=23. **See also Fig 2.**

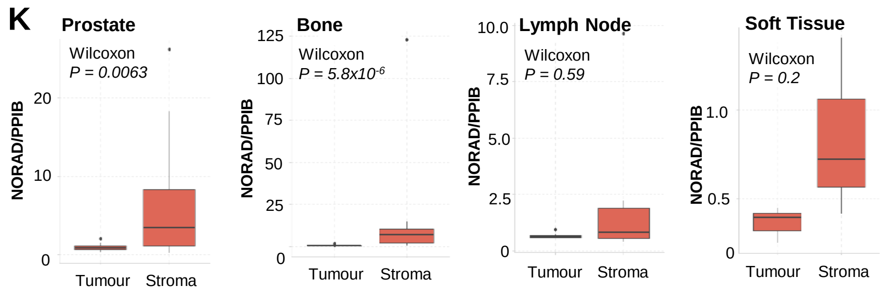

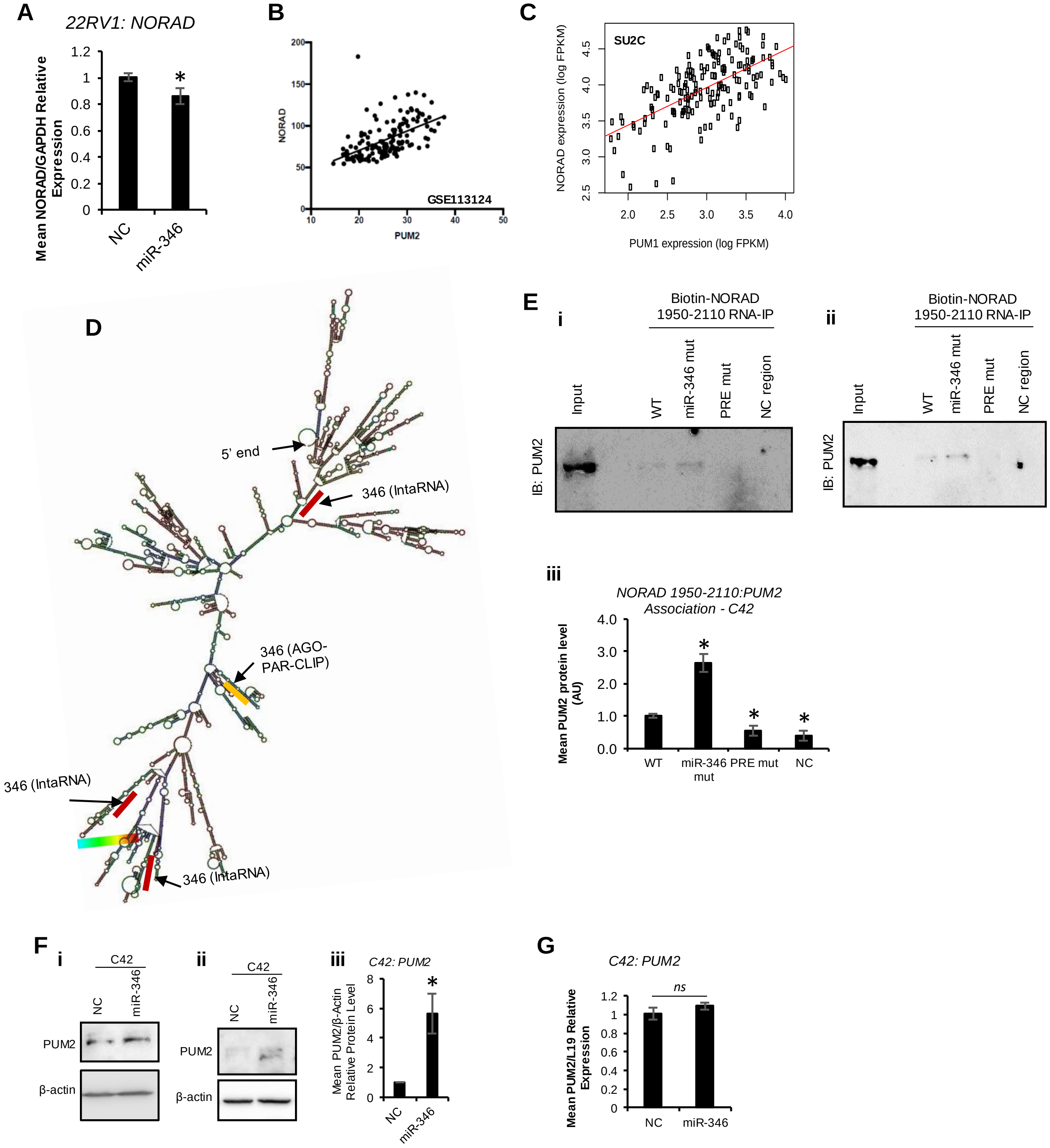

Figure S8: MiR-346 Disrupts NORAD:PUM2 Interaction to Increase DNA Damage in Prostate Cancer. *A)* qRT-PCR analysis of NORAD expression in 22RV1 cells transfected with miR-346 (10nM) for 72h. Mean ± SEM for three biological repeats performed in technical triplicate is shown. Data are presented relative to GAPDH expression. *B)* NORAD correlates with PUM2 expression in primary PC (GSE113124), *C)* NORAD correlates with PUM1 expression in metastatic PC (SU2C). *D)* Predicted secondary structure of NORAD RNA, indicating miR-346 binding sites. *E)* Western blot analysis of PUM2 protein levels in biotin-NORAD 1950-2110 (WT or mutant, as indicated) immunoprecipitates from C42 cells. Biological replicates relating to Fig 4E are shown. *F)* Western blot analysis of PUM2 protein levels in levels in C42 cells transfected with miR-346 or negative control miR (20nM) for 72h. β-actin was used as a loading control and independent biological replicates relating to Fig 4F are shown. *E,F)* Quantification was performed using ImageJ. *G)* qRT-PCR analysis of PUM2 expression in C42 cells transfected with miR-346 (20nM) for 72h. Mean ± SEM for three biological repeats performed in technical triplicate is shown. Data are presented relative to L19 expression. * $P \leq 0.05$. **See also Fig 3.**

A

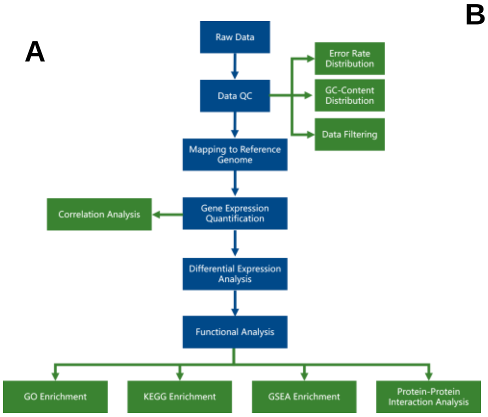

HISAT2 was used to map filtered sequenced reads to reference genome.

B

Sample	raw_reads	clean_reads	raw_data(G)	clean_data(G)	error_rate(%)	Q20(%)	Q30(%)	GC (%)
M346_1_D	39782731	39396167	11.9	11.8	0.02	98.17	94.68	53.49
M346_1_N	35500371	35003424	10.7	10.5	0.02	97.98	94.41	54.61
M346_12D	34859996	33769457	10.5	10.1	0.03	97.62	93.35	51.74
M346_12N	39374665	38377558	11.8	11.5	0.03	97.7	93.77	49.98
M346_2_D	30379479	29847339	9.1	9	0.03	97.94	93.99	55.6
M346_2_N	34454289	33474875	10.3	10	0.03	97.8	93.93	50.9
shNOR12D	33819066	33278254	10.1	10	0.03	97.89	94.27	55.5
shNOR12N	40331375	39676762	12.1	11.9	0.03	97.89	94.16	54.06
shNOR4Dx	42201269	41810130	12.7	12.5	0.02	98.01	94.41	52.91
shNOR4NT	36245491	35236585	10.9	10.6	0.03	97.43	93.36	53
shNOR5Dx	38585444	38013719	11.6	11.4	0.03	97.65	93.74	54.4
shNOR5NT	43227603	42588429	13	12.8	0.03	97.85	94.1	53.91
siNC_1	34009699	33411944	10.2	10	0.03	97.72	93.87	55.13
siNC_2	31695554	31271530	9.5	9.4	0.02	97.93	94.29	54.38
siNC_3	40676741	40090360	12.2	12	0.03	97.88	94.16	53.81
siNORAD1	32768255	32250375	9.8	9.7	0.03	97.7	93.73	52.07
siNORAD2	35197322	34671072	10.6	10.4	0.02	97.96	94.23	52.05
siNORAD3	36384596	35702698	10.9	10.7	0.03	97.78	93.88	53

(2) Raw reads: reads count from the raw data, four rows as a unit, with statistics of reads count for every sequencing.
(3) Clean reads: Clean data are reads count filtered from raw data. Statistics method is similar with raw reads. All the following analysis is based on clean data.
(4) Raw bases: Base number of raw data. (number of raw reads) * (sequence length), converting unit to G.
(5) Clean bases: Base number of raw data after filtering. (number of clean reads) * (sequence length), converting unit to G.
(6) Error rate(%): base error rate of whole sequencing.
(7) Q20(%): The percentage of the bases whose Q Phred values is greater than 20. (Number of bases with Q Phred value > 20) / (Number of total bases) *100.
(8) Q30(%): The percentage of the bases whose Q Phred values is greater than 30. (Number of bases with Q Phred value > 30) / (Number of total bases) *100.
(9) GC content(%): The percentage of G&C base numbers of total bases.(G&C base number) / (Total base number)*100.

C

Sample name	M346_1_N	M346_12N	M346_2_N	M346_1_D	M346_12D	M346_2_D	shNOR12N	shNOR4NT	shNOR5NT	shNOR12D	shNOR4Dx	shNOR5Dx	siNC_1	siNC_2	siNC_3	siNORAD1	siNORAD2	siNORAD3
Total reads	70006848	76755116	66949750	78792334	67538914	59694678	79353524	70473170	85176858	66556508	83620260	76027438	66823888	62543060	80180720	64500750	69342144	71405396
Total mapped reads	66240766	72903946	63194892	65265149	53992859	47317145	75292069	66360119	80683327	61086393	78177806	70897631	63750611	59785346	76642250	61485973	66230602	68007547
Uniquely mapped reads	56987392	68227094	59149874	57597595	50492403	39848497	64918213	62100991	69449493	52708337	68444654	60804271	53774147	51341498	66391040	53261515	57083676	58404909
Multiple mapped reads	9253374	4676852	4045018	7667554	3500456	7468648	10373856	4259128	11233834	8378056	9733152	10093360	9976464	8443848	10251210	8224458	9146926	9602638
Total mapping rate	94.62%	94.98%	94.39%	82.83%	79.94%	79.27%	94.88%	94.16%	94.72%	91.78%	93.49%	93.25%	95.40%	95.59%	95.59%	95.33%	95.51%	95.24%
Uniquely mapping rate	81.40%	88.89%	88.35%	73.10%	74.76%	66.75%	81.81%	88.12%	81.54%	79.19%	81.85%	79.98%	80.47%	82.09%	82.80%	82.58%	82.32%	81.79%
Multiple mapping rate	13.22%	6.09%	6.04%	9.73%	5.18%	12.51%	13.07%	6.04%	13.19%	12.59%	11.64%	13.28%	14.93%	3.50%	12.79%	12.75%	13.19%	13.45%

(2) Total reads: total clean reads used for analysis
(3) Total mapped reads: numbers of reads being mapped on the genome, the ratio should higher than 70%.
(4) Uniquely mapped reads: numbers of reads being mapped on single position of the genome
(5) Multiple mapped reads: numbers of reads being mapped on more than one position of the genome.
(6) Total mapping rate: (mapped reads)/(total reads)*100
(7) Uniquely mapping rate: (uniquely mapped reads)/(total reads)*100
(8) Multiple mapping rate: (multiple mapped reads)/(total reads)*100

D

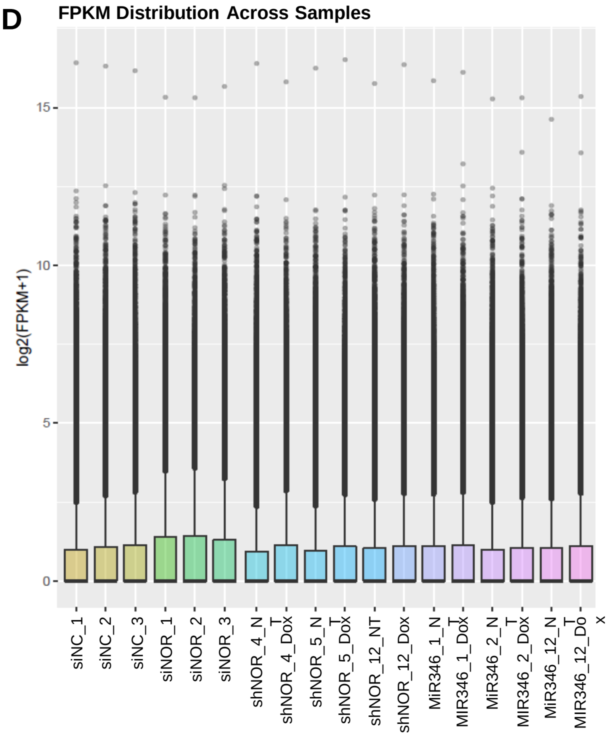

Distribution of Gene expression levels among different samples. X axis represents the name of the sample, Y axis indicates the log10(FPKM+1), parameters of box plots are indicated, including maximum, upper quartile, mid-value, lower quartile and minimum

E

Sample	Exonic	Intronic	Intergenic
MiR-346_1_N	73.16	15.85	11
MiR-346_12_N	77.54	14.75	7.71
MiR-346_2_N	81.22	11.39	7.38
MiR-346_1_D	73.27	16.25	10.48
MiR-346_12_D	74.57	16.59	8.84
MiR-346_2_D	75.75	11.72	12.53
shNOR4NT	81.35	10.61	8.05
shNOR5NT	76.67	13.06	10.27
shNOR12NT	74.27	15.05	10.68
shNOR4D	74.55	15.67	9.78
shNOR5D	71.54	17.07	11.39
shNOR12D	75.78	12.49	11.73
siNC 1	75.72	12.67	11.62
siNC 2	73.41	15.41	11.17
siNC 3	71.33	17.87	10.8
siNORAD 1	60.46	26.93	12.61
siNORAD 2	61.21	26.17	12.62
siNORAD 3	66.16	20.98	12.88

Exonic/Intonic/Intergenic Distribution of RNA-Seq Reads

Fig S9: Read Numbers, Mapping Data and Gene Expression Distribution of C42 RNA-Sequencing Data. *A)* RNA-seq data analysis pipeline. *B)* Table showing read numbers, error rate and GC content of RNA-seq reads. *C)* Table showing percentage of RNA-seq reads mapping to human genome. *D)* Distribution of gene expression levels across different samples. *E)* Percentage of RNA-seq reads mapping to exonic, intronic and intergenic regions of the human genome. Data relating to **Fig 3** are shown.

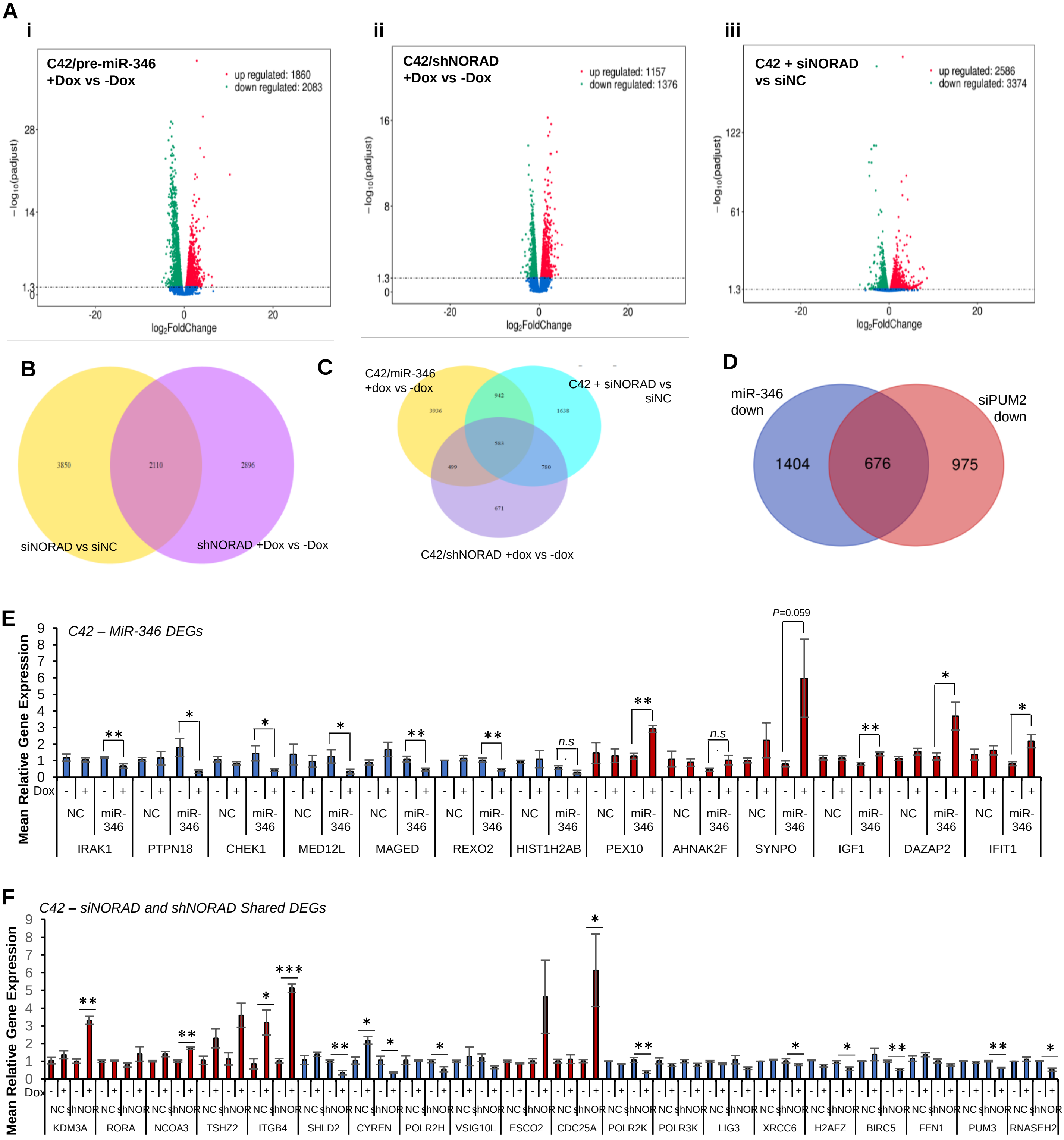

Fig S10: RNA-seq Identified MiR-346 and NORAD Regulation of DNA Replication and Repair Pathways in Prostate Cancer. *A)* Volcano plot analysis of significantly differentially-expressed genes (DEGs) identified by RNA-seq of i) C42/miR-346 ± dox, ii) C42/shNORAD ± dox, and iii) C42 ± siNORAD. *B,C,D)* Venn diagrams showing DEGs shared by siNORAD and shNORAD *(B),* miR-346, shNORAD and siNORAD *(C)*, and by miR-346 and siPUM2 *(D)*. *E,F)* qRT-PCR analysis of transcript levels of top RNA-seq-identified miR-346- *(E)* and shNORAD-/siNORAD *(F)* modulated genes. Red columns indicated genes upregulated by dox transgene induction by RNA-seq, blue columns represent genes downregulated by dox transgene induction by RNA-seq. L19 was used as a normalisation gene. *Columns:* mean ± SEM for three independent experiments performed in triplicate. * $P \leq 0.05$, ** $P \leq 0.005$, *** $P \leq 0.0001$. Data relating to **Fig 3** are shown.

A

Overlap of MiR-346-Induced DSBs with shNORAD-Induced DSBs

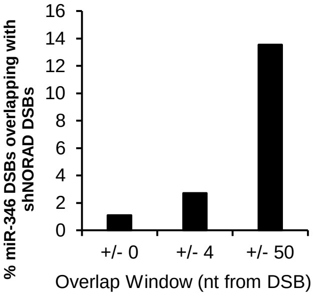

B

	Best match	P-val	log P-val	% of Targets	% of Background	Motif sequence	Alignment	Length
1	NKX3.1	1e-23071	-5.31E+04	43.17%	34.52%	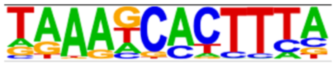	TAAAKCACTTTA<br>--AAGCACTTAA	12bp
2	NKX2.3	1e-10178	-2.34E+04	33.58%	28.14%	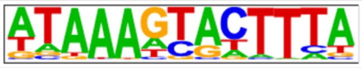	-ATAAAGTACTTTA--<br>CNTTAAGTACTTAANG	13bp
3	SREBF1	1e-13461	-3.10E+04	20.48%	15.35%	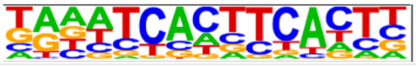	KADATCACTTCACTB<br>---ATCACCCCAC--	15bp
4	Bapx1	1e-5993	-1.38E+04	31.76%	27.62%	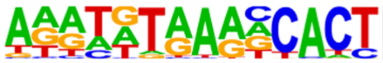	ARATDTAAANCACT---<br>-------MRSCACTYAA	14bp
5	E2F4	1e-5887	-1.36E+04	15.11%	12.07%	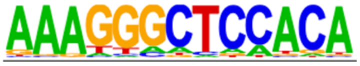	AAAGGGCTCCACA-<br>NAATGGCGCCAAAA	13bp
6	FOXJ3.2	1e-5463	-1.26E+04	8.95%	6.67%	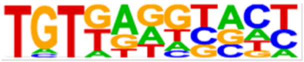	-----TGTKARGYACY-<br>NNCTTTGTTTTGNTNNN	11bp
7	ZNF528	1e-4345	-1.00E+04	8.48%	6.48%	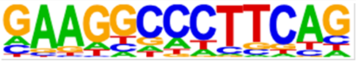	---GAAGGCCCTTCAG<br>AGGGAAGTCATTTCT-	13bp

C

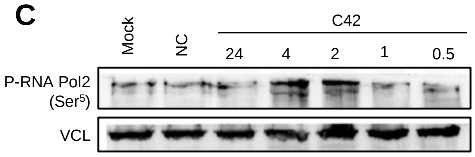

D

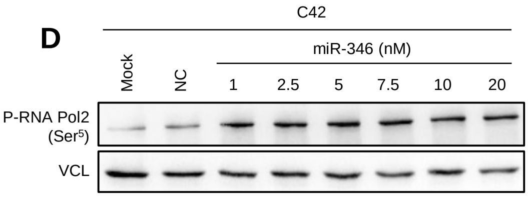

Figure S11: MiR-346-Induced DSBs Show Minimal Overlap with shNORAD-Induced DSBs, have Limited DNA Sequence Specificity, and MiR-346 Stimulates Transcription in Prostate Cancer Cells. *A)* Integration of coordinates of miR-346- and shNORAD-induced double-strand breaks (DSBs) from INDUCE-seq data reveals minimal overlap between shNORAD- and miR-346-induced DSBs. *B)* Outcome of HOMER *de novo* motif enrichment analysis searching for 10-15bp-long motifs. '% of Targets' and '% of Background' refer to the frequency of a given motif appearing in the target (DSBs in C42/miR-346 +doxycycline) or background sequence (DSBs in C42/NC –doxycycline), respectively. 'Best Match' lists TFs where similar motifs were identified from HOMER or JASPAR TFs database. *C,D)* Western blot analysis of phospho-RNA PolII (Ser5) protein levels in C42 cells transfected with 20nM miR-346 for the indicated durations *(C)*, or at the indicated miR-346 concentrations *(D)* for 72h. VCL was used as a loading control and representative images are shown. Data relating to **Fig 4** are shown.

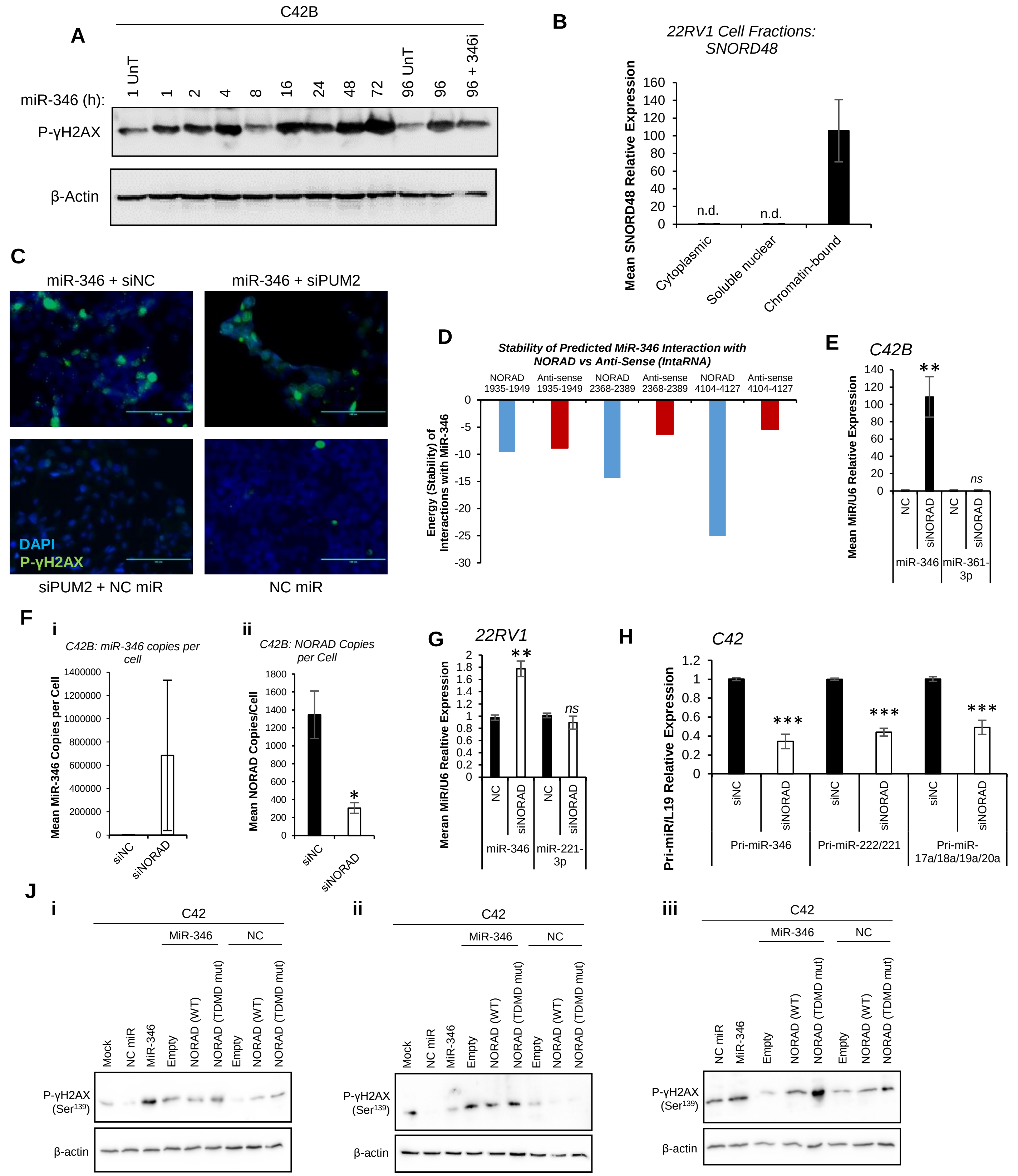

Figure S12: MiR-346 Induces Rapid DNA Damage that is in part NORAD-Independent, NORAD Promotes MiR-346 Target-Directed MiR Decay. *A)* Western blot assay analysis of phospho-γH2AX protein levels (DNA damage marker) in LNCaP cells transfected with 20nM miR-346 for 1-96h. β-actin was used as a loading control. *B)* qRT-PCR analysis of SNORD48 levels in cell fractions of 22RV1 cells. *C)* Immunofluorescence microscopy analysis of phospho-γH2AX protein in C42 cells transfected with NC or miR-346 mimic (10nM) ± siPUM2 (20nM). Scale bars = 100µm. Representative images shown. *D)* Predicted stability of interaction between miR-346 and indicated NORAD regions (IntaRNA: http://rna.informatik.uni-freiburg.de/IntaRNA/Input.jsp), *E)* qRT-PCR analysis of miR-346 and -361-3p levels in C42B cells transfected with siNORAD or siNC for 72h. MiR levels were normalised to U6. *F)* Estimated copies of *(i)* miR-346 and *(ii)* NORAD per C42B cell following transfection with siNORAD or siNC for 72h. Ten-fold serial dilutions of a miR-346 mimic and NORAD qPCR amplicon were prepared, reverse transcribed and analysed by qRT-PCR in parallel to siRNA-transfected C42B samples. *G)* qRT-PCR analysis of miR-346 and -221-3p levels in 22RV1 cells transfected with siNORAD or siNC for 72h. MiR levels were normalised to U6. *H)* qRT-PCR analysis of pri-miR-346, -222/221 and 17a/18a/19a/20a levels in C42 cells transfected with siNORAD or siNC for 72h. Pri-miR levels were normalised to L19. *J)* Western blot analysis of phospho-Ser^{139}-γH2AX protein levels in C42 cells transfected with miR-346 (10nM) ± WT or TDMD-mutant NORAD. β-actin was used as a loading control. Data from three independent repeats relating to Fig 6J are shown. *B, E-H) Columns:* mean ± SEM for three independent experiments, n.d. = not detected. Data relating to **Fig 5** are shown.

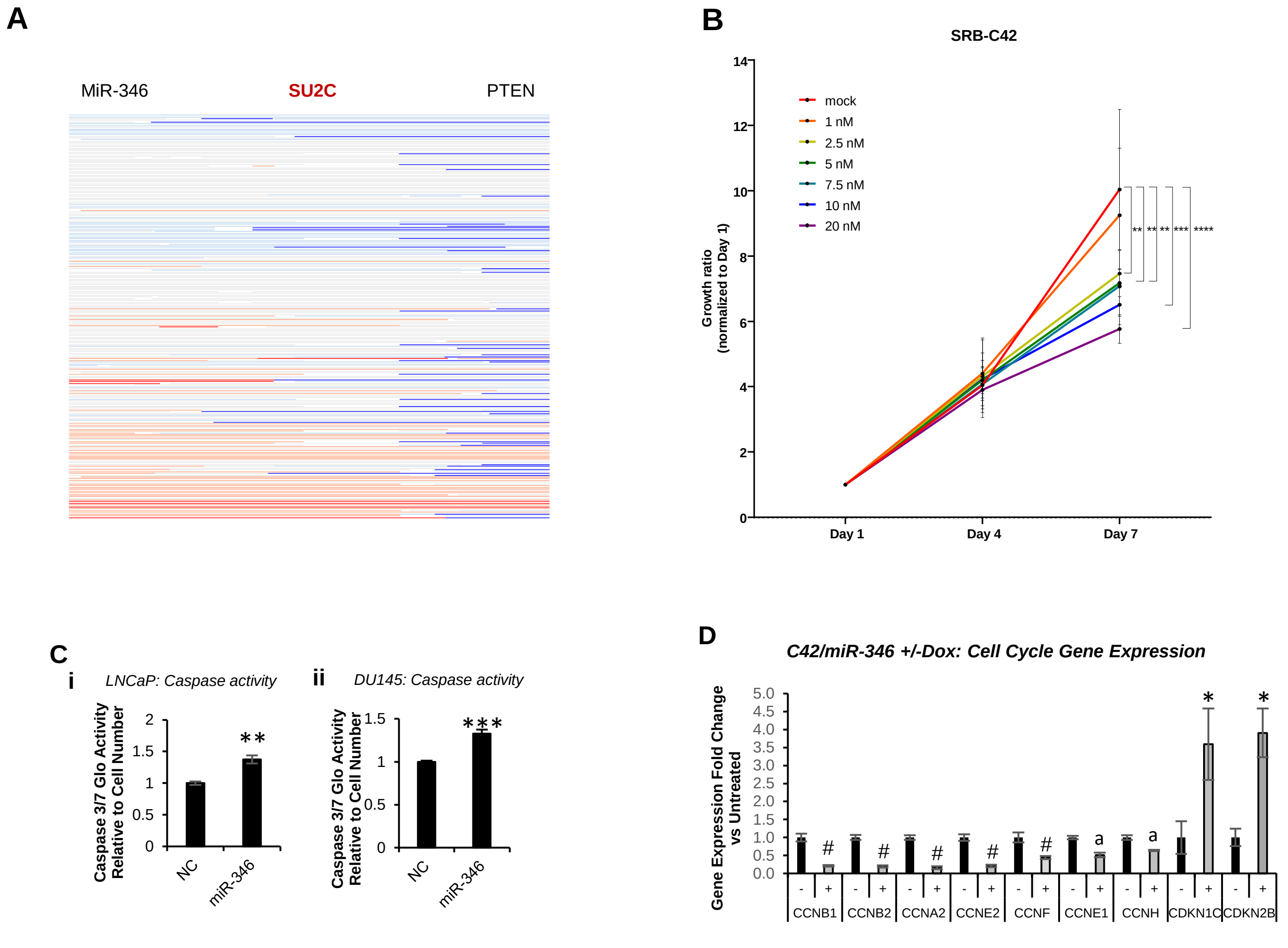

Figure S13: MiR-346 Reduces PC Cell Proliferation and Inhibits Expression of Cell Cycle Genes. *A)* Copy number profile for the region of chromosome ten covering *MIR346* and *PTEN* genes. Each line represent a copy number alteration (CNA) profile from a patient. Blue = loss, grey = no change, red = gain. Breakpoints are observed between *MIR346* and *PTEN*. *B)* SRB assay analysis of C42 cell proliferation following transfection of cells with indicated doses for 6d. Data are presented relative to absorbance at day 0. Points: mean absorbance at 492 nm for three independent experiments performed in quadruplicate ± SEM. *C)* Caspase 3/7 Glo assay analysis of apoptosis in LNCaP *(i)* and DU145 *(ii)* cells transfected with 20nM NC miR or miR-346 for 72h. Caspase activity is shown relative to cell number (quantified by SRB assay) and mean of three independent experiments performed in quadruplicate is shown. *D)* RNA-seq analysis of cell cycle gene expression in C42/miR-346 cels ± Dox (72h). * $P \le 0.05$, a $P \le 0.005$, # $P \le 0.0001$. Data relating to **Fig 6** are shown.

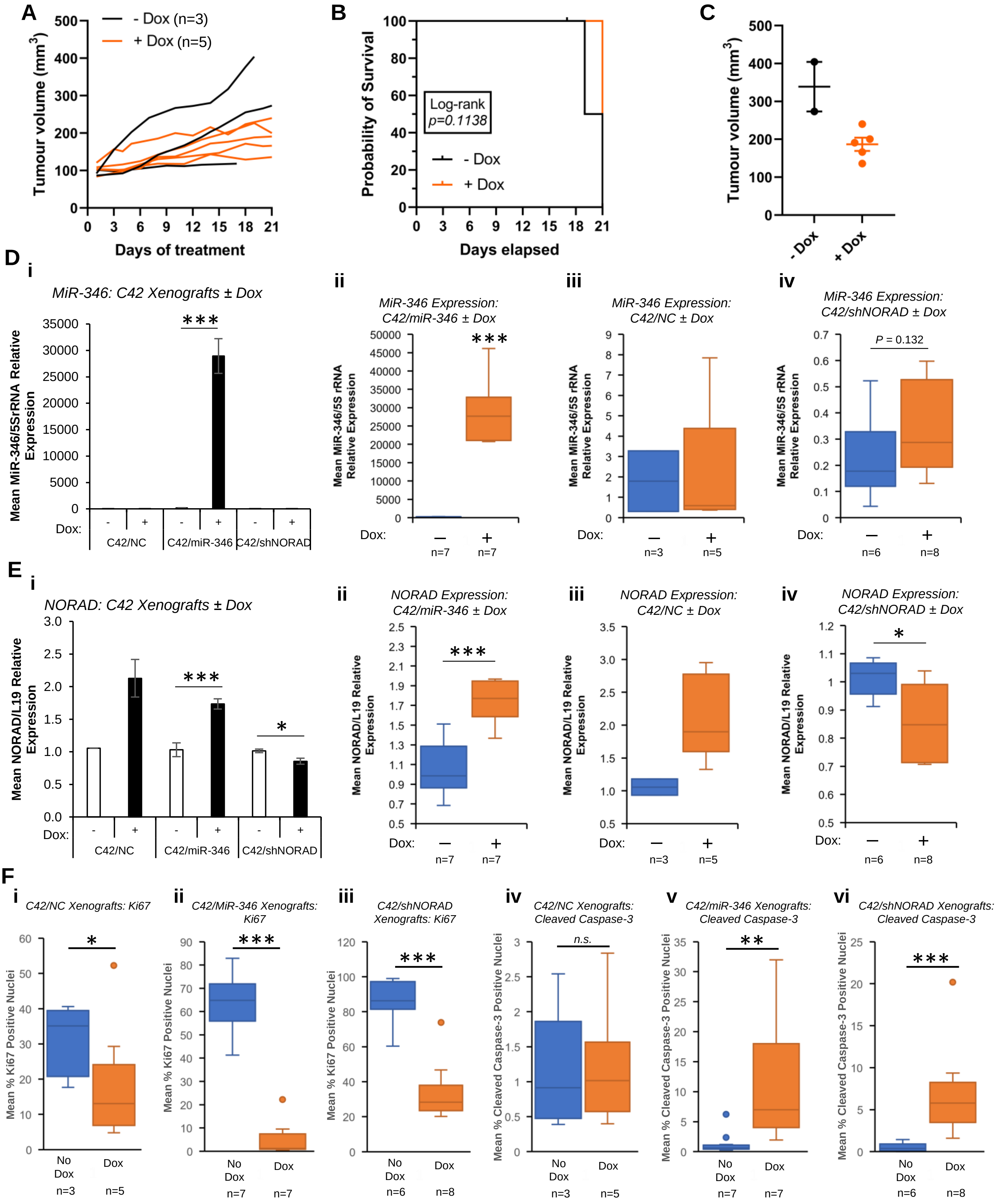

Fig S14: Doxycycline Induction of MiR-346 and shNORAD Significantly Modulates Proliferation and Apoptosis in C42 Xenograft Tumours. *A-C)* 4x10^6 C42 cells stably-expressing doxycycline-inducible RFP-tagged negative control miR (NC-miR) were injected subcutaneously into the flanks of seven NSG mice and allowed to grow to 80mm3, at which point mice were randomly assigned to dox treatment (250ng/ml n=5) or vehicle (n=3) via drinking water. Tumour volume *(A)* was measured daily, and survival *(B)* and final tumour volume *(C)* measured at experimental endpoint (21d). *D,E)* qRT-PCR analysis of miR-346 *(D)* and NORAD *(E)* expression in C42/NC, C42/miR-346 and C42/shNORAD xenografts treated ± Dox at experiment endpoint. *(F)* ImageJ IHCProfiler quantification of IHC-assessed Ki67 *(i-iii)* and cleaved Caspase-3 *(iv-vi)* protein levels in FFPE sections of C42/NC, C42/miR-346 and C42/shNORAD xenografts treated ±Dox. Three fields per xenograft were quantified. Data are shown relative to non-dox treated control xenografts and numbers per group indicated. * $P \leq 0.05$, ** $P \leq 0.005$, *** $P \leq 0.0001$, n.s. = not significant. Data relating to **Fig 6** are shown.

C42/NC

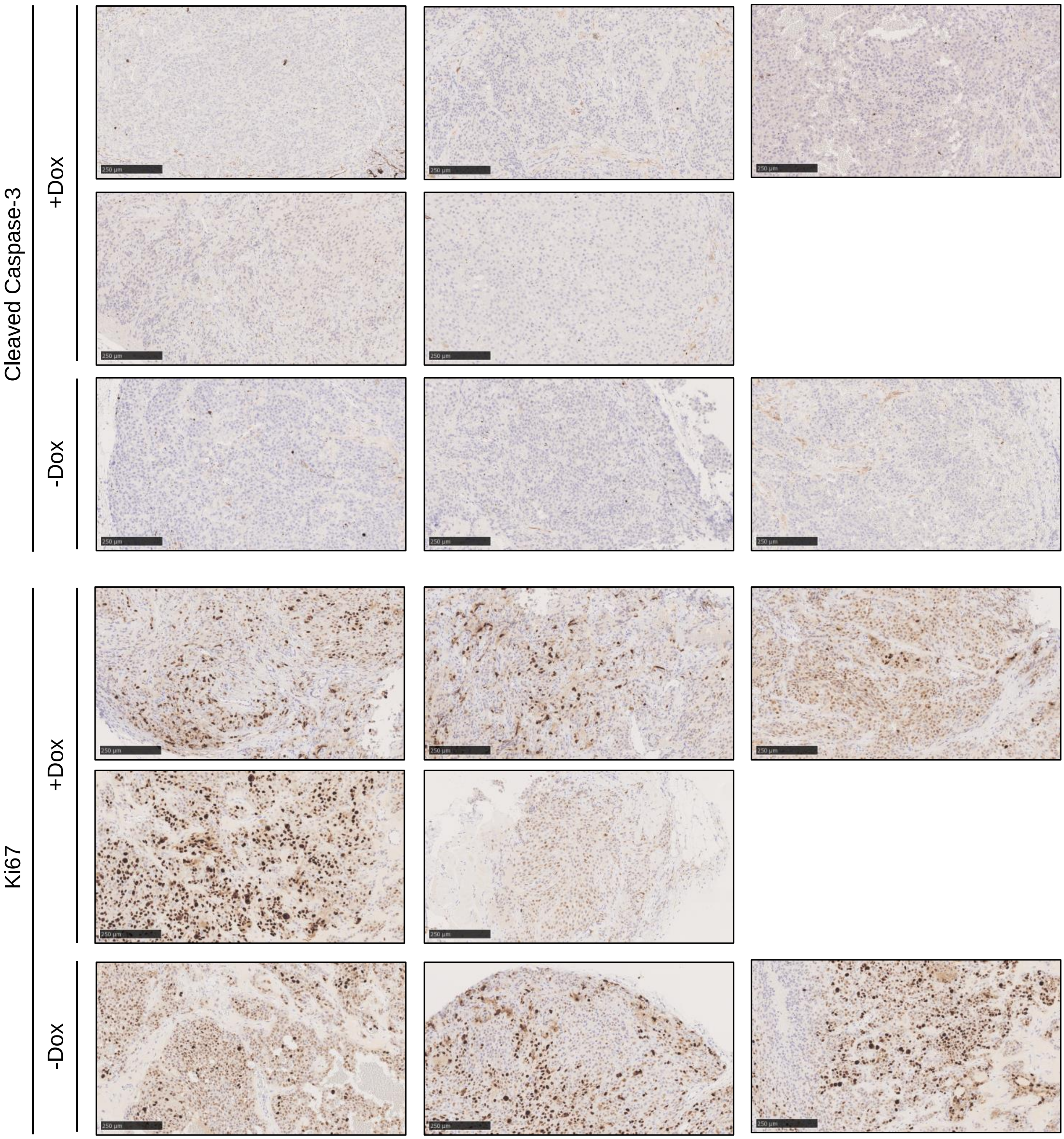

Figure S15: Effect of Negative Control MiR Expression on Protein Levels of Apoptotic (Cleaved Caspase-3) and Proliferative (Ki67) Markers in C42 Xenografts. Immunohistochemistry analysis of cleaved Caspase-3 and Ki67 protein levels in FFPE sections of Dox-treated C42/NC xenograft tumours from NSG mice. Scale bar = 250µm. Data relating to **Fig 6** are shown.

C42/miR-346

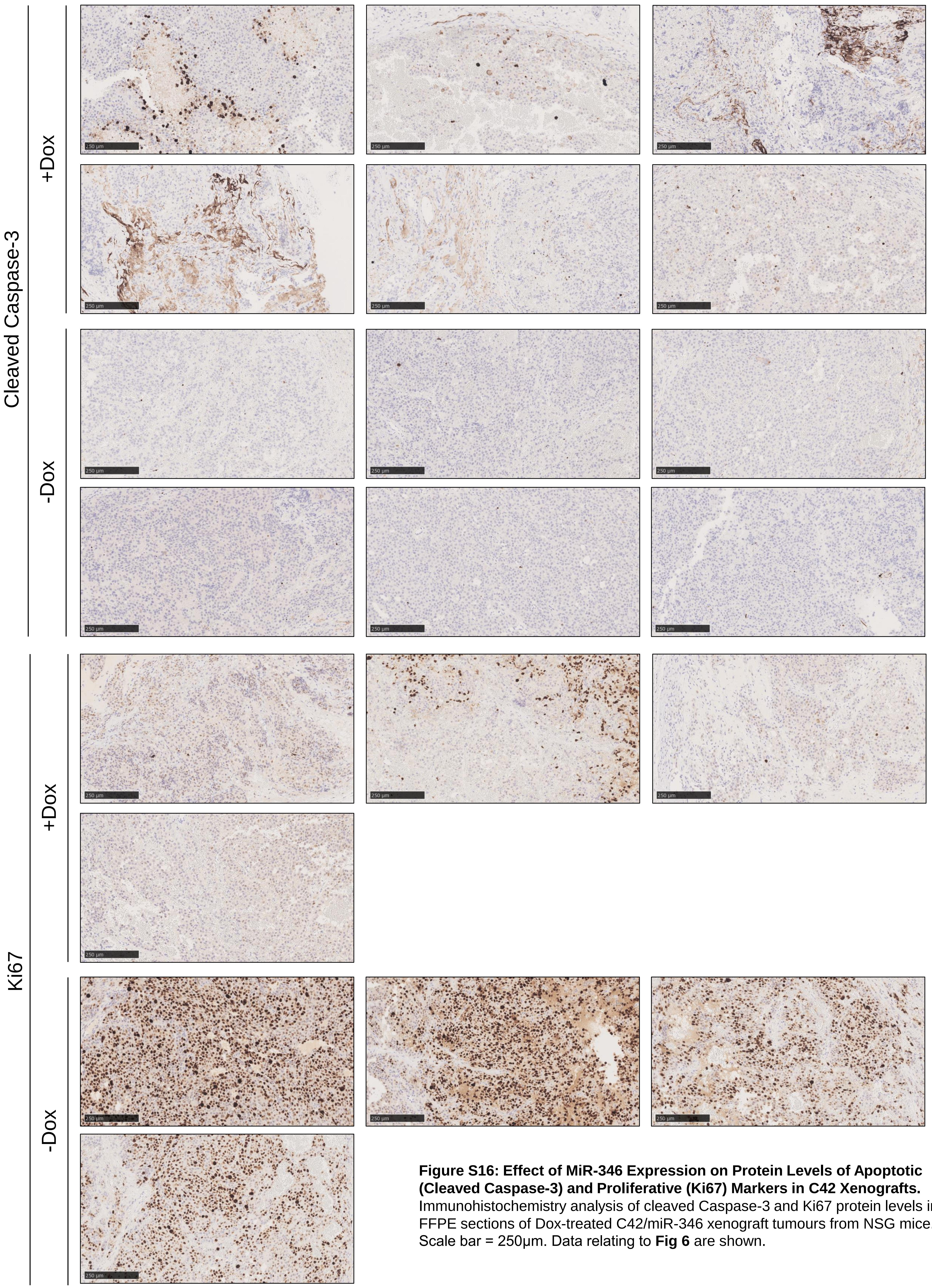

Figure S16: Effect of MiR-346 Expression on Protein Levels of Apoptotic (Cleaved Caspase-3) and Proliferative (Ki67) Markers in C42 Xenografts. Immunohistochemistry analysis of cleaved Caspase-3 and Ki67 protein levels in FFPE sections of Dox-treated C42/miR-346 xenograft tumours from NSG mice. Scale bar = 250µm. Data relating to **Fig 6** are shown.

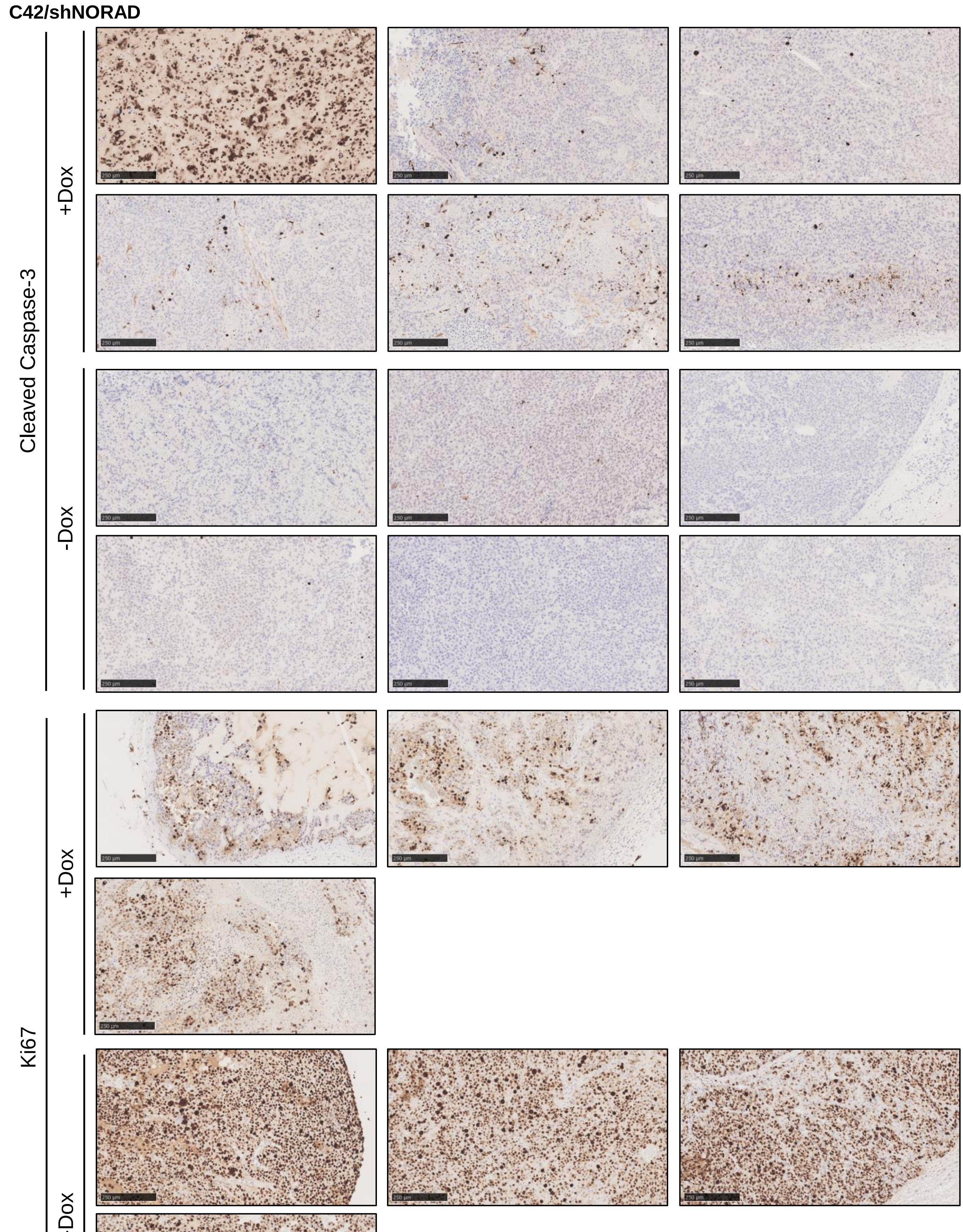

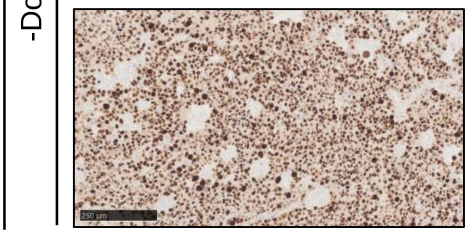

Figure S17: Effect of shNORAD Expression on Protein Levels of Apoptotic (Cleaved Caspase-3) and Proliferative (Ki67) Markers in C42 Xenografts. Immunohistochemistry analysis of cleaved Caspase-3 and Ki67 protein levels in FFPE sections of Dox-treated C42/shNORAD xenograft tumours from NSG mice. Scale bar = 250µm. Data relating to **Fig 6** are shown.
