# Supplementary material for: A non-coding RNA balancing act: miR-346-induced DNA damage is limited by the long non-coding RNA NORAD in prostate cancer
Source: Mol Cancer. 2022 Mar 22;21:82. doi: 10.1186/s12943-022-01540-w (PMC8939142; doi:10.1186/s12943-022-01540-w)
Supplement: Supplementary file 2 — Additional file 2. [file 12943_2022_1540_MOESM2_ESM.pdf]

Gene Location	Strand	Gene ID	Gene Type	Seed Start	Seed End	Total Sites	No of PC lines (/5)	Cell Lines
NC_RNA	-	RP6-99M1.2	lincRNA	45605615	45605622	14	5	DU145, PC3, 22RV1, LAPC4, LNCaP
Intron	-	Intron	Intron	133013410	133013418	13	5	DU145, PC3, 22RV1, LAPC4, LNCaP
CDS	+	MYL6	protein_coding	56553804	56553811	9	5	DU145, PC3, 22RV1, LAPC4, LNCaP
3_UTR	-	PPP2CA	protein_coding	133532948	133532955	8	5	DU145, PC3, 22RV1, LAPC4, LNCaP
3_UTR	-	TUBA1B	protein_coding	49525117	49525124	7	5	DU145, PC3, 22RV1, LAPC4, LNCaP
CDS	-	TRAPPC1	protein_coding	7835098	7835105	5	5	DU145, PC3, 22RV1, LAPC4, LNCaP
3_UTR	-	HNRNPK	protein_coding	86584688	86584695	13	4	PC3, LNCaP, 22RV1, LAPC4
3_UTR	-	YWHAZ	protein_coding	101932896	101932904	11	4	PC3, LNCaP, 22RV1, LAPC4
Promoter	-	YWHAZP2	Promoter	127314982	127314990	10	4	PC3, LNCaP, 22RV1, LAPC4
CDS	-	TMED4	protein_coding	44621091	44621098	9	4	PC3, LNCaP, 22RV1, LAPC4
3_UTR	+	NFIC	protein_coding	3465087	3465094	8	4	DU145, PC3, 22RV1, LAPC4
CDS	+	HIST2H2AC	protein_coding	149858916	149858923	8	4	LNCaP, 22RV1, LAPC4, PC3
3_UTR	-	F11R	protein_coding	160966039	160966046	7	4	PC3, LNCaP, 22RV1, LAPC4
NC_RNA	-	LINC00657	lincRNA	34635627	34635634	7	4	LNCaP, 22RV1, LAPC4, PC3
3_UTR	-	U2AF1	protein_coding	44513184	44513191	7	4	LNCaP, 22RV1, LAPC4, PC3
NC_RNA	+	EEF1A1P5	pseudogene	135895263	135895270	7	4	DU145, PC3, 22RV1, LAPC4
3_UTR	-	ENO1	protein_coding	8938683	8938690	6	4	DU145, PC3, 22RV1, LAPC4
3_UTR	-	EIF4G2	protein_coding	10825904	10825911	6	4	DU145, PC3, 22RV1, LAPC4
NC_RNA	+	SNORA81	snoRNA	186504493	186504500	6	4	DU145, PC3, 22RV1, LAPC4
CDS	+	ABT1	protein_coding	26598952	26598960	6	4	LNCaP, 22RV1, LAPC4, PC3
3_UTR	-	YWHAZ	protein_coding	101931200	101931208	5	4	DU145, PC3, 22RV1, LAPC4
CDS	-	AARS	protein_coding	70316576	70316583	5	4	DU145, PC3, 22RV1, LAPC4
Intergenic	-	Intergenic	Intergenic	127354083	127354090	5	4	LNCaP, 22RV1, LAPC4, PC3
3_UTR	+	PTP4A1	protein_coding	64286424	64286431	5	4	DU145, PC3, 22RV1, LAPC4

Table S1: Top MiR-346-Bound Transcripts in Prostate Cancer. AGO-PAR-CLIP-seq data[49] was interrogated to identify miR-346-associated RNAs. Data is ranked by number of PC cell lines the interaction was identified in. MiR binding site location, strand, gene name, seed coordinates, total number of miR binding sites and number of cell lines positive for miR:RNA interaction are shown. MiR-346 interaction with lncRNA, NORAD (non-coding RNA activated by DNA damage – LINC00657) is highlighted (orange). **See also Fig 2.**

NAME	SIZE	ES	NES	NOM p-val	FDR q-val	FWER p-val	RANK AT MAX	LEADING EDGE
CYTOKINETIC_PROCESS(GO:0032506)	38	-0.65982	-1.7651	0	0.05	0	1894	tags=29%, list=3%, signal=30%
NUCLEAR_ENVELOPE_DISASSEMBLY(GO:0051081)	16	-0.78057	-1.72252	0	0.075	0.05	558	tags=44%, list=1%, signal=44%
MEMBRANE_DISASSEMBLY(GO:0030397)	16	-0.78057	-1.72252	0	0.05	0.05	558	tags=44%, list=1%, signal=44%
OOCYTE_MATURATION(GO:0001556)	27	-0.73952	-1.69974	0	0.05	0.05	2361	tags=30%, list=4%, signal=31%
POSITIVE_REGULATION_OF_CYTOKINESIS(GO:0032467)	36	-0.74824	-1.67278	0	0.05	0.05	1311	tags=31%, list=2%, signal=31%
REGULATION_OF_CYTOKINESIS(GO:0032465)	83	-0.67523	-1.64977	0	0.057135	0.096	1559	tags=29%, list=3%, signal=30%
REGULATION_OF_MEIOTIC_CELL_CYCLE(GO:0051445)	51	-0.66274	-1.62611	0	0.066536	0.129	2603	tags=27%, list=4%, signal=29%
REGULATION_OF_UBIQUITIN-PROTEIN_TRANSFERASE_ACTIVITY(GO:0051438)	55	-0.6143	-1.61197	0	0.067799	0.179	6010	tags=44%, list=10%, signal=49%
G2_DNA_DAMAGE_CHECKPOINT(GO:0031572)	32	-0.70214	-1.60465	0	0.068157	0.222	3219	tags=41%, list=5%, signal=43%
INTERCELLULAR_BRIDGE(GO:0045171)	58	-0.62295	-1.58485	0	0.072129	0.222	3286	tags=29%, list=6%, signal=31%
MITOTIC_G1/S_TRANSITION_CHECKPOINT(GO:0044819)	63	-0.64488	-1.58295	0	0.074663	0.222	6663	tags=48%, list=11%, signal=54%
MITOTIC_G1_DNA_DAMAGE_CHECKPOINT(GO:0031571)	63	-0.64488	-1.58295	0	0.068441	0.222	6663	tags=48%, list=11%, signal=54%
CYCLIN-DEPENDENT_PROTEIN_KINASE_HOLOENZYME_COMPLEX(GO:0000307)	45	-0.69715	-1.58105	0	0.067022	0.222	4923	tags=40%, list=8%, signal=44%
NEGATIVE_REGULATION_OF_CELLULAR_SENESCENCE(GO:2000773)	20	-0.61794	-1.58081	0	0.067276	0.268	1251	tags=20%, list=2%, signal=20%
G1_DNA_DAMAGE_CHECKPOINT(GO:0044783)	64	-0.641	-1.57676	0	0.069673	0.325	6663	tags=47%, list=11%, signal=53%
REGULATION_OF_CELL_CYCLE_ARREST(GO:0071156)	107	-0.57351	-1.57275	0	0.073564	0.419	5578	tags=34%, list=9%, signal=37%
REGULATION_OF_CHROMATIN_SILENCING(GO:0031935)	30	-0.68313	-1.57268	0	0.072178	0.419	4772	tags=40%, list=8%, signal=44%
MITOTIC_CYTOKINESIS(GO:0000281)	84	-0.5612	-1.57267	0	0.070946	0.419	1937	tags=25%, list=3%, signal=26%
TELOMERE_MAINTENANCE_VIA_TELOMERE_TRIMMING(GO:0090737)	15	-0.73926	-1.57113	0	0.075106	0.419	352	tags=27%, list=1%, signal=27%
FORMATION_OF_EXTRACHROMOSOMAL_CIRCULAR_DNA(GO:0001325)	15	-0.73926	-1.57113	0	0.071351	0.419	352	tags=27%, list=1%, signal=27%
T-CIRCLE_FORMATION(GO:0090656)	15	-0.73926	-1.57113	0	0.067953	0.419	352	tags=27%, list=1%, signal=27%
DNA_DAMAGE_RESPONSE,_SIGNAL_TRANSDUCTION_BY_P53_CLASS_MEDIATOR_RESULTING_IN_CELL_CYCLE_ARREST(GO:0006977)	56	-0.66459	-1.56838	0	0.068104	0.463	6663	tags=48%, list=11%, signal=54%

Table S2: Gene Set Enrichment Analysis of MiR-346-Regulated Transcripts (RNA-seq)

GO biological process complete	Fold Enrich	FDR
DNA ligation involved in DNA repair (GO:0051103)	22.36	0.0137
DNA ligation (GO:0006266)	15.97	0.0281
DNA strand elongation involved in DNA replication (GO:0006271)	15.53	0.00855
somatic hypermutation of immunoglobulin genes (GO:0016446)	13.15	0.0419
somatic diversification of immune receptors via somatic mutation (GO:0002566)	12.42	0.049
DNA strand elongation (GO:0022616)	12.15	0.017
regulation of axon extension involved in axon guidance (GO:0048841)	10.82	0.00861
tRNA-containing ribonucleoprotein complex export from nucleus (GO:0071431)	9.86	0.0116
tRNA export from nucleus (GO:0006409)	9.86	0.0115
semaphorin-plexin signaling pathway (GO:0071526)	9.06	0.0157
axon extension (GO:0048675)	9.06	0.0156
tRNA transport (GO:0051031)	9.06	0.0154
ncRNA export from nucleus (GO:0097064)	8.83	0.017
negative regulation of G0 to G1 transition (GO:0070317)	8.6	0.0183
regulation of axon guidance (GO:1902667)	7.8	0.0261
regulation of G0 to G1 transition (GO:0070316)	7.8	0.0259
intracellular transport of virus (GO:0075733)	7.25	0.0157
substrate adhesion-dependent cell spreading (GO:0034446)	6.99	0.0372
transport of virus (GO:0046794)	6.87	0.0183

Table S3: Gene Ontology Pathway Analysis of Shared MiR-346 RNA-seq Dysregulated Genes and AGO-PAR-CLIP-seq-Identified MiR-346-Bound Transcripts in PC

NAME	SIZE	ES	NES	NOM p-val	FDR q-val	FWER p-val	RANK AT MAX	LEADING EDGE
HISTONE_H3-K27_METHYLATION(GO:0070734)	19	-0.60831	-1.52074	0	1	0.717	3958	tags=32%, list=7%, signal=34%
NUCLEAR_HETEROCHROMATIN(GO:0005720)	35	-0.60923	-1.49286	0	1	0.815	2992	tags=34%, list=5%, signal=36%
NUCLEOSOME(GO:0000786)	109	-0.73902	-1.47471	0	1	0.87	4179	tags=47%, list=7%, signal=50%
VITAMIN_TRANSMEMBRANE_TRANSPORT(GO:0035461)	16	-0.73244	-1.47117	0	1	0.87	1767	tags=31%, list=3%, signal=32%
NUCLEOSOME_POSITIONING(GO:0016584)	15	-0.6881	-1.45928	0	1	0.87	100	tags=20%, list=0%, signal=20%
NUCLEOSOMAL_DNA_BINDING(GO:0031492)	55	-0.6423	-1.45259	0	1	1	4017	tags=33%, list=7%, signal=35%
PERICENTRIC_HETEROCHROMATIN(GO:0005721)	22	-0.60704	-1.45208	0	0.969806	1	2580	tags=45%, list=4%, signal=48%
NUCLEAR_NUCLEOSOME(GO:0000788)	38	-0.77223	-1.45174	0	0.866249	1	2501	tags=42%, list=4%, signal=44%
FEMALE_MEIOTIC_NUCLEAR_DIVISION(GO:0007143)	28	-0.65713	-1.45023	0	0.7951	1	4915	tags=36%, list=8%, signal=39%
DNA_PACKAGING_COMPLEX(GO:0044815)	117	-0.7382	-1.44225	0	0.84191	1	4634	tags=50%, list=8%, signal=54%
ANTIMICROBIAL_HUMORAL_IMMUNE_RESPONSE_MEDIATED_BY_ANTIMICROBIAL_PEPTIDE(GO:0061844)	74	-0.68728	-1.44014	0	0.796965	1	5993	tags=22%, list=10%, signal=24%
CENTROSOME_SEPARATION(GO:0051299)	15	-0.77739	-1.43489	0.182524	0.829338	1	2819	tags=47%, list=5%, signal=49%
PORE_COMPLEX(GO:0046930)	18	-0.67416	-1.43107	0	0.842469	1	7189	tags=39%, list=12%, signal=44%
ENDOCYTIC_VESICLE_LUMEN(GO:0071682)	19	-0.67819	-1.43058	0	0.789385	1	9284	tags=37%, list=16%, signal=44%
PROTEIN-DNA_COMPLEX(GO:0032993)	203	-0.60792	-1.42513	0	0.832492	1	3928	tags=37%, list=7%, signal=39%
MITOCHONDRIAL_ATP_SYNTHESIS_COUPLED_PROTON_TRANSPORT(GO:0042776)	17	-0.75783	-1.42459	0	0.788166	1	8783	tags=76%, list=15%, signal=90%
NUCLEOBASE_METABOLIC_PROCESS(GO:0009112)	34	-0.68836	-1.42371	0	0.761359	1	3287	tags=47%, list=6%, signal=50%
PURINE_NUCLEOBASE_METABOLIC_PROCESS(GO:0006144)	20	-0.85652	-1.42007	0	0.780508	1	3287	tags=60%, list=6%, signal=64%
SMALL_NUCLEOLAR_RIBONUCLEOPROTEIN_COMPLEX(GO:0005732)	22	-0.71121	-1.41385	0.17154	0.80417	1	8509	tags=64%, list=14%, signal=74%
OUTER_MITOCHONDRIAL_MEMBRANE_PROTEIN_COMPLEX(GO:0098799)	18	-0.72847	-1.4138	0	0.767164	1	8803	tags=56%, list=15%, signal=65%
INTERSTRAND_CROSS-LINK_REPAIR(GO:0036297)	52	-0.69159	-1.41168	0	0.768494	1	7209	tags=52%, list=12%, signal=59%
CHROMATIN_ASSEMBLY(GO:0031497)	163	-0.66497	-1.40775	0	0.786937	1	4186	tags=42%, list=7%, signal=45%

Table S4: Gene Set Enrichment Analysis of shNORAD-Regulated Transcripts (RNA-seq)

GO biological process complete	+/-	Fold Enrich	FDR
somatic diversification of immunoglobulins involved in immune response (GO:0002208)	+	13.89	3.90E-02
somatic recombination of immunoglobulin genes involved in immune response (GO:0002204)	+	13.89	3.88E-02
isotype switching (GO:0045190)	+	13.89	3.85E-02
DNA replication-independent nucleosome assembly (GO:0006336)	+	13.54	1.27E-04
CENP-A containing nucleosome assembly (GO:0034080)	+	13.2	2.68E-03
CENP-A containing chromatin organization (GO:0061641)	+	13.2	2.65E-03
DNA replication-independent nucleosome organization (GO:0034724)	+	13.2	1.45E-04
centromere complex assembly (GO:0034508)	+	12.48	8.41E-04
chromatin remodeling at centromere (GO:0031055)	+	12	3.99E-03
ribosomal large subunit biogenesis (GO:0042273)	+	10.56	2.88E-06
somatic diversification of immune receptors via germline recombination within a single locus (GO:0002562)	+	9.7	3.40E-02
somatic cell DNA recombination (GO:0016444)	+	9.7	3.37E-02
cholesterol biosynthetic process (GO:0006695)	+	9.66	9.74E-03
histone exchange (GO:0043486)	+	9.62	3.15E-03
secondary alcohol biosynthetic process (GO:1902653)	+	9	1.32E-02
mitochondrial translational elongation (GO:0070125)	+	9	1.23E-05
mitochondrial translational termination (GO:0070126)	+	8.9	1.28E-05
translational termination (GO:0006415)	+	8.16	2.91E-05
sterol biosynthetic process (GO:0016126)	+	7.92	2.34E-02
mitotic spindle organization (GO:0007052)	+	7.81	8.29E-08
ribosome assembly (GO:0042255)	+	7.22	1.26E-02
anaphase (GO:0051322)	+	7.19	5.96E-07
mitotic anaphase (GO:0000090)	+	7.19	5.74E-07

Table S5: Gene Ontology Pathway Analysis of Shared MiR-346-, shNORAD- and siNORAD-Dysregulated Genes

GO biological process complete	+/-	Fold Enrichment	FDR
nuclear DNA replication (GO:0033260)	+	19.52	1.25E-20
cell cycle DNA replication (GO:0044786)	+	19.09	1.86E-20
DNA replication initiation (GO:0006270)	+	17.74	4.58E-13
telomere maintenance via semi-conservative replication (GO:0032201)	+	18.07	6.9E-11
DNA strand elongation (GO:0022616)	+	18.39	1.86E-09
CENP-A containing chromatin organization (GO:0061641)	+	15.18	2.18E-09
CENP-A containing nucleosome assembly (GO:0034080)	+	15.18	2.2E-09
DNA strand elongation involved in DNA replication (GO:0006271)	+	21.69	2.66E-09
double-strand break repair via break-induced replication (GO:0000727)	+	26.61	2.06E-07
kinetochore organization (GO:0051383)	+	18.07	3.66E-07
mitotic DNA replication (GO:1902969)	+	23.66	2.5E-06
DNA replication checkpoint (GO:0000076)	+	17.22	2.58E-06
regulation of DNA-directed DNA polymerase activity (GO:1900262)	+	25.3	1.18E-05
positive regulation of DNA-directed DNA polymerase activity (GO:1900264)	+	25.3	1.19E-05
prophase (GO:0051324)	+	17.35	1.25E-05
mitotic prophase (GO:0000088)	+	17.35	1.25E-05
DNA unwinding involved in DNA replication (GO:0006268)	+	17.35	1.26E-05
pre-replicative complex assembly (GO:0036388)	+	27.88	5.65E-05
pre-replicative complex assembly involved in nuclear cell cycle DNA replication (GO:0006267)	+	27.88	5.67E-05
pre-replicative complex assembly involved in cell cycle DNA replication (GO:1902299)	+	27.88	5.69E-05
regulation of attachment of spindle microtubules to kinetochore (GO:0051988)	+	17.52	6.35E-05
positive regulation of protein localization to chromosome, telomeric region (GO:1904816)	+	15.01	0.000649
meiotic chromosome condensation (GO:0010032)	+	23.23	0.000735
DNA replication-dependent nucleosome organization (GO:0034723)	+	16.26	0.00231
DNA replication-dependent nucleosome assembly (GO:0006335)	+	16.26	0.00232
positive regulation of establishment of protein localization to telomere (GO:1904851)	+	16.26	0.00232
mitotic DNA replication checkpoint (GO:0033314)	+	16.26	0.00233
female meiosis chromosome segregation (GO:0016321)	+	21.69	0.00545
G-quadruplex DNA unwinding (GO:0044806)	+	18.59	0.00778
U4 snRNA 3'-end processing (GO:0034475)	+	16.26	0.0108
DNA replication preinitiation complex assembly (GO:0071163)	+	32.53	0.0157
mitotic DNA replication initiation (GO:1902975)	+	24.4	0.0248
positive regulation of protein localization to kinetochore (GO:1905342)	+	24.4	0.0249
regulation of protein localization to kinetochore (GO:1905340)	+	24.4	0.0249
nuclear cell cycle DNA replication initiation (GO:1902315)	+	24.4	0.025
cell cycle DNA replication initiation (GO:1902292)	+	24.4	0.025
regulation of isotype switching to IgA isotypes (GO:0048296)	+	16.26	0.0481
positive regulation of exit from mitosis (GO:0031536)	+	16.26	0.0482
nuclear ncRNA surveillance (GO:0071029)	+	16.26	0.0483
nuclear polyadenylation-dependent tRNA catabolic process (GO:0071038)	+	16.26	0.0484
nuclear polyadenylation-dependent rRNA catabolic process (GO:0071035)	+	16.26	0.0484
nuclear polyadenylation-dependent ncRNA catabolic process (GO:0071046)	+	16.26	0.0485
maintenance of centrosome location (GO:0051661)	+	16.26	0.0486
establishment of sister chromatid cohesion (GO:0034085)	+	16.26	0.0487
regulation of ovarian follicle development (GO:2000354)	+	16.26	0.0487

Table S6: Gene Ontology Pathway Analysis of Shared Genes Dysregulated by MiR-346 and siPUM2

Pathway	*P* value
GCM_BMPR2	1.19E-22
GCM_CALM1	5.31E-20
GCM_RAN	2.80E-18
AGGTGCA_MIR500	3.98E-18
AGTCAGC_MIR345	2.86E-17
GCM_RAB10	3.42E-17
GCM_MAP4K4	4.11E-17
GCM_DEAF1	4.28E-17
CAGGGTC_MIR504	8.91E-17
GCM_PTK2	1.02E-16
GCM_CRKL	1.14E-16
GCM_PTPRD	1.81E-16
SCHAEFFER_PROSTATE_DEVELOPMENT_AND_CANCER_BOX4_DN	2.96E-16
GABRIELY_MIR21_TARGETS	3.19E-16
GACAATC_MIR219	4.48E-16
TGCCTTA_MIR124A	6.17E-16
TERAO_AOX4_TARGETS_HG_UP	6.21E-16
AGCACTT_MIR93_MIR302A_MIR302B_MIR302C_MIR302D_MIR372_MIR373_MIR520E_MIR520A_MIR526B_MIR520B_MIR520C_MIR520D	7.68E-16
GCM_MAP1B	7.82E-16
GCACTTT_MIR175P_MIR20A_MIR106A_MIR106B_MIR20B_MIR519D	8.18E-16
ACCATTT_MIR522	1.31E-15
GTGCCTT_MIR506	2.06E-15
GGCACTT_MIR519E	2.89E-15
GCM_CSNK1D	3.37E-15
ACTGCCT_MIR34B	6.00E-15
GO_PROTEIN_LOCALIZATION_TO_GOLGI_APPARATUS	6.72E-15
GCM_SUFU	8.45E-15
RODRIGUES_THYROID_CARCINOMA_POORLY_DIFFERENTIATED_DN	1.04E-14
TTTGCAG_MIR518A2	1.05E-14
CAGCACT_MIR5123P	1.61E-14
GCM_IL6ST	1.66E-14
ACACTGG_MIR199A_MIR199B	1.88E-14
TTGGGAG_MIR150	1.93E-14
TGCTGCT_MIR15A_MIR16_MIR15B_MIR195_MIR424_MIR497	2.04E-14
GTTTGTT_MIR495	2.35E-14
GCM_UBE2N	2.62E-14
GSE19888_ADENOSINE_A3R_ACT_VS_TCELL_MEMBRANES_ACT_IN_MAST_CELL_DN	2.82E-14
GO_ESTABLISHMENT_OF_PROTEIN_LOCALIZATION_TO_GOLGI	2.95E-14
GCTGAGT_MIR5125P	3.91E-14
CACTGTG_MIR128A_MIR128B	4.27E-14

Table S7: Top NORAD-Correlated Pathways in mCRPC Patient Tumours – SU2C Data Set[51]
