# Supplementary material for: A non-coding RNA balancing act: miR-346-induced DNA damage is limited by the long non-coding RNA NORAD in prostate cancer
Source: Mol Cancer. 2022 Mar 22;21:82. doi: 10.1186/s12943-022-01540-w (PMC8939142; doi:10.1186/s12943-022-01540-w)
Supplement: Supplementary file 3 — Additional file 3. [file 12943_2022_1540_MOESM3_ESM.docx]

**Supplementary Methods**

**DNA fibre assay**

48h post-miR-346 transfection, cells were pulsed with media containing 5µM 5-Bromo-2’-deoxyuridine (BrdU, B5002, Sigma-Aldrich) for 20 minutes, washed three times with PBS followed by incubation with medium containing 200µM 5-iodo-2’-deoxyuridine (IdU, I7125, Sigma-Aldrich) for 20 minutes. Cells were washed x2 in ice-cold PBS, trypsinized, and resuspended to 1X10^6^ cells/ml in ice-cold PBS. 2µl cell suspension was spotted onto Superfrost Plus slides (Thermo Scientific, UK) and dried for 5-10min, after which 7µl of spreading/lysis buffer (200 mM Tris-HCl pH7.5, 50 mM EDTA, 0.5% SDS) was added to each drop, mixed immediately by gentle stirring, and incubated for 2-3mins. Slides were tilted at approximately 10°, such that drops run the complete slide length in 5min. Spreads were air-dried (30min), fixed with methanol:acetic acid (3:1) for 10mins and air-dried for 30min. Slides were washed 2x 5min with H_2_0 and 1x 5min with 2.5M HCl before denaturing with 2.5M HCl (1h). Slides were washed x2 with PBS and 2x 5min with blocking buffer (PBS, 1% BSA, 0.1% Tween-20) before 1h incubation with blocking buffer. Slides were incubated with rat anti-BrdU antibody (Abcam, ab6326, 1:500) in blocking buffer overnight at 4^°^C, washed x3 with PBS, fixed with 1% formalin (10min), washed x3 with PBS and quenched with 500mM glycine pH8.0 (10min). Slides were then washed x3 with PBS and 3x 5min with blocking buffer and incubated with mouse anti-BrdU (BD biosciences, B44 - BrdU is saturated by previous antibody and this antibody has better affinity for IdU) 1:100 in blocking buffer overnight at 4^°^C. Slides were washed x2 in PBS and 3x 5min with blocking buffer prior to incubating with secondary antibodies: Cy3-conjugated goat anti-rat (Abcam ab98416) and Cy2-conjugated goat anti-mouse (Abcam ab6944), both 1:500 in blocking buffer for 2h. Slides were washed x2 in PBS, 3x 5min in blocking buffer, x2 in PBS and mounted in glycerol:PBS (1:1). Images were acquired using LSM510 confocal microscope (Zeiss) with 60X objective. Fibres were quantified, and replication fork speed calculated using ImageJ as described^[1]^. 5HU (1mM, for 40min during nucleotide pulses), was used as control for replication fork stalling.

**RNA-seq**

RNA integrity and quantitation were assessed using the RNA Nano 600 Assay Kit on the Bioanalyser 2100 system (Agilent Technologies). 1µg total RNA per sample was used as input and sequencing libraries prepared using NEBNext Ultra RNA Library Prep Kit for Illumina (NEB, USA) according to the manufacturer’s instructions. Library quality was assessed sequenced using the Ilumina Bioanalyser 2100 system (Agilent Technologies). Clustering of index-coded samples was performed on cBot Cluster Generation System using PE Cluster Kit cBot-HS (Illumina) according to the manufacturer’s instructions. Libraries were sequenced on Illumina platform and paired-end reads generated. Clean reads were obtained from raw reads in FASTQ format through removal of adapters, poly-N sequences and low-quality reads. Paired end clean reads were mapped to reference genome (GRCh38) using HISAT2. Featurecounts was used to count numbers of reads mapped for each gene, and RPKM calculated. Differential expression analysis was performed using DESeq2 R package. Resultant *P* values were False Discovery Rate (FDR)-adjusted using the Benjamini and Hochberg’s approach. Gene Ontology (GO) and Kyoto Encyclopedia of Genes and Genomes (KEGG) Pathway enrichment analyses were performed using the clusterProfiler R package.

**NORAD RNA *In Situ* Hybridisation (RNA-ISH)**

RNA in situ hybridization (ISH) detection of NORAD was performed on freshly-cut 4µm sections derived from FFPE blocks with probe for NORAD (Cat. No. 525638, Advanced Cell Diagnostics, Hayward, CA, USA), using the RNAscope 2.5 LS Reagent Kit-BROWN (Cat. No. 322100, Advanced Cell Diagnostics, Hayward, CA, USA) on a BOND RX platform (Leica, Nussloch, Germany) according to the manufacturer's protocol. A housekeeping gene peptidylprolyl isomerase B (cyclophilin B - PPIB) probe (Cat. No. 313908, Advanced Cell Diagnostics, Hayward, CA, USA) was used as an internal-control for mRNA quality per sample. Sections were scanned at 40x on a VS200 Research Slide Scanner (Olympus, Tokyo, Japan) and analyzed using the RNAish analysis module from HALO Image analysis software (Indica Labs, USA). Areas of tissue with a PPIB expression of less than 4 spots/cell were excluded from the analysis.

**Immunohistochemistry**

Heat mediated antigen retrieval was performed in EDTA based pH 9.0 solution, and then the endogenous peroxidase was quenched with 3% hydrogen peroxide. For Ki67 staining, the sections were incubated with mouse monoclonal to Ki67 (clone K2, Leica, PA0230), followed by rabbit anti mouse IgG (Leica Bond Polymer Refine Detection, DS9800). For cleaved caspase3 staining, the sections were incubated with rabbit polyclonal to cleaved caspase3 (Cell Signaling, 9661). The sections were subsequently incubated with anti-rabbit IgG conjugated with polymeric horseradish peroxidase linker (Leica Bond Polymer Refine Detection, DS9800).  DAB was used as the chromogen and the sections were then counterstained with hematoxylin and mounted with DPX. IHC was performed on Leica BOND RX. Images of stained xenograft sections were obtained using a [NanoZoomer-SQ Digital slide scanner](https://nanozoomer.hamamatsu.com/all/en/scanner/search/C13140-01/index.html) (Hamamatsu, Japan) and analysed using NDP.view2 software. Images at x10 magnification were exported and automated quantification of Ki67 and cleaved Caspase-3 staining performed using IHC Prolifer plug-in for ImageJ (https://sourceforge.net/projects/ihcprofiler/)^[2]^.

**Endogenous PUM2 RNA Immunoprecipitation**

Prior-transfected cells (5x10^6^ per condition) were PBS-washed, pelleted by centrifugation and lysed in 1ml ice-cold RNA-IP lysis buffer (20mM Tris-HCl pH7.5, 150mM NaCl, 1.5mM MgCl_2_, 2mM DTT, 0.5% sodium deoxycholate, 0.5% NP-40, 3μl/ml SUPERaseIn RNase inhibitor (ThermoFisher Scientific, UK), 5μl/ml protease inhibitor cocktail (P8340, Sigma-Aldrich, UK)). Lysates were cleared by centrifugation (13,000rpm, 20min, 4°C) and 5% removed as input. 1mg of total protein lysate was incubated with prior antibody-coupled Protein G Dynabeads, (5μg per sample of Rb α PUM2 [A300-202A, Bethyl Laboratories, TX, USA] or IgG control) at 4°C for 16h with rotation. Lysates were removed and beads washed x3 with ice-cold PBS containing 0.1% Tween20 (TBST) (3x 5min with rotation). Beads and input samples were resuspended in Trizol Reagent (ThermoFisher Scientific, UK) and RNA isolation performed according to the manufacturer’s instructions, prior to reverse transcription and qRT-PCR.

**Bioinformatics analysis – INDUCE-seq data**

INDUCE-seq raw data was processed as described^[3]^, generating .bed files containing break genome locations and break counts. Resultant data was visualised using SeqPlots R package (version 1.27.0)^[4]^. MiR-346-induced DSBs were used as background tracks in the majority of analyses. For analysis of break enrichment within given gene sets, RNA-seq data (from C42 cells, described above) were filtered (FDR < 0.05). Genes with logFC≤-1 (dox-induced *versus* non-induced) was classified as downregulated, whilst those with logFC≥1 were classified as upregulated. Top highly-expressed genes in C42 cells were identified from normalised RNA-seq data by ranking according to FPKM values. MiR-346 DSB counts (BAM format) were plotted against gene set TSSs (TSS ± 5kb). Bin track size was set at 150bp and error bars represent SEM. ChIP-seq datasets of TF binding sites were downloaded as BED files from Gene Expression Ominbus (GEO). The following datasets were accessed: GSM2219854 (AR), GSM1907204 (c-Myc), GSM2827203 (CTCF), GSM1145322 (ETV1), GSM941195 (GATA2), GSM1863005 (FOXA1), GSM2480818 (HOXB13), GSM989640 (NKX3.1), GSM696843 (POLR2A), GSM50151 (PR), GSE43988 (ESR1), GSM759669 (NR3C1), GSM2305255 (EZH2), GSM2305251 (n-Myc), GSM838400 (RUNX2). MiR-346 DSB counts (BAM format) were plotted against TF binding sites coordinates ± 10kb in SeqPlots. Bin track size was set at 200bp and error bars represent SEM. To identify DSB frequency (breaks per 100,000bp) within genes or specifically at TSSs (defined as canonical gene start sites ± 200bp), the following equations were applied:

$$DSBs enrichment at TSSs= \frac{number of DSBs at TSSs}{gene number \times200bp}$$

$$DSBs enrichment at genes= \frac{DSBs count at any gene site}{sum of all gene lengths (bp)}$$

*De novo* motif enrichment analysis was performed using the HOMER v4.11 package, with output motif lengths set at between 10bp and 15bp. Percentage of C42/miR-346 +dox INDUCE-seq DSB reads containing identified motifs (target) were compared against percentage of C42/NC -dox INDUCE-seq DSB reads containing the motif (background). 50bp regions were selected for motif finding and total number of background sequences was limited to 50,000.

**Bioinformatics Analysis – NORAD Activity Score (NAS)**

NAS is defined as summed expression of 33 genes previously identified as downregulated with PUM1/2 overexpression *and* NORAD siRNA and upregulated with NORAD overexpression in U2OS cells (n=17)^[5]^, or downregulated in NORAD^-/-^ HCT116 cells *versus* WT cells and identified as PUM2-interacting transcripts (n=16)^[6]^): ESCO2, ID4, NET1, MASTL, LMNB2, CAV2, KIF20B, SMC1A, SMAD7, BARD1, HMGB1, CENPT, CENPE, SMC4, SMC3, TBX3, MCM8, PARP1, CDK1, CENPF, TTK, EXO1, GSK3B, WDHD1, RBBP8, CDK2, CKAP2, PARP2, SOX2, MCM4, PRC1, ASPM, KIF18A.

**Supplementary Materials**

**Primers for Site Directed Mutagenesis of pcDNA3.1-NORAD**

| **Name** | **Sequence (5’ 🡪 3’)** | **Mutagenesis round** | **TDMD site mutated (nt)** |
| --- | --- | --- | --- |
| TDMD 1a | GGTGTTTGGGGTGGGCCAAGCCGTGGTGGGCAGAGGAGG | 1 | 4103-4126 |
| TDMD 1b | CCTCAGATGATAACCATTGTTATGTGTGTGCAATTTTATTTAACTCTCGTGTCTAACTGGTGGACAAGTTATATGAAATATCTAGT | 1 | 2367-2388 |
| TDMD 2a | GGGGTGGGCCAAGCCGTGCTCGCGTCTGGAGGTATGCAGGGAGAG | 2 | 4103-4126 |
| TDMD 2b | CCATTGTTATGTGTGTGCAATTTTATTTAACTCTCGTGTCTAACTCCTTCTGTAGTTATATGAAATATCTAGTCTTTCTAGATATTTGGAA | 2 | 2367-2388 |

**Template Oligonucleotides for NORAD 1950-2110 PCR Prior to *In Vitro* Transcription**

| **Name** | **Sequence (5’ 🡪 3’)** |
| --- | --- |
| NORAD 1950-2110 WT | AATTGCAAAAGGTAATATTACTAGTGTGTTCATACTTGGACATTTTCAGACACCATTTTTCTATATG  TTTTGTGCATTTTGTTTTGCTCTGTATATAGTATATATAATGGACAAATAGTCCTAATTTTTCAACAT  CTAGTCTCTAGATGTTAAAGAGGTTG |
| NORAD 1950-2110 miR-346 BS mutant | AATTGCAAAAGGTAATATTACTAGTGTGTTCATACTTGGACATTTAGGCGTTTCATTTTTCTATATG  TTTTGTGCATTTTGTTTTGCTCTGTATATAGTATATATAATGGACAAATAGTCCTAATTTTTCAACAT  CTAGTCTCTAGATGTTAAAGAGGTTG |
| NORAD 1950-2110 PRE mutant | AATTGCAAAAGGTAATATTACTAGTGTGTTCATACTTGGACATTTTCAGACACCATTTTTCTATATG  TTTTGTGCATTTTGTTTTGCTCACAGCCAGGTATATATAATGGACAAATAGTCCTAATTTTTCAACA  TCTAGTCTCTAGATGTTAAAGAGGTTG |
| Neg ctrl region | GATAGGATACATCTTGGACATGGAATTGTTAAGCCACCTCTGAGCAGTGTATGTCAGGACTTGTTC  ATTAGGTTGGCAGCAGAGGGGCAGAAGGAATTATACAGGTAGAGATGTATGCAGATGTGTCCATA  TATGTCCATATTTACATTTTGATAGCCATTG |

**Primers for T7 NORAD PCR Prior to *In Vitro* Transcription**

| **Name** | **Sequence (5’ 🡪 3’)** |
| --- | --- |
| NORAD T7 1950-2110 F | TTCTAATACGACTCACTATAGGGAATTGCAAAAGGTAATATTACTAG |
| NORAD T7 1950-2110 R | CAACCTCTTTAACATCTAGAGACTAGATGTTG |
| NC reg T7 F | TTCTAATACGACTCACTATAGGGGATAGGATACATCTTGGACAT |
| NC reg T7 R | CAATGGCTATCAAAATGTAAATATGGACAT |

**Primers for qRT-PCR**

| **Primer Name** | **Sequence (5’ – 3’)** |
| --- | --- |
| AHNAK2F F | GTGCAGAAACGGAAGATGACC |
| AHNAK2F R | GCCTCAGTCGTGTATTCGTAGA |
| ASPM F | AACCCATTATCGCTGTGGCA |
| ASPM R | CCAAGTGAAGCCCTGTTCCT |
| BIRC5 F | AGGACCACCGCATCTCTACAT |
| BIRC5 R | AAGTCTGGCTCGTTCTCAGTG |
| CAV2 F | GCTCCCACAAGGTAAAACTTCA |
| CAV2 R | AGAAAGCTTTGTGTATCGCTCTT |
| CDC25A F | GTGAAGGCGCTATTTGGCG |
| CDC25A R | TGGTTGCTCATAATCACTGCC |
| CDK1 F | AAACTACAGGTCAAGTGGTAGCC |
| CDK1 R | TCCTGCATAAGCACATCCTGA |
| CDK2 F | CGTCCACCTCCTACCCCATA |
| CDK2 R | ACCCATGCCCTCACTCAATC |
| CENPE F | GATTCTGCCATACAAGGCTACAA |
| CENPE R | TGCCCTGGGTATAACTCCCAA |
| CENPF F | CAAGAATATGCACAACGTCCTGC |
| CENPF R | GAACGCCTGTTCAGCTCTG |
| CENPT F | CATCCTACTAACTGCCCCAGA |
| CENPT R | CCGCAACTGCTCTCTTGTC |
| CHEK1 F | ATATGAAGCGTGCCGTAGACT |
| CHEK1 R | TGCCTATGTCTGGCTCTATTCTG |
| CKAP2 F | TACCCGGGACACTGTGAAAC |
| CKAP2 R | CATGAGGCCCTTTCCGGATT |
| CYREN F | AATCCGAGACTAAAACGAGGGT |
| CYREN R | GCCTTCATTGGTGCCACATT |
| DAZAP2 F | AGCAAAGGTCAATATCCAACACA |
| DAZAP2 R | GCCTGAGGAAGATGCAAGGTC |
| ESCO2 F | TGTGTGCAAGTCTTGTGGTATG |
| ESCO2 R | CCCATCCCAAAACTCTGCTACT |
| FEN1 F | ATGACATCAAGAGCTACTTTGGC |
| FEN1 R | GGCGAACAGCAATCAGGAACT |
| GAPDH F | ATGGGGAAGGTGAAGGTCG |
| GAPDH R | GGGGTCATTGATGGCAACAATA |
| GSK3B F | CAGGTGGCTCTTTGTTTGCC |
| GSK3B R | GGCTCATCAGCCTTTGACCT |
| H2AFZ F | GGCGGTAAGGCTGGAAAGG |
| H2AFZ R | TGTCGATGAATACGGCCCAC |
| HIST1H2AB F | ACTCGGTCTTCTCGTGCAG |
| HIST1H2AB R | GCTCCTCGTCATTGCGGAT |
| HMGB1 F | TATGGCAAAAGCGGACAAGG |
| HMGB1 R | CTTCGCACCATCACCAATGGA |
| IFIT1 F | TTGATGACGATGAAATGCCTGA |
| IFIT1 R | CAGGTCACCAGACTCCTCAC |
| IGF1 F | GCTCTTCAGTTCGTGTGTGGA |
| IGF1 R | GCCTCCTTAGATCACAGCTCC |
| IRAK1 F | GCACCCACAACTTCTCGGAG |
| IRAK1 R | CACCGTGTTCCTCATCACCG |
| ITGB4 F | CTCCACCGAGTCAGCCTTC |
| ITGB4 R | CGGGTAGTCCTGTGTCCTGTA |
| KDM3A F | GTGCTCACGCTCGGAGAAA |
| KDM3A R | GTGGGAAACAGCTCGAATGGT |
| KIF18A F | TGGACTTACTTTACACCAGCCC |
| KIF18A R | GCTGTTTTGTCTTGTTGTCGC |
| KIF20B F | GCACCAGAGTGTTGCCTTCA |
| KIF20B R | CACTGACTCGAATTACACGAGAC |
| L19 F | GCGGAAGGGTACAGCCAAT |
| L19 R | GCAGCCGGCGCAAA |
| LIG3 F | TCACTGGCGTGATGTAAGACA |
| LIG3 R | CCTGGAATGATAGAACAGGCTTT |
| MAGED F | GAAGACAGCGCCTTGCTTATG |
| MAGED R | GTGGCCTGGTTAGTAGGTGG |
| MCM8 F | GCCTCCTGGGTTTATTGCAG |
| MCM8 R | TACTTTTGGGACATCATTTTTCAGA |
| MED12L F | TCCAGGACACGGGAAAGAAGA |
| MED12L R | ACTCTGGGATCGAGCAGTAAC |
| NCOA3 F | ACATAAACGCCAGTCCTGAAAT |
| NCOA3 R | CCTTCCTCCATCATAGCTCGT |
| NET1 F | ACACCCGCCAAGAGAAGGA |
| NET1 R | TGTTCACCTCGGGACATTTCATA |
| NORAD F | AGCGAAGTCCCGAACGACGA |
| NORAD R | TGGGCATTTCCAACGGGCCAA |
| PARP1 F | TGGAAAAGTCCCACACTGGTA |
| PARP1 R | AAGCTCAGAGAACCCATCCAC |
| PARP2 F | GGACTCCGTACTCCTCCACT |
| PARP2 R | TGGTCCAATGGGTGTTCTGG |
| PEX10 F | TCTGCTGGGAGTGCATCAC |
| PEX10 R | CGAAGGTAGATGAGCTTCTGGG |
| POLR2H F | GACAAGTTCCGGTTGGTCATAG |
| POLR2H R | AGTGGGGTTGTATTCACCATCA |
| POLR2K F | GGAGAGTGTCACACAGAAAATGA |
| POLR2K R | TCGAGCATCAAAAACGACCAAT |
| POLR3K F | CACCCGCAAGGTAACAAATCG |
| POLR3K R | CTGCATGAAGTAAGCACGAGG |
| Pri-miR-346 F | CCTCGTCTGCAGGGCCAACC |
| Pri-miR-346 R | CCCATGCAGGAGCCGCTCTG |
| Pri-miR-222/221 F | AGAAGGCAAAGGATCACCCAG |
| Pri-miR-222/221 R | ATCTCTCTCAGGACACTGAAG |
| Pri-miR-17a/18a/19a/20a F | CCAGTCAGAATAATGTCAAAGTG |
| Pri-miR-17a/18a/19a/20a R | CAACATCAGCAGGCCCTGC |
| PTPN18 F | AGGAGTCCCGTTCTGTGTACC |
| PTPN18 R | TGCCTCACATAATCCACGGTG |
| PUM2 F | TCGGGGAATGGGAGAGCTTT |
| PUM2 R | GCTGGGACATTGAATGGTGAGA |
| PUM3 F | AGCAAGGGGACAAATCACCAA |
| PUM3 R | ACCATCTGGCTGGAATTTTCTC |
| REXO2 F | TGGCTGAAGGTCCTAACCTGA |
| REXO2 R | TGCTCCTTACACCAATCTGACA |
| RNASEH2 F | AAGACCCTATTGGAGAGCGAG |
| RNASEH2 R | AGTTCAGGTTGTATTTGACCCG |
| RORA F | ACTCCTGTCCTCGTCAGAAGA |
| RORA R | CATCCCTACGGCAAGGCATTT |
| SHLD2 F | ATGAGTGGAGGATCTCAAGTCC |
| SHLD2 R | GGGGTCAGCAACAGACATTAAAG |
| SMAD7 F | TTCCTCCGCTGAAACAGGG |
| SMAD7 R | CCTCCCAGTATGCCACCAC |
| SMC1A F | CATCAAAGCTCGTAACTTCCTCG |
| SMC1A R | CCCCAGAACGACTAATCTCTTCA |
| SMC3 F | ATTGGTGCCAAAAAGGATCAGT |
| SMC3 F | GATTGCTTCGAGAAAAACCAGC |
| SMC4 F | AGCCCAGAGGATCTTGAAGC |
| SMC4 R | ATGGCACCGAGGTTTGGTTT |
| SOX2 F | GCACATGAACGGCTGGAGCAACG |
| SOX2 R | TGCTGCGAGTAGGACATGCTGTAGG |
| SYNPO F | ATGGAGGGGTACTCAGAGGAG |
| SYNPO R | CTCTCGGTTTTGGGACAGGTG |
| TBX3 F | CTATTTCGAGCTCAGCGGCA |
| TBX3 R | TCCTTGTGCCTTGCGTTTTT |
| TOP2A F | TGGCTGTGGTATTGTAGAAAGC |
| TOP2A R | TTGGCATCATCGAGTTTGGGA |
| TSHZ2 F | GGACGAGGAGCTAGAAACGG |
| TSHZ2 R | CTCTTGATGTCCGACACCTGA |
| TTK F | GCGACGGGGAAATTCAAACG |
| TTK R | TCTACAGAAAGCTGCGCTGG |
| VSIG10L F | CTGAGGGCCAAGATTTGAGCC |
| VSIG10L R | CAGACAGTTTGGTATGGGAGAC |
| WDHD1 F | GCTGGACCTGTGGTGTCAAT |
| WDHD1 R | AGCAGTTGAACTCCAAGGCA |
| XRCC6 F | GCTAGAAGACCTGTTGCGGAA |
| XRCC6 R | TGTTGAGCTTCAGCTTTAACCTG |

1. Halliwell, J.A.*, et al.*, 2020. **54**: e115.

2. Varghese, F.*, et al.*, PloS one, 2014. **9**: e96801.

3. Dobbs, F.M.*, et al.*, 2020: 2020.2008.2025.266239.

4. Stempor, P.*, et al.*, Wellcome open research, 2016. **1**: 14.

5. Tichon, A.*, et al.*, Nature communications, 2016. **7**: 12209.

6. Lee, S.*, et al.*, Cell, 2016. **164**: 69-80.
